# What evidence exists for temporal variability in Arctic terrestrial and freshwater biodiversity throughout the Holocene? A systematic map protocol

**DOI:** 10.1186/s13750-022-00267-x

**Published:** 2022-04-04

**Authors:** Andrew C. Martin, Jakob J. Assmann, Richard H. W. Bradshaw, Mari Kuoppamaa, Niina I Kuosmanen, Signe Normand, James D. M. Speed, Marc Macias-Fauria

**Affiliations:** 1 School of Geography and Environment, University of Oxford, South Parks Road, Oxford, OX1 3QY UK; 2 Department of Biology, Ny Munkegade 114-116, 8000 Aarhus, Denmark; 3 Department of Geography & Planning, University of Liverpool Roxby Building, Liverpool, L69 7ZT UK; 4 Department of Geosciences and Geography, Faculty of Science, University of Helsinki, 00014 Helsinki, Finland; 5 Department of Natural History, NTNU University Museum, Norwegian University of Science and Technology, Trondheim, Norway

**Keywords:** Palaeoecology, Dendrochronology, Late Quaternary, Lake sediments, Environmental archives, Environmental proxies

## Abstract

**Background:**

The Arctic tundra is subject to the greatest climate change-induced temperature rises of any biome. Both terrestrial and freshwater biota are responding to recent climate warming through variability in their distribution, abundance, and richness. However, uncertainty arises within models of future change when considering processes that operate over centennial timescales. A systematic evidence synthesis of centennial-scale variability in biodiversity does not currently exist for the Arctic biome. Here, we sought to address the primary research question: what evidence exists for temporal variability in Arctic terrestrial and freshwater biodiversity throughout the Holocene (11,650 years before present (yBP)—0yBP)?

**Methods:**

Consultation with stakeholders informed key definitions, scoping and the appropriateness of the research question. The research question was structured using a PECO framework—Arctic biota (P), a timestamped year in the Holocene (E), another year in the Holocene (C), and the dimensions of biodiversity that have been measured (O)—to inform the search strategy. Search strings were benchmarked against a test list of 100 known sources to ensure a specific and comprehensive return of literature. Searches will occur across 13 bibliographic databases. The eligibility criteria specify that sources must: (a) use ‘proxy’ methods to measure biodiversity; (b) fall within the spatial extent of the contemporary Arctic tundra biome; and (c) consist of a time-series that overlaps with 11,650yBP to 0yBP (1950AD). Information coded from studies will include proxy-specific information to account for both temporal uncertainty (i.e., the characteristics of age-depth models and dating methods) and taxonomic uncertainty (i.e., the samples and processes used for taxonomic identification). We will assess temporal uncertainty within each source by determining the quality of dating methods and measures; this information will be used to harmonise dates onto the IntCal20 calibration curve and determine the available temporal resolution and extent of evidence through space. Key outputs of this systematic map will be: (1) a graph database containing the spatial–temporal properties of each study dataset with taxonomic harmonisation; and (2) a geographical map of the evidence base.

**Supplementary Information:**

The online version contains supplementary material available at 10.1186/s13750-022-00267-x.

## Background

### Rationale

The Arctic tundra biome is warming at an unprecedented rate [1, 2], which is leading to new challenges for the people and biodiversity at the Pan-Arctic scale [3]. As a largely coastal biome, declines in Arctic ocean sea ice extent and thickness are amplifying rates of increase in air temperatures [4], and changing weather patterns driving an intensification of extreme events including drought [5]. Recently observed climate-induced shifts in tundra vegetation community composition [6]—particularly shrubification [7]—have cumulative effects on birds [8, 9] and mammals [10] (such as reindeer [11–14]), and insects [15], all of which result in wider ecological perturbation [16, 17]. Without a holistic understanding of the responses of individual species to environmental drivers, it is difficult to predict near- and long-term consequences of ongoing environmental pressures.

The study of biodiversity responses to environmental change through experimentation or observation in neo-ecology [18] is essential for identifying the underlying mechanisms that may explain its variability. However, such research designs identify the role of controlling factors and responses only within their spatial–temporal frame of reference; predictive insights based on neo-ecology alone are therefore subject to extrapolation both in terms of the environmental envelope and response rates [19]. Long-term ecological information is thus key to understand present directionality and rates of change [20], with relevance for biodiversity conservation [21]. The recent period of relative climatic stability during the Holocene since the end of the Younger Dryas may elicit information to address concerns that arise from a reliance on contemporary ecological research. Time series of proxy measures for the presence or absence of species can indicate distributions [22]; migration [23, 24]; persistence [25]; and the development of refugia over time [26]. Such time-series also illustrate the rate and order of change in biodiversity metrics and given palaeo-environmental data, may thus be used to infer the mechanisms behind the observed trends [27]. When synthesised across space, hindcasting methods can also be used to test assumptions within spatially explicit ecological models [28], while process-based distribution models can indicate reasons for changes in species distributions [29]. Biodiversity metrics including species co-occurrence [30] and beta-diversity [31] may be reconstructed [32]. Research designs based on space-for-time substitution can overestimate the effect size of environmental processes [6]. Rates of change and the order of events are masked in space-for-time studies, as each site within a spatial gradient has its own unique history. Differences in biodiversity characteristics between sites within spatial gradients (e.g. of temperature) reflect the culmination of long-term integrated variability in temperature and other factors, whereas for temporal data at a single site temperature changes are reflected in biodiversity changes over a shorter term. Space-for-time studies may therefore not be appropriate for calibration and incorporation into predictive models of environmental change; this includes the use of chronosequences [33].

A systematic evidence synthesis of centennial-scale variability in indicators of biodiversity does not currently exist for Arctic biomes. Such a dataset is necessary to: (a) refine models of the causes and consequences of Arctic biodiversity change by accounting for processes that operate over decadal to centennial timescales; and (b) target resources towards evidence gaps where uncertainty in biodiversity change is greatest. Existing database efforts only partly fill these needs. The European Pollen Database and North American Pollen Database, which have been incorporated into the Neotoma Palaeoecology Database [34], contain datasets from several palaeoecological sources, but with the disadvantage of being non-exhaustive (based on voluntary contributions), nor focused on the Arctic region. The ‘Arctic Holocene proxy climate database’ (AHPCD) [35] is an expert-led, pan-Arctic synthesis of palaeoenvironmental proxy records from lake sediments and other sources, stemming from a non-systematic search strategy. A subset of the sources included in this database indicate the temporal dynamics of biodiversity measures (i.e., those using biotic proxies, which includes but is not limited to pollen and chironomids). The quality of dating methods used was quantified in AHPCD in a systematic manner by assessing the age-depth models of each included sedimentary record against defined quality metrics. Both Neotoma and the AHPCD incorporate full datasets (rather than only metadata), where willingness or ability to contribute may be dependent on many factors. We therefore may not have a complete record of research from which data have yet to be digitised or made available under an open license.

Regional reviews and data syntheses have also been completed, albeit scoped to specific proxies. For example, for Eurasia a synthesis of pollen and plant macrofossil datasets above 40°N includes some Arctic sites [36]. Palaeoclimate databases that indirectly contain biodiversity information have been compiled for North-Western [37] and North-Eastern [38] America. Additional palaeotemperature reconstructions based on biotic proxies are captured in a global Holocene palaeotemperature database [39]. Taxon-specific Arctic databases exist for diatoms [40] and for fossil insects (e.g., limited to North-West Siberia [41]), amongst others.

The proposed systematic map has four important contributions to Arctic biodiversity research. First, it will contribute a holistic quantification of uncertainty along three axes—taxonomic, temporal, and spatial—to enable the existing evidence base to be applied within models of centennial-scale variability, such as those integrating climate, soil, or landscape variability with biodiversity outcomes. Second, it will identify evidence gaps along spatial and temporal axes and subsequently allow assessment of research priorities to reduce the identified spatial–temporal uncertainties arising from these gaps. Third, it will indicate the quality of underlying data available for use in ‘hindcasting’, a practice through which species distribution models may be validated against previous species presence-absence data [42]. Fourth, it will capture the investment that has occurred in the collection, analysis, and storage of samples (e.g., sediment cores), which can help guide future efforts to extend the knowledge base in the most cost-effective manner by highlighting reserves of data that have been already collected. Fieldwork collection and radiocarbon dating of fossil material requires substantial resources, and preparation and analysis of fossil material can take many years. There are substantial reserves of ‘dark data’ [43] within the disciplines of palaeoecology and archaeology where the resultant datasets have not been published.

There are specific challenges facing the integration of centennial-scale biodiversity indicators. First, long-term ecological data often arises from environmental archives or samples that have uncertain temporal and spatial frames. Sediment cores are commonly dated using radiocarbon dates at a series of depths; an age-depth model is then required to calibrate depth to age, which may be constructed using classical or Bayesian methods [44]. The distribution of dates, the age-depth modelling approach employed, and the nature of the material all contribute to (variable) temporal uncertainty around proxy measures from all levels within the core [45]. Additional complexity arises from the calibration of radiocarbon dates; as radiocarbon calibration curves are updated with new information, previously estimated ages (in years before present—yBP) may diverge substantially from new estimates [46]. It is therefore necessary to recalibrate radiocarbon dates to a common standard when comparing across studies. Second, spatial uncertainty arises from the movement of dead/fossil material from source to sink locations (e.g., driftwood, microfossils in lake sediments). Driftwood is found around Arctic shorelines that can be dated to the early Holocene [47], but the location is only partially or not indicative of the tree’s original locale. Similarly, pollen deposited in lakes and found in the sedimentary record has differential origin from a source area depending on the size and shape of the lake basin, hydrological and weather conditions, surrounding habitat, and the dispersal traits of each pollen morphotype [48, 49]. Third, taxonomic uncertainty exists during the establishment of a link between a palaeoecological proxy and the original taxonomic unit (i.e., species, genus, family). For example, proxy studies that apply morphotype classification to taxonomic groups such as phytoliths, plant macrofossils, ostracods, and pollen require implicit or explicit use of keys or atlases to connect individual morphotypes to one or many candidate taxa [50]; the robustness and reproducibility of the method determines the certainty and temporal stability (i.e., conversion between previously accepted synonyms and presently accepted names) of the identification. Finally, much older research has generated proxy data that may now be used to infer biodiversity characteristics, but where the primary research focus at the time was paleoclimate reconstruction (e.g., climate reconstructions inferred from biological remains/assemblages) [51]. A robust approach to synthesising palaeoecological data should account for the temporal, spatial, and taxonomic uncertainties outlined here.

To address the identified need for an assessment of the current evidence for centennial-scale variability in measure of biodiversity and associated evidence gaps, we prepared a systematic map protocol adhering to the ROSES checklist for systematic map protocols [52].

### Stakeholder engagement

The identification, engagement with, and role of stakeholders throughout the entire mapping process was designed referring to the framework for stakeholder engagement in environmental management [53]. We employ a broad definition of stakeholder to include any party who may use the information collated within the systematic map, which includes (but is not limited to) government/non-government decision-makers, Arctic inhabitants, museums and educational institutions, and the researcher community.

The planned role of stakeholders within the mapping process is to: (1) validate the proposed scoping of the review and research questions; (2) scrutinise the design of the search strategy; and (3) identify and facilitate access to grey literature sources (e.g., through identification of key contacts, formats, and locations of unpublished material). Where stakeholders make substantial contributions, consent will be sought to include them within acknowledgements or credited within authorship of the final map.

### Stakeholder Identification (analysis and selection)

**Fig. 1:**
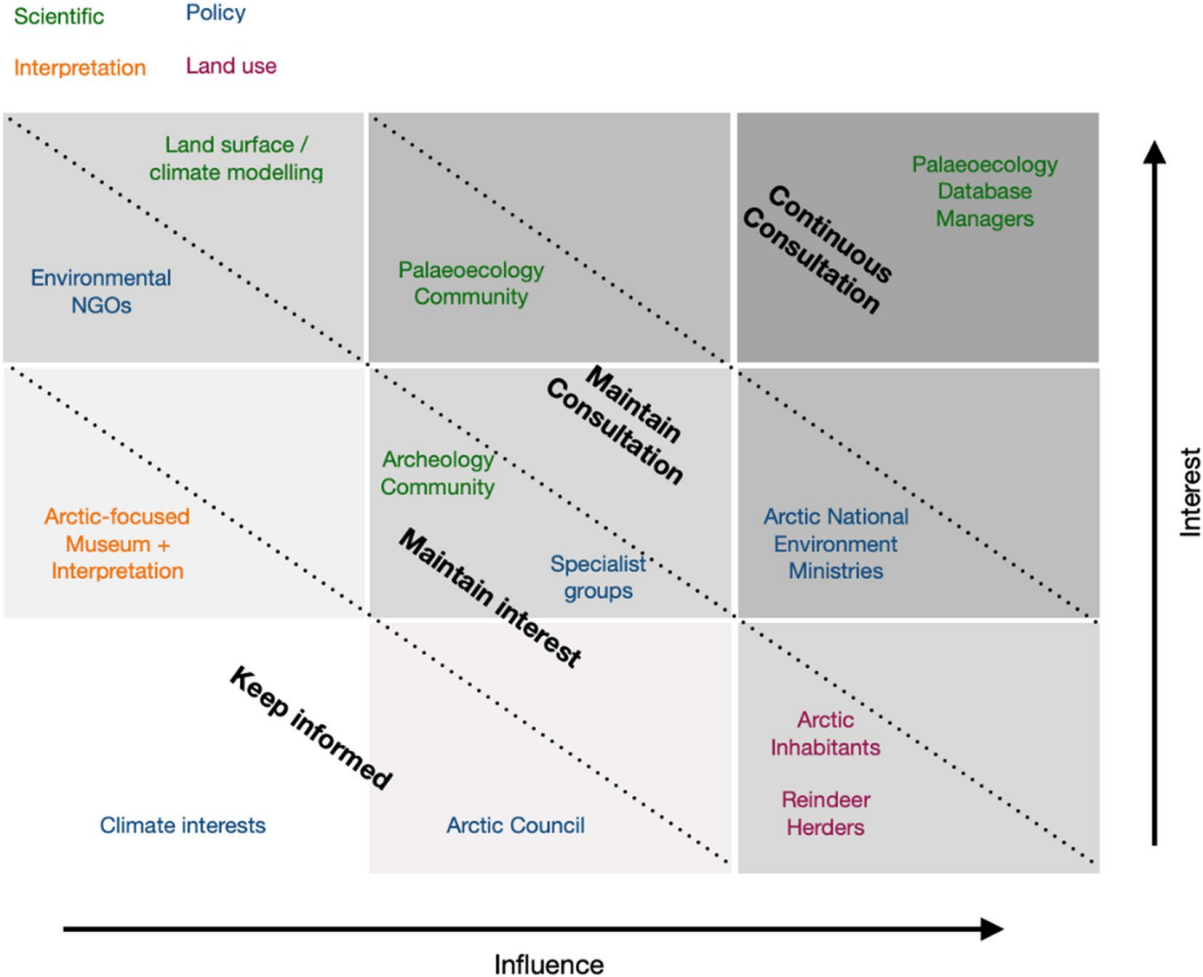
Stakeholder influence/interest matrix for the centennial-scale Arctic biodiversity map. Constituent groups within broad groupings (differentiated by colour) are overlain with the identified communication strategy during stakeholder analysis

The following groups of stakeholder groups were identified by the review team: Environment departments of Arctic Governments; county authorities who manage nature reserves; inter-Governmental organisations [e.g., Arctic Council who produce biodiversity assessments alongside CAFF (Conservation of Arctic Flora and Fauna)]; Arctic inhabitants; scientists involved in production (palaeoecologists, archaeologists) and use (climate modellers) of long-term ecological research. Identified stakeholder groups were placed by the review team within an influence-interest matrix to determine appropriate levels of engagement throughout the entire process (Fig. 1). Communication strategies were tailored to specific groups depending on their position in the interest-influence matrix in Fig. 1. Engagement methods were tailored according to the communication strategy, as stated in the following section.

A mixed *stakeholder selection* process was used, including both *purposive selection*—which involves the identification of well-known stakeholders [53]—and a systematic approach, which involved searching a published list of organisations, described below. For *purposive selection*, the proposed systematic map is a deliverable defined within *Drivers and Feedbacks of Changes in Arctic Terrestrial Biodiversity* (CHARTER), an EU Horizon 2020 research consortium. CHARTER has a steering committee, which includes organisations and individuals who represent core stakeholders. In addition, the consortium contains parallel research streams who intend to use the output of this systematic map in further research. Both the CHARTER steering group and CHARTER work package members—which represent the interests of reindeer herders, scientists, funders, and Government—were included through purposive selection. Furthermore, a systematic approach was applied to identify further relevant stakeholders from within the index of ‘who’s who’ acronyms from the Association of Polar Early Career Scientists (https://www.apecs.is/research/who-s-who-polar-acronyms.html accessed 4th January 2021). The APECS acronyms database is tagged with research areas; we used an advanced search to include acronyms with the following tags: *Natural sciences; Social sciences; Indigenous; Indigenous knowledge; Polar sciences; Biology; Policy; Multidisciplinary; Conservation; Environmental Protection; Climate; Geosciences; Outreach; Environmental; Biodiversity; and Scientific Research*.

#### Initial invitation and engagement

**Fig. 2:**
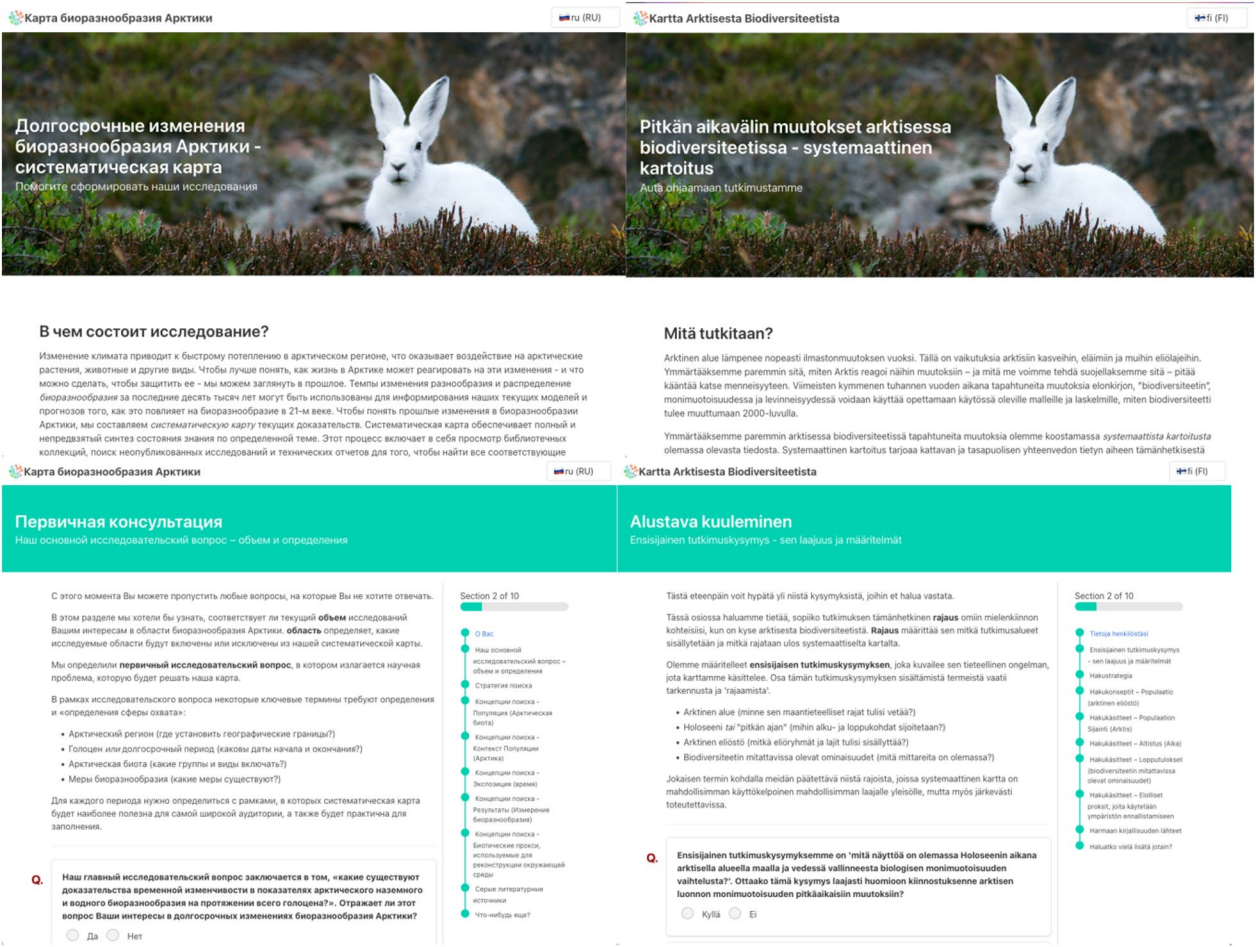
Online consultation platform as used for this project. The consultation was made using the *cottongrass* consultation platform [54] and was conducted in four languages. Left: Russian; Right: Finnish

We used an initial period of engagement from 12th July 2020 to 20th September 2021 (10 weeks) to receive stakeholder input on question formulation (including scoping and definitions) and the formulation of the systematic map protocol. Stakeholders were invited to express an interest in the map, with the option of providing comment on the scope and research question through a multi-lingual website built with the *cottongrass* platform [54], which defaulted to the in-country language of the users’ web browser if available (Fig. 2). Invites were made by email to contribute to an online consultation. The wording of the invite was tailored to each stakeholder group (for wording see Additional file 1). Although only selected stakeholders were contacted, the online consultation was open and invited stakeholders were encouraged to share the link to other interested parties that they were aware of (*snowballing*). The initial consultation was conducted with full-text professional translations in English, Finnish, Russian, and Swedish. The format of the online consultation is given in Additional file 2. Stakeholders who represent organisations were invited to circulate the consultation to their members. Invites also gave stakeholders the option to contact us by other methods if they preferred, such as video or voice call.

The initial engagement considered the following topics to inform protocol development. First, we sought to understand the research experiences of scientists who have been involved in the production of palaeoecological datasets, as well as the experiences (e.g., successes or difficulties) of those who have, or have an interest in, applying these data to policy, research, and other use-cases. This includes experiences addressing the three key uncertainties when integrating palaeoecological datasets (i.e., spatial, temporal, and taxonomic uncertainty). Second, we sought to scope key definitions (as used in inclusion/exclusion criteria) to include only information relevant to stakeholder groups; these include definitions for ‘biodiversity components’ and the ‘Arctic region’. Third, we sought to assess the suitability of the wording of the primary research question. Fourth, we requested support and approval for the search concepts and terms used in the proposed search strategy.

#### Explanation to stakeholders

Tailored explanations of systematic mapping concepts and the reasons for choosing this method for the present research were included with the initial invites to stakeholder groups. Key definitions were initially determined by the protocol working group in a drafting workshop prior to initial engagement with stakeholders (see Sect. "Objective of the systematic map").

#### Continued engagement and measuring impact

Engagement with stakeholders will continue beyond the initial contact in line with the levels in the interest-influence matrix (Fig. 1). For the lowest two tiers (‘keep informed’ and ‘maintain interest’) every other month a newsletter will be prepared to present the status of the mapping process and advertise calls for grey literature. For those in the ‘maintain consultation’ group, two-way communication will be encouraged to ensure engagement in the map process, including sharing of articles, data, and other grey literature sources. All stakeholders will be made aware of the stage of the mapping process and expected quiet times during screening and study coding.

#### Dissemination and communication

The finished map will be submitted to *Environmental Evidence* and publicised to stakeholders using a multilingual press release as part of the CHARTER consortium. An online visualisation and a data exploration tool will be made openly available and shared with all identified stakeholders, which will contain a plain-language story summary of the key findings (detailed further in Sect. 4.5).

## Objective of the systematic map

The primary question of the systematic map is: ‘what evidence exists for temporal variability in Arctic terrestrial and freshwater biodiversity throughout the Holocene?’ The research will also address two secondary research questions, which address the anticipated sources of heterogeneity in the measures of biodiversity:

1. What is the quality of the dating methods used, and how is this reflected in temporal uncertainty within the synthesised evidence base?
2. To what extent is the identified evidence for long-term variability in biodiversity presented alongside co-occurring indicators of environmental variability from the same environmental archive?

We defined core terms contained within the primary research question from which scoping of the study boundaries could then be conducted. The core definitions included: (a) components of biodiversity; (b) measures (or dimensions) of biodiversity; (c) long-term ecological sources/environmental archives; (d) the regional bounds of the Arctic; and (e) the earliest and latest dates comprising the Holocene within the defined Arctic region.

- We defined the *Components of Biodiversity* as the variability in both living organisms (taxonomic, genetic, and morphological) and the ecological complexities of their interactions following the 1992 Convention on Biological Diversity [55].
- *Measures of biodiversity* are the richness, diversity, and evenness of the components of biodiversity at a single point in time (e.g., species richness, genetic diversity); this includes the presence or absence of individual species. The properties of biodiversity may also be measured by their spatial distribution (i.e., alpha, beta, gamma, and zeta-diversity), and across multiple time-points by spatial–temporal stability in terms of rates of change and periodicity (e.g., alpha-variability, gamma-variability, synchronicity) [56]. Further derived measures include the resilience and persistence of biodiversity over time [57].
- *Long-term ecological sources* are defined as information prior to the modern observational record that observe proxy data and apply specific methods for their analysis. Proxy methods therefore include any method where implicit or explicit inference or reconstruction is required to relate the proxy measure to a species or other constituent component of biodiversity. Under this definition, long-term ecological records include evidence from environmental archives within lake and peat sediment cores (e.g., fossils such as plant macrofossils, pollen; and inference from physical characteristics such as organic content, stable isotope ratios), reconstructions from tree and shrub wood rings, and terrestrial fossils (e.g., vertebrate bones, (sub)fossil wood).
- We consulted with stakeholders to identify a suitable definition of the *Arctic region*, to address issues such as whether transition zones and surrounding taiga should be included within the research context. The *Arctic region* is often defined by either: daylight hours during the summer and winter solstices (Arctic circle); mean annual temperatures, often the 10℃ isotherms; north of a fixed latitudinal band; or based on biome (e.g., tundra north of the tundra-taiga ecotone). Although the boundaries of the Arctic region may have varied through the Holocene based on some of these definitions, our research question is concerned with the contemporary Arctic.
- We define the Holocene in the Arctic is as the onset of warmer conditions at the end of the Younger Dryas. We set the date for when the onset of warming occurred as 11,650yBP, which is the *Global Boundary Stratotype Section and Point* that marks the end of the Pleistocene and start of the Holocene epoch [58].

**Fig. 3:**
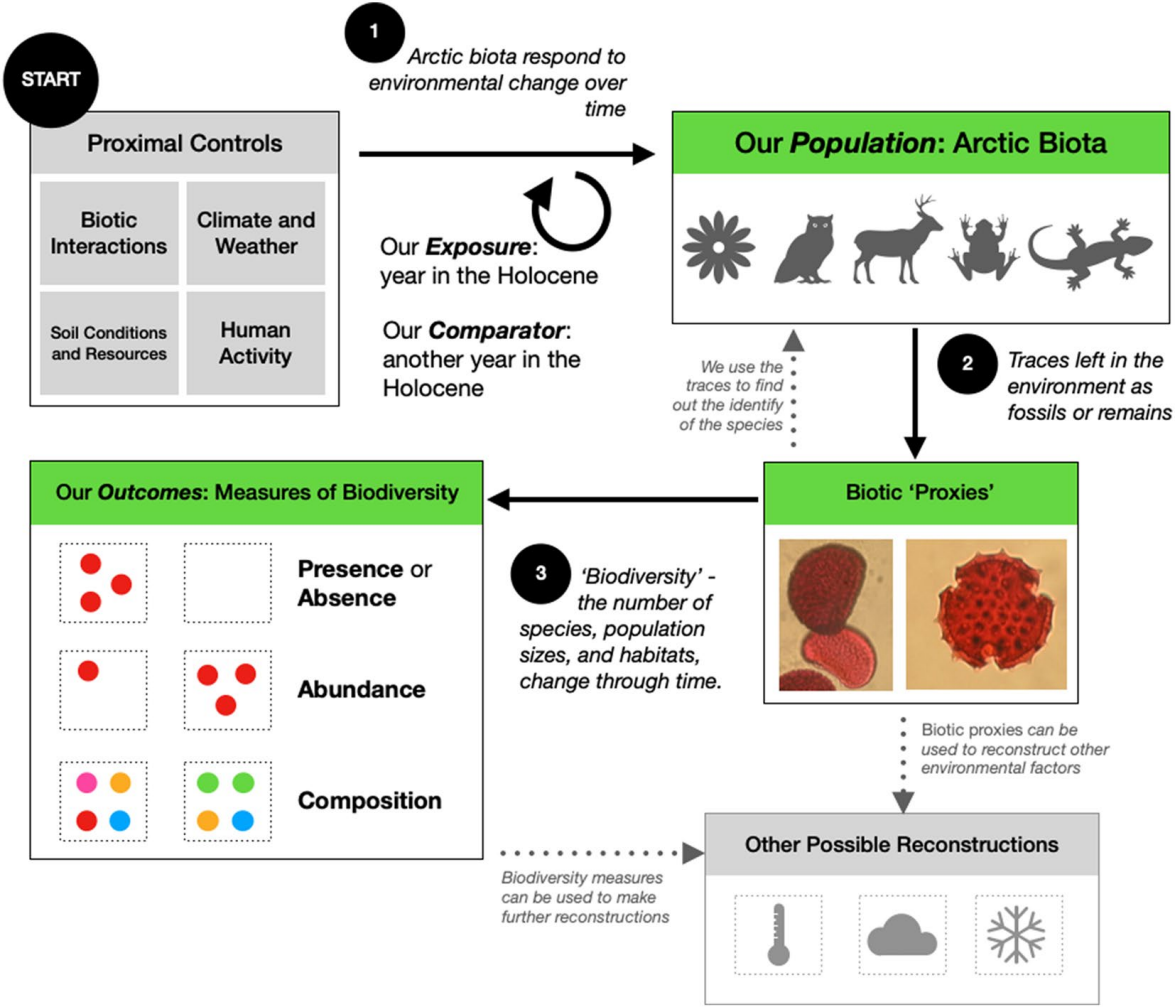
Theory of Change. In Arctic and Oro-Arctic regions, biota (defined by morphological taxonomy—‘*population*’) has been *exposed* to the progression of time, over which time unspecified (possibly currently unknown) proximal controls varied in importance and magnitude. Temporal variability within the proximal controls (both biotic—such as inter-specific competition—and abiotic—such as soil physical characteristics), which is driven ultimately by other large-scale environmental factors (e.g., sea ice, teleconnections) affects the performance of biota year-to-year. Biota deposit remains and other indicators of past existence; the centennial-scale variability in Arctic biota (*population*) are therefore now recorded within measures of biodiversity (*outcomes*) of the fossils and remains of the *population*

We developed a theory of change to model the research question through a Population-Exposure-Comparator-Outcome (PECO) framework (Fig. 3). The PECO defines the relationship between the exposure and outcome where there has not been a specific intervention [59]. The PECO is defined using the definitions (a) to (e) above. Within the Arctic region, components of biodiversity (e.g., species—P) vary from one year in the Holocene (E) to another year (C), as indicated by measures of biodiversity (O). The changing presence/absence, distribution and abundance of Arctic biota are controlled by proximal environmental factors (i.e., environmental processes that directly affect individuals, such as biotic interactions and local climate/soil conditions) over the long-term (i.e., at centennial-scale), which are themselves the product of ultimate controls (i.e., further indirect sources of environmental variability, such as Arctic sea ice extent) [60]. Here, we treat time as an integrated measure of all environmental variability, as we do not wish to define a priori the proximal controls to all biodiversity and assume that little is known between the exposure (time) and the outcomes (measures of biodiversity), such as the nature and functional form of any relationship. Rates of change are therefore captured by the movement of time, with every year within the Holocene capturing the synchronicity of responses across space (C). Rates of change are based on background environmental variability and expressed as the spatial–temporal covariance of the measures of biodiversity. Throughout the Holocene, the components of biodiversity are identified using long-term ecological proxies, which introduce added complexity through the implicit or explicit identification method of biota and environmental proxies. The population is therefore not directly observed; we represent the link between proxies for the population and the population itself through methods used to infer biotic components from each proxy [61].

### Scope

The key definitions and theory of change were scoped through initial stakeholder engagement to establish boundaries for the research question. Definitions were scoped to the following:

- Components of Biodiversity. Taxa from within both terrestrial and freshwater environments will be included; taxa from littoral environments are also included, although marine taxa are excluded. Included taxa are limited to the kingdoms Plantae (which includes bryophytes) and Fungi (which includes lichens); the phylums Bacillariophyceae (diatoms) and Mollusca (molluscs); the superclass Tetrapoda (which includes mammals, birds, amphibians, and reptiles) but excluding humans; the classes Ostracoda and Coleoptera; and the family Chironomidae. Consequently, other taxonomic groups (e.g., most insects) are not included. Taxa may be identified through morphological or molecular techniques (e.g., sedimentary DNA). Aside from taxonomic diversity, habitat diversity is included (e.g., land cover change, for example shifts in the dominance of forest versus grassland); morphological diversity is excluded.
- Measures of Biodiversity. No exclusions to scope were identified, but concepts were identified as—but not limited to—richness; evenness (which employs measures of relative abundance between taxa); and presence-absence. When measured through time, presence-absence may implicitly indicate colonisation, establishment or establishment limits, persistence, local loss of taxa, and successional patterns. When measured across sites, geographical distributions may be reconstructed. Habitat diversity is sometimes represented in high-latitude research by the position of the northern treeline, the position of tundra-taiga ecotone, and habitat zonation.
- Long-term ecological information. No limits to scope are included above the core definition. For clarification, long-term ecological information not based on proxy-based reconstructions are excluded; this includes herbarium specimens collected from living plants, and oral histories.
- Arctic region. Through the initial consultation, stakeholders placed importance on the inclusion of the Scandinavian high-altitude tundra, as these contain many known grey literature sources of palaeoecological data and are of relevance to contemporary research questions concerning reindeer management. A preference to include the contemporary tundra-taiga ecotone was identified. There was a preference for using a latitudinal band to define the Arctic region. To incorporate these considerations, we limit the Arctic region here to include areas that are either (a) within bioclimatic subzones A—E of the contemporary Arctic as defined by the Circumpolar Arctic Vegetation Map [62]; (b) within the ‘sub-Arctic’ as defined by the Arctic Biodiversity Assessment [63]; or (c) north of the boundary line of the Conservation of Arctic Flora and Fauna (CAFF) working group of the Arctic Council [63]. The CAFF boundary is defined by each state; e.g., for Sweden, it includes reindeer husbandry areas. The CAFF boundary represents a compromise that mostly follows the Arctic Circle in Fennoscandia, allowing for a latitudinal band definition in this region, but flexing to include sub-Arctic regions in Canada and north-eastern Russia, where the sub-Arctic zone is of far lower latitude.
- Holocene. The Holocene was scoped to truncate the recent past; information that only includes times from 1950AD to the present day are excluded to avoid research focused on contemporary ecology and land surface change, for example using remote sensing proxy methods; such studies do not concern long-term variability as defined in our research question.

## Methods

### Searching for articles

The search strategy was designed to minimise publication biases arising from exclusion of unpublished sources and maximise sensitivity (comprehensiveness) and specificity in locating relevant sources. A multi-lingual strategy will be used to minimise publication bias. Searches for both published and unpublished sources will be conducted in the appropriate language(s) for the bibliographic index in each database. The 13 bibliographic databases that we will use to identify publicly available sources are outlined in Table 1:

**Table 1:** Databases used in search strategy for published sources

Type	Bibliographic database	Language of search string(s)	Source
Formal	Web of Science Core Collection	English	https://www.webofscience.com/wos/woscc/basic-search
	Russian Science Citation Index	English, Russian	https://www.webofscience.com/wos/woscc/basic-search
	Zoological Record	English	https://www.webofscience.com/wos/woscc/basic-search
	CAB Abstracts 1910–2021 Week 31	English	https://www.cabdirect.org
	BIOSIS Previews Archive (1926–1968); BIOSIS (1969–)	English	https://ovid.com; https://www.webofscience.com/wos/woscc/basic-search
	Bielefeld Academic Search Engine	English	https://www.base-search.net
	Scopus	English	https://www.scopus.com
	ProQuest	English	
	Cristen	Norwegian	https://app.cristin.no/
	Swepub	Swedish	http://swepub.kb.se/form_extended.jsp
	Reserch.fi	Finnish	https://www.research.fi/en/
	Bibliotek.dk	Danish	https://bibliotek.dk
	Biodiversity Heritage Library	English	
Informal	Google Scholar	English	https://scholar.google.com

Search term concepts were identified by taking the key concepts from the PECO and definition of terms outlined in the previous section. For each concept, we identified synonyms and preferred terms within CAB Thesaurus [64]. In addition, we also used CAB Thesaurus to identify both broader terms that still fell within the scope of the research question, and narrower terms that may increase the sensitivity of the search string. We assessed each term for double meanings that may lead to irrelevant search results. We also identified how concepts varied between disciplines, for example between ecology and archaeology, and included variations within the search term concepts. For example, time intervals within the Holocene are defined differently depending on geographical context, discipline, and dating method (e.g., palaeoecology pollen zones, archaeological periods, geological periods). The search concepts and terms used are given in Additional file 3.

Search terms were constructed into search strings in appropriate syntax for each database. For BIOSIS, the specialist taxonomic, geographical, and temporal indices were searched for the population, context, and exposure respectively. Search strings are also given in Additional file 3.

We assessed the sensitivity (comprehensiveness) of the search string by comparing the search result against a test list of 100 benchmark publications that address the research question, match the eligibility criteria, and represent a broad spread of content across the project scope. The test list was compiled with contributions from each member of the mapping team and from stakeholders to minimise bias towards specific subject areas. The test list is given in Additional file 4. We found that 98 of 100 articles were correctly identified by the search string (when searching Web of Science, CAB Abstracts, BIOSIS, Scopus, and the Russian Citation Index on 12th August 2021 using Bodleian library subscriptions). Of the two articles not correctly identified, one was an introduction to a special issue [65], which did not refer to biodiversity measures in its title or abstract. The papers referred to within the special issue were however identified in the test search. The second article [66] was a wide review of Quaternary megafauna extinctions without reference to geography. We did not adapt our search terms to include these two articles as they relate to broad overview material where the original sources appear to be already included, each would require the removal of a PECO element from the search terms, and we have already included adaptations to capture syntheses/meta-analyses on global datasets. We assessed the specificity of the search string by taking a random set of 250 distinct sources from the same search and assessing them against the inclusion criteria at title-abstract level; this indicated an approximate specificity at title-abstract of 9.6% (24/250 included).

When conducting searches, we will proceed through each bibliographic database search in the order of Table 1. Results will be de-duplicated in EndNote using the systematic method given in Bramer et al. (2016), accounting for differences in page number formatting between databases [67]. If the review becomes stale during the process, we will conduct search updates; we will do this at every 6-month interval that lapses since the initial search. If a source contains secondary information (e.g., meta-analysis), we will use snowballing to retrieve and assess original sources. We will not use reverse snowballing.

We will locate grey literature using three approaches. First, we will conduct web searches using a web scraping tool and the Bing and Google search engines, ensuring that account tracking influence on search results is minimised. We will assess the first 200 results of each search for research papers, books, periodicals, and reports by the identification of key words. Second, we will use snowballing from stakeholder contacts to ascertain individuals who may hold unpublished material. Third, we will conduct targeted searches on the organisational websites of nine organisations that hold Arctic- and palaeoecological-specific datasets and were identified as key sources of published and unpublished research data, as shown in Table 2.

**Table 2:** Organisational websites that will be investigated using targeted search

Type	Organisation	Website address
Palaeoecological	Neotoma [34]	https://neotomadb.org
	National Oceanic and Atmospheric Administration (NOAA) Palaeoclimatology Database	https://www.ncdc.noaa.gov/data-access/paleoclimatology-data
Arctic-specific	Arctic Data Committee	https://arcticdc.org/products/partner-data-products
	Sustaining Arctic Observing Networks	https://arcticdc.org/about-us/saon
	The Arctic Traits database	https://www.univie.ac.at/arctictraits/
	The Arctic Data Center	https://arcticdata.io/
	National Institute of Polar Research, Japan	https://www.nipr.ac.jp/english/database/
	Arctic Monitoring and Assessment Programme	https://www.amap.no/

## Article screening

### Screening process

Sources will first be screened at combined title-abstract level; those included will then be screened at full-text level. Screening will be undertaken using Colandr [68]. Articles will be screened first based on the eligibility criteria stated below at the title and abstract level simultaneously. When uncertain, articles will proceed to be screened in a second stage at full-text level. Initially, sources will each be reviewed by two individuals. To ensure consistency and objectivity in decisions but optimise resources, we will assess Cohen’s kappa κ [69] between reviewers on 250 random sources to ensure that each new individual added has moderate or greater agreement with existing reviewers in decision-making (kappa score above 0.60). Group discussion will be used to resolve differential understanding of the eligibility criteria and reach consensus on a decision for each article and, if the κ threshold of 0.60 had not been met, the process re-run on another random set of 250 sources. Only when the kappa threshold is met will sources then be reviewed by a single individual. Kappa scores will be re-assessed between all individuals after every 5,000 items on 250 new items to ensure continued consistency, which will result in at least 5% of all articles screened by two people at title-abstract level. After single screening at full-text level, 10% of articles will be double-screened and consistency assessed using Kappa scoring; if a threshold of 0.60 is not met, all articles will be double-screened. We will provide a full table of articles that were excluded at full-text level with the reasons for each articles’ exclusion linked to the eligibility criteria.

We will ensure procedural independence by ensuring that reviewers will not make decisions (i.e., include or critically appraise) their own work.

### Eligibility criteria

We will apply *eligibility criteria* to determine whether articles should proceed to the data coding stage. The elibility criteria consist of nine requisites summarised in Table 3:

**Table 3:** Eligibility criteria used in screening process

	Criterion	Rule
Inclusion	[P] Includes taxon	Must include at least one taxon as defined in 2 (a)
	[P—context] Is in Arctic region	Sample location must intersect polygon as defined in 2 (b)
	[E] Includes time in Holocene	Must include data from at least one year between 11,650yBP and 0yBP (1950AD)
	[C] Includes second time in Holocene	Must include data from a second year (forming a time-series at a single location) between 11,650yBP and 0yBP (1950AD)
	[O] Has biodiversity measure	Must include a measure of biodiversity for taxa at time-points
Exclusion	Is not proxy method	Exclude if not using proxy method as defined in 2(c)
	Is experimental design	Exclude if based on an experimental study design only
	Is oceanic	Exclude if location is in ocean (but include littoral locations)
	Is space-for-time study	Exclude if space-for-time design (e.g., phylogeographical analysis, chronosequence)

**Fig. 4:**
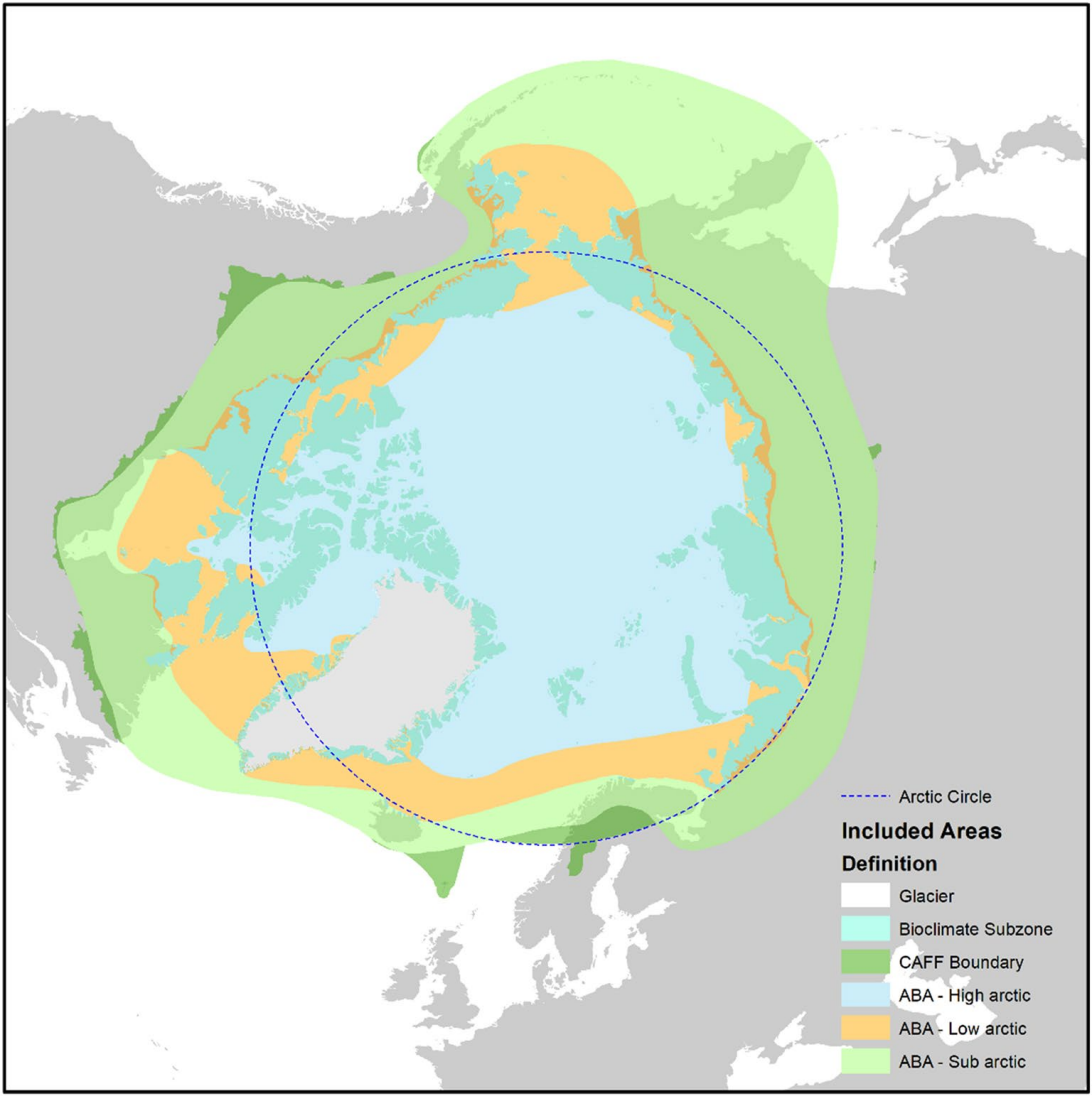
Map of the ‘Arctic Region’ as defined for this systematic map. Included areas are defined by either **a** bioclimatic subzones A–E, **b** the sub-Arctic as defined by the Arctic Biodiversity Assessment (ABA), or **c** areas below the sub-Arctic but north of the Conservation of Arctic Flora and Fauna (CAFF) boundary

Each source must include five components. First, a *relevant subject(s)* must be included, which is defined within the scoped definitions of ‘biota’ (see Sect. "Objective of the systematic map"). Second, site(s) must fall within the Arctic region as defined in Sect. "Objective of the systematic map". For site(s) near boundaries or within geographical designations that cross boundaries, coordinates must intersect with a shapefile of the valid extent (defined as shown in Fig. 4). Third, the *relevant subject(s)* must be *exposed* to: (a) two or more time points in a time-series representing temporal variability (that overlaps with the temporal extent defined as the Holocene in Sect. "Objective of the systematic map"). For clarification, the definition of temporal extent uses the original study’s dating method and dates rather than reinterpreted dates (i.e., using a newer radiocarbon calibration curve). Fourth, one or more measures of biodiversity (outcomes) defined in Sect. "Objective of the systematic map" must be measured at the time points from (3). We will also apply the following *exclusion criteria*. First, certain study designs will be excluded: experimental study designs; and space-for-time designs in which observations of the subject in geographical space are projected to variation in time using a modelling approach (e.g., phylogenetic analysis of modern population genetic structure). Second, ocean sediment cores and other non-terrestrial sources of information will be excluded. Third, the subject(s) must be identified using a ‘proxy method’ with the meaning given in Sect. "Objective of the systematic map" (c).

### Study validity assessment

We will assess the quality of the underlying data to understand the validity of individual studies and the synthesised evidence base. Of particular focus is temporal uncertainty. Recalibration of radiocarbon dates is essential to ensure comparability between ages from different studies, as older studies may use old methods of age-depth model construction and outdated calibration curves [70]. As all information included in the systematic map will consist of individual time-series, we will adapt the approach used by Wang et al. (2019) to compute consistent age-depth models with estimates of temporal uncertainty over many palaeoecological datasets simultaneously. For biodiversity measures obtained from proxies within the sedimentary record (i.e., lake sediments, peat cores), we will quantify the uncertainty present within the dating methods and age-depth models used. We will use OxCal version 4.4 [71] to recalibrate all radiocarbon dates to the IntCal20 radiocarbon curve [72] and recompute age-depth models with outlier models (to identify problematic dates) based on the recalibrated dates, which will include the calculated temporal uncertainty. The unified age-depth models will be presented alongside the original estimated dates within the final evidence map and database. For non-sedimentary records that have been radiocarbon dated, the same strategy of recalibration will be employed. The uncertainty present within the dating methods is a crucial determinant for the viability of using the records in further meta-analysis.

To quantify taxonomic uncertainty, we will compare the taxonomic labels assigned to taxa within individual research articles to working checklists of Arctic taxonomies. We will only assess taxonomic uncertainty for plants. We will use the ‘World Flora Online’ taxonomic backbone v.2019.05 (derived from The Plant List v1.1), which represents the most complete global taxonomic working list of plant names and synonyms [73].

The information from uncertainty quantification will be used in synthesis when determining the temporal resolution, extent, and continuity of available evidence.

### Data coding strategy

Data coding will be undertaken by a single person for each article. To check consistency, a random subset of 5% of sources will be coded twice; any deviations from the data coding strategy (e.g., due to human error) will be addressed or explained with annotation. Metadata from each included article will be coded and standardised into a custom graph database structure. The graph database relates metadata on literature sources, individual study timelines, taxonomical group, measures of biodiversity, and proxy methods. We will use a custom-designed form user interface to input data directly into the graph database structure; this approach was chosen to minimise redundancy and input errors when compared to using spreadsheets. The interface contains in-built data constraints so that only valid data for each field may be entered. The interface is a.NET 6 application, which is underpinned by a no-SQL data store of JSON files and indexes; this allows for data coding to be conducted by multiple people while using version control to handle conflicts. A git repository will be used to commit and synchronise work between collaborators. Source code for the graph database structure is available at Zenodo [74]. Where the source’s information is only in graphical format, we will digitise the graphics to retrieve any relevant metadata (e.g., from pollen diagrams). Where relevant information in the source is unclear, we will contact the corresponding author by email for clarification and pursue phone contact if email is unsuccessful.

**Fig. 5:**
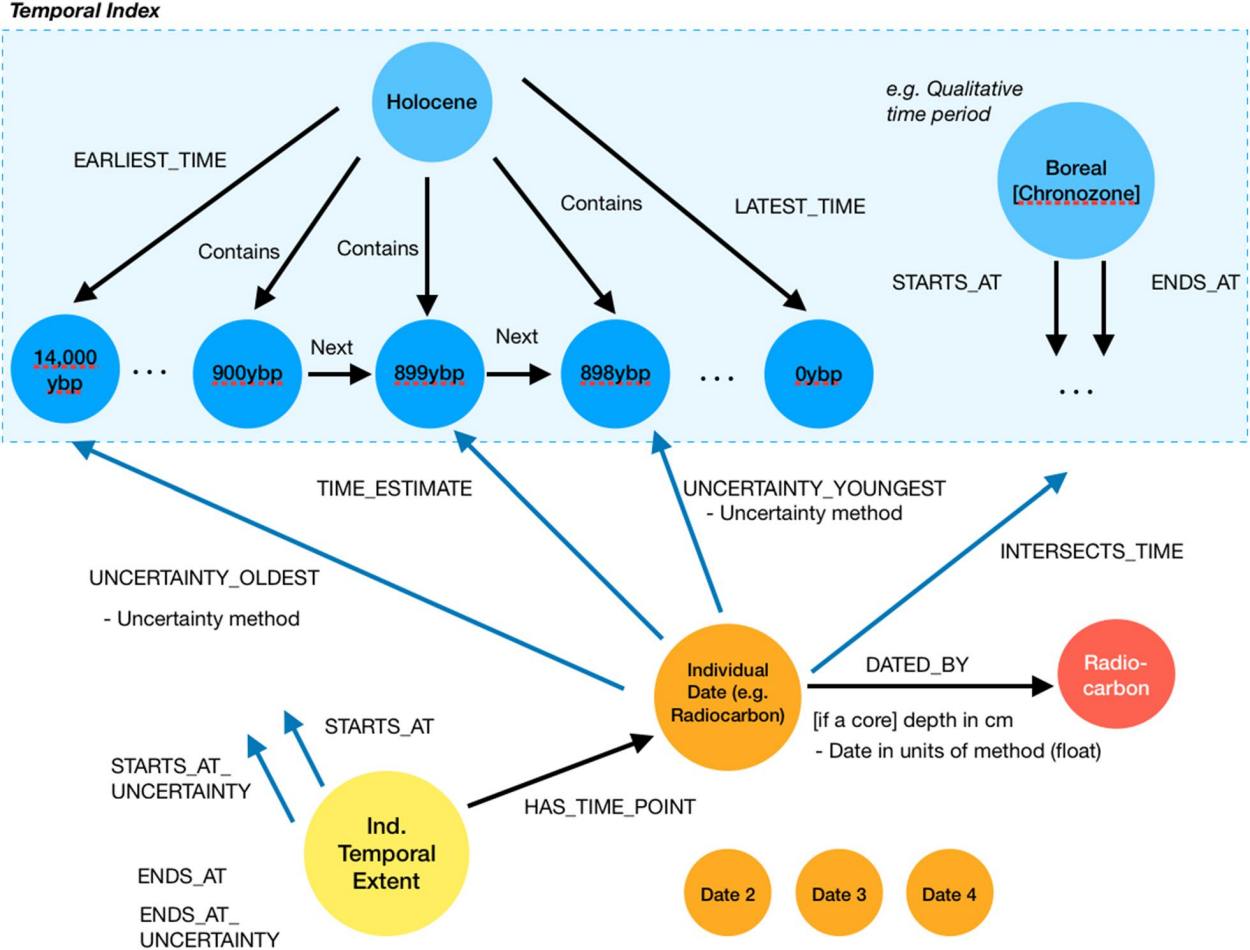
Diagram showing how coded temporal metadata is represented within the graph structure. Circles and arrows represent nodes and links respectively. Blue nodes are a common time index; orange nodes represent individual dates within studies; and yellow nodes represent the timelines within individual studies that arise from individual dates. All dates are defined in years before present (yBP), where 0 = 1950AD

For each included study, we will identify individual study timelines (i.e., time-series that may be assessed independently from others); for each timeline we will record the start and end date as given by the author, plus each individual date used to construct the time-series. All dates are specified in years before present (with upper and lower age uncertainty estimates if stated) and record the dating method used. If the date is taken from specific material (e.g., at a specific depth in a sediment core), this metadata will be recorded. The maximum temporal resolution captured is one calendar year. The data coding structure also allows for the use of qualitative time periods; where dating is referenced to a time window or interval (e.g., chronozone, pollen zone), this will also be recorded as an intermediate link between the start/end dates and the dated sample. This approach allows for sources using pollen zones of uncertain start/end dates to be included in the database. Within the graph representation of the coded data, all dates are connected to a common temporal index (Fig. 5).

For each included study, we will record the spatial location from which the sample(s) were taken, the date of sampling if known, and standard bibliographic metadata. We will record location as either coordinates of site(s) (latitude, longitude based on WGS 84 (EPSG:4326) in decimal degrees), or the name of the site, locale, region, and/or country, depending on the resolution of spatial reference given in the study.

**Fig. 6:**
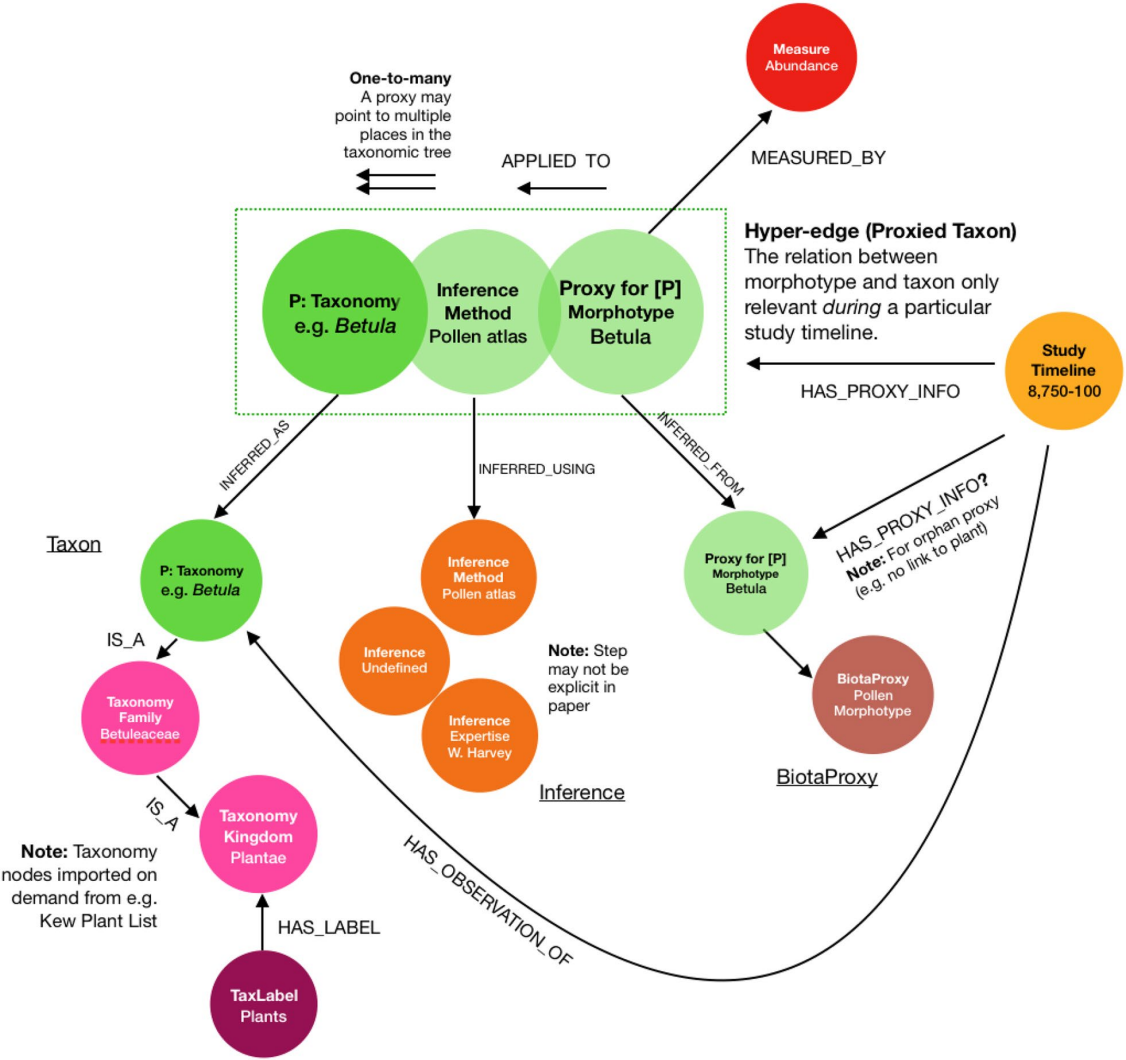
Graph representation of Arctic biodiversity (the Population P for the PECO used within this systematic map protocol). A study timeline may contain information about biodiversity through **a** a biotic proxy inferred as a botanical or other taxon (e.g., pollen morphotype identified as a genus), or **b** a biotic proxy not linked to a taxon (e.g., pollen morphotype only). The combination of three factors—proxy, inference method, and taxon—unique to a particular study timeline are conceptualised as a ‘proxied taxon’ using a *triple*

Within our research question, environmental proxies are used to connect a dated environmental sample to a component of biodiversity. Where proxies are used, a method of inference is required to resolve the proxy measurements to the identify of a biotic component (e.g., a particular genus, species, or set of species). The method of inference chosen within a particular source’s method may change the resultant taxa; this information is paramount therefore to enable cross-comparison of variability in biodiversity between studies. Within our data coding strategy, we will extract information on the proxy measures encountered (e.g., macro-, or micro-fossil type), the biotic component that it has been identified as (e.g., genera linked to a pollen morphotype), and the method of inference whether implicit or explicit (e.g., a specific reference volume or online database used to identify pollen morphotypes; sedimentary DNA barcoding). We will record if any of the three aspects is not included; for example, if the specific pollen morphotypes are not given but only the genus name. The specific biodiversity measure used will be recorded for each proxy occurrence, as shown in Fig. 6. As a result, the final coded biodiversity metadata will include information on the identity of all individual species (and other biodiversity components) within a study. We do not record the values of individual measurements within the time series (e.g., counts of pollen or other microfossils at each level of a core), but only metadata surrounding the relevant biodiversity indicators within the individual study timeline (e.g., identified species, sediment core depths and/or dates).

We will include a backbone of common nodes that represent biodiversity components (see Fig. 6), which will be used to autocomplete inputting of biodiversity metadata. A backbone approach was chosen to ensure that biota were not represented by duplicate concepts, such as taxonomic synonyms and merging of families since a study’s publication. Checklists will be applied as specified in Sect. 4.3.

To enhance the relevance of the coded metadata to specific stakeholders, we will record whether individual studies include collection or assessment of co-occurring environmental datasets, and how each study relates environmental variability to variability in the biodiversity outcomes. The coding method makes the role of narrative versus statistical links to environmental factors distinct. For example, it is common in palaeoecology for multi-proxy datasets to be interpreted within narrative rather than formal statistical tests; in these cases, we will record the nature of the associated datasets and that the link was narrative.

### Study mapping and presentation

A narrative synthesis will be included in the systematic map manuscript. We will include the following descriptive statistics: the number of studies in the initial searches per database that were then excluded at each screening level; included individual biota subset by taxonomic group, rank, and taxonomic label; counts of study designs by ‘proxy method’ used; and proportion of individual biodiversity records by biodiversity measure. We will include statistics on drift between author-given timelines and synthesised timelines based on recalibrated dates.

We will include tables that show evidence gaps identified in space and time. To capture evidence gaps within the spatial–temporal evidence base, we will conduct additional statistical analysis using a polar stereographic projection (EPSG 3413). We will assess the degree to which evidence points are spatially clustered or dispersed by computing spatial autocorrelation using the Global Moran's I statistic (using an inverse distance spatial relationship over Euclidean distance). To identify spatial hotspots, we will use cluster analysis using Getis-Ord Gi^*^ statistic [75]. To identify spatial gaps, we will intersect included site coordinates with three key spatial polygon layers from the Circumpolar Arctic Vegetation Map [62] representing contemporary environmental variability: (1) bioclimatic subzones with the addition of the Oro-Arctic designation [76]; (2) floristic sectors; and (3) subzone x floristic sector. For each intersection, we will identify gaps as polygons with (i) zero and (ii) less than five individual sites; gaps will be stated within tables in the main text, with the full data given in supplementary material.

We will include two key figures: a geographic map of evidence in the Oro-Arctic region; and a graph showing the temporal dimensionality of the evidence. The full-page geographical map will be displayed in an Arctic projection (EPSG 3413) and will show pie charts representing clustering of evidence points. Each pie chart will be proportional to the count of distinct taxa found within the clustered sites, with colour-coded wedges indicating taxonomic group. To show the temporal dimensionality of evidence, we will bin the evidence base into 1000-year (centennial-scale) intervals between 11,650yBP and 2000yBP (aligned with each 1000yBP boundary), and in 500-year intervals from 2000yBP to 0yBP. We will identify temporal evidence gaps by identifying periods for which there is zero overlapping data; this process will be conducted for the whole evidence base, and by the same polygon layers used for the spatial analysis. Temporal evidence gaps will be shown within a unified figure of evidence coverage versus time, colour-coded to the spatial designations used. Temporal gap analysis will be conducted on recalibrated dates rather than author-given dates.

Two key outputs will be created from the systematic map: a graph database and a geo-temporal map of the Arctic. First, the database will be a direct output of the data coding process and will be appended to the manuscript as supplementary data. The graph database will be fronted by a website through which the structure of the graph may be explored. Second, the geo-temporal map will be presented within the *Thalloo* mapping framework [77]; a complete package of code and data will be made available in supplementary material such that the interactive map may be reproduced offline. A narrative will be incorporated into the geographical map to guide stakeholders between key identified knowledge gaps and clusters on spatial, temporal, and taxonomic axes.

## Supplementary Information

**Additional file 1: Appendix A.** Wording and format of initial invites to members of each stakeholder group.

**Additional file 2: Appendix B.** Format of the online consultation used within the initial consultation period.

**Additional file 3: Appendix C.** Search concepts and search terms, and the derived search strings.

**Additional file 4: Appendix D.** ‘Test list’ of sources used for search string calibration and development.

**Additional file 5.** ROSES Checklist.

## Data Availability

The data coding software used will be available alongside the final systematic map. *Cottongrass* multi-lingual configuration files for stakeholder consultation are included in supplementary files.
