# Supplementary material for: What evidence exists for temporal variability in Arctic terrestrial and freshwater biodiversity throughout the Holocene? A systematic map protocol
Source: Environ Evid. 2022 Apr 4;11:13. doi: 10.1186/s13750-022-00267-x (PMC11378824; doi:10.1186/s13750-022-00267-x)
Supplement: Supplementary file 1 — Additional file 1: Appendix A. Wording and format of initial invites to members of each stakeholder group. [file 13750_2022_267_MOESM1_ESM.docx]

**Appendix A: wording of invites to stakeholder groups**

We used four different email templates depending on whether the email was to a scientific / policy stakeholders or interpretation / land manager stakeholders, each with a template to an individual or organisation. We also distributed two flyers with emails to organisations (English and Russian only). The four templates and two flyers are given below.

Template 1: Scientific / Policy stakeholder (individual)

**Subject:** Consultation: Long-term trends in Arctic biodiversity - help us improve our systematic map

Good {morning},

I am emailing on behalf of the EU-funded [CHARTER research project](http://www.charter-arctic.org/), for which we are conducting a systematic map (a type of scientific review) of the evidence on long-term (centuries to millennia) variability in Arctic biodiversity. We have completed the initial planning phase of the review and are now contacting people with interest in the subject area to help us improve our review protocol. The protocol is the formal method that will be used to find and interpret evidence.

We would appreciate if you could pass on this email to {organisation} members or others who you think would be interested.

The aim of our systematic map is to provide an as unbiased way as possible for gathering all information on long-term variability in Arctic biodiversity. The Arctic is undergoing rapid environmental change with a multitude of potential consequences for regional biodiversity. To put these changes into context and to make better predictions of future change, we need to know how Arctic biodiversity has varied in the past. A systematic map is often considered the gold standard for collating such information (you can find out more about systematic maps in additional links at the bottom of this email).

Your knowledge, interest and experiences with the subject matter are important to us. We are now running a brief online consultation to find out:

- Whether you would be interested in the outcomes of our research?
- Whether our review protocol could be improved?
- Whether you have any relevant knowledge (e.g., literature suggestions / own experiences) that could be included in the review process?

**The web consultation can be found here:**[**https://biodiversity.charter-arctic.org**](https://biodiversity.charter-arctic.org)

The consultation is available in multiple languages and takes about 20-30 min to complete. The deadline for the last submission is the **{date}**. The consultation can be filled in anonymously, but you can register your details if you would like to have your contributions acknowledged, would like to collaborate with us, or would like to be informed about the progress and outcomes of the review. You can also contact us by email ({EMAIL}) to discuss over email or setup an online meeting / phone call if you prefer.

We thank you in advance for your feedback and please do not hesitate to contact us should you have any questions.

**{FROM} (lead reviewer) and the CHARTER palaeoecology review team**

***Explainers about ‘systematic reviews’ that may be of interest***

*Quick introduction to systematic reviews (2 min video, medical sciences):*

- [*https://www.youtube.com/watch?v=IDiKpJu_hFI&t=43s*](https://www.youtube.com/watch?v=IDiKpJu_hFI&t=43s)

*More depth:*

- *Interview with Neal Haddaway on systematic maps and reviews in environmental science:*[*https://www.sei.org/featured/what-does-the-science-say-qa/*](https://www.sei.org/featured/what-does-the-science-say-qa/)
- *6 min video of above interview:*[*https://youtu.be/HIiHDHG4AgY*](https://youtu.be/HIiHDHG4AgY)

*Academic reading:*

- *Information for authors – Collaboration for Environmental Evidence:*[*https://environmentalevidence.org/information-for-authors/aims-and-scope/*](https://environmentalevidence.org/information-for-authors/aims-and-scope/)
- *James et al. 2016. A methodology for systematic mapping in environmental sciences.*[*https://doi.org/10.1186/s13750-016-0059-6h*](https://doi.org/10.1186/s13750-016-0059-6h)

Template 2: Scientific / Policy stakeholder (organisation)

**Subject:** Consultation: Long-term trends in Arctic biodiversity - help us improve our systematic map

Good {morning},

I am emailing on behalf of the EU-funded [CHARTER research project](http://www.charter-arctic.org/), for which we are conducting a systematic map (a type of scientific review) of the evidence on long-term (centuries to millennia) variability in Arctic biodiversity. We have completed the initial planning phase of the review and are now contacting people with interest in the subject area to help us improve our review protocol. The protocol is the formal method that will be used to find and interpret evidence.

We would appreciate if you could pass on this email to {organisation} members or others who you think would be interested.

The aim of our systematic map is to provide an as unbiased way as possible for gathering all information on long-term variability in Arctic biodiversity. The Arctic is undergoing rapid environmental change with a multitude of potential consequences for regional biodiversity. To put these changes into context and to make better predictions of future change, we need to know how Arctic biodiversity has varied in the past. A systematic map is often considered the gold standard for collating such information (you can find out more about systematic maps in additional links at the bottom of this email).

The knowledge, interest and experiences of your organisation and your members are important to us. We are now running a brief online consultation to find out:

- Whether your organisation or members would be interested in the outcomes of our research?
- Whether your organisation or members think our review protocol could be improved?
- Whether your organisation or members have any relevant knowledge (e.g., literature suggestions / own experiences) that could be included in the review process?

**The web consultation can be found here:**[**https://biodiversity.charter-arctic.org**](https://biodiversity.charter-arctic.org)

The consultation is available in multiple languages and takes about 20-30 min to complete. The deadline for the last submission is the **{date}**. The consultation can be filled in anonymously, but you can register your details if you would like to have your contributions acknowledged, would like to collaborate with us, or would like to be informed about the progress and outcomes of the review. You can also contact us by email ({EMAIL}) to discuss over email or setup an online meeting / phone call if you prefer.

We thank you in advance for your feedback and please do not hesitate to contact us should you have any questions.

**{FROM} (lead reviewer) and the CHARTER palaeoecology review team**

***Explainers about ‘systematic reviews’ that may be of interest***

*Quick introduction to systematic reviews (2 min video, medical sciences):*

- [*https://www.youtube.com/watch?v=IDiKpJu_hFI&t=43s*](https://www.youtube.com/watch?v=IDiKpJu_hFI&t=43s)

*More depth:*

- *Interview with Neal Haddaway on systematic maps and reviews in environmental science:*[*https://www.sei.org/featured/what-does-the-science-say-qa/*](https://www.sei.org/featured/what-does-the-science-say-qa/)
- *6 min video of above interview:*[*https://youtu.be/HIiHDHG4AgY*](https://youtu.be/HIiHDHG4AgY)

*Academic reading:*

- *Information for authors – Collaboration for Environmental Evidence:*[*https://environmentalevidence.org/information-for-authors/aims-and-scope/*](https://environmentalevidence.org/information-for-authors/aims-and-scope/)
- *James et al. 2016. A methodology for systematic mapping in environmental sciences.*[*https://doi.org/10.1186/s13750-016-0059-6h*](https://doi.org/10.1186/s13750-016-0059-6h)

Template 3: Interpretation / Land use stakeholder (individual)

**Subject:** Interested in long-term trends in Arctic biodiversity? Help us improve our research!

Dear **…**,

I am reaching out on behalf of the [CHARTER research project](http://www.charter-arctic.org/). As part of the project, we are carrying out a form of scientific research called a “systematic map”, for which we are trying to gather as much knowledge as possible on long-term changes in Arctic biodiversity. We have completed the initial planning phase of the research and are now contacting people with interest in the subject to help us improve our research.

The environment is changing rapidly in the Arctic, with many consequences for the variety of things that live in the region, including the plants, animals, and insects. To better understand these consequences and to improve our predictions of future changes, we need to know how this Arctic “biodiversity” has changed in the past. We are therefore trying to gather as much information as possible about these past changes. To do so we use a scientific method called a “systematic map”. You can find out more about such “maps” at the bottom of this email!

The knowledge on the subject within your organisation is of high value to us! We are now running a brief online survey to find out:

- Whether your organisation would be interested in the outcomes of our research?
- Whether your organisation has suggestion to improve our research method?
- Whether your organisation has any relevant knowledge (e.g., reading suggestions / reports / own experiences) that could be included in our research?

The web survey can be found here: INSERT LINK

The survey is available in multiple languages and takes about XX-YY min to complete. The deadline for the last possible submission is the XX of MMMM YYYY. The survey can be filled in anonymously, but you can register your details if you would like to have your contributions acknowledged or would like to be informed about the progress and outcomes of the research. You can also contact us by email (EMAIL) or phone (NUMBER).

You are also very welcome to forward this email to anyone who you think might be interested!

We thank you in advance for sharing your time and knowledge, and please do not hesitate to contact us should you have any questions.

INSERT NAME and the CHARTER WP4 team

*Quick introduction to systematic reviews / maps (2 min video, medical sciences):*

- [*https://www.youtube.com/watch?v=IDiKpJu_hFI&t=43s*](https://www.youtube.com/watch?v=IDiKpJu_hFI&t=43s)

*More detail:*

- *Interview with Neal Haddaway on systematic maps and reviews in environmental science:* [*https://www.sei.org/featured/what-does-the-science-say-qa/*](https://www.sei.org/featured/what-does-the-science-say-qa/)
- *6 min video of above interview:* [*https://youtu.be/HIiHDHG4AgY*](https://youtu.be/HIiHDHG4AgY)

Template 4: Interpretation / Land use stakeholder (organisation)

**Subject:** Interested in long-term trends in Arctic biodiversity? Help us improve our research!

Dear **…**,

I am reaching out on behalf of the [CHARTER research project](http://www.charter-arctic.org/). As part of the project, we are carrying out a form of scientific research called a “systematic map”, for which we are trying to gather as much knowledge as possible on long-term changes in Arctic biodiversity. We have completed the initial planning phase of the research and are now contacting people with interest in the subject to help us improve our research.

The environment is changing rapidly in the Arctic, with many consequences for the variety of things that live in the region, including the plants, animals and insects. To better understand these consequences and to improve our predictions of future changes, we need to know how this Arctic “biodiversity” has changed in the past. We are therefore trying to gather as much information as possible about these past changes. To do so we use a scientific method called a “systematic map”. You can find out more about such “maps” at the bottom of this email!

Your knowledge and interest in the subject area is of high value to us! We are now running a brief online survey to find out:

- Whether you would be interested in the outcomes of our research?
- Whether you think we could improve our research method?
- Whether you have any relevant knowledge (e.g., reading suggestions / own experiences) that could be included in our research?

The web survey can be found here: INSERT LINK

The survey is available in multiple languages and takes about XX-YY min to complete. The deadline for the last possible submission is the XX of MMMM YYYY. The survey can be filled in anonymously, but you can register your details if you would like to have your contributions acknowledged or would like to be informed about the progress and outcomes of the research. You can also contact us by email (EMAIL) or phone (NUMBER).

Please circulate this email with your membership / any individuals within your organisation who you think might be interested!

We thank you in advance for sharing your time and knowledge, and please do not hesitate to contact us should you have any questions.

INSERT NAME and the CHARTER WP4 team

*Quick introduction to systematic reviews / maps (2 min video, medical sciences):*

- [*https://www.youtube.com/watch?v=IDiKpJu_hFI&t=43s*](https://www.youtube.com/watch?v=IDiKpJu_hFI&t=43s)

*More detail:*

- *Interview with Neal Haddaway on systematic maps and reviews in environmental science:* [*https://www.sei.org/featured/what-does-the-science-say-qa/*](https://www.sei.org/featured/what-does-the-science-say-qa/)
- *6 min video of above interview:* [*https://youtu.be/HIiHDHG4AgY*](https://youtu.be/HIiHDHG4AgY)

Template 5: Flyer distributed to interest groups (Russian)

Template 6: Flyer distributed to interest groups (English)
