# Supplementary material for: What evidence exists for temporal variability in Arctic terrestrial and freshwater biodiversity throughout the Holocene? A systematic map protocol
Source: Environ Evid. 2022 Apr 4;11:13. doi: 10.1186/s13750-022-00267-x (PMC11378824; doi:10.1186/s13750-022-00267-x)
Supplement: Supplementary file 2 — Additional file 2: Appendix B. Format of the online consultation used within the initial consultation period. [file 13750_2022_267_MOESM2_ESM.docx]

**Appendix B: online consultation format**

The online consultation is defined in a YAML file and scaffolded into a single page web application by the *Cottongrass* application (https://github.com/AndrewIOM/cottongrass). Our consultation was configured to display questions in ten sections:

- About You
- Our primary research question - scope and definitions
- Search Strategy
- Search Concepts - Population (Arctic Biota)
- Search Concepts - Population Context (Arctic)
- Search Concepts - Exposure (Time)
- Search Concepts - Outcomes (Measure of Biodiversity)
- Search Concepts - Biotic Proxies used for Environmental Reconstruction
- Grey Literature Sources
- Anything else?

The multilingual YAML configuration file is attached separately to this manuscript.
