# Supplementary material for: What evidence exists for temporal variability in Arctic terrestrial and freshwater biodiversity throughout the Holocene? A systematic map protocol
Source: Environ Evid. 2022 Apr 4;11:13. doi: 10.1186/s13750-022-00267-x (PMC11378824; doi:10.1186/s13750-022-00267-x)
Supplement: Supplementary file 3 — Additional file 3: Appendix C. Search concepts and search terms, and the derived search strings. [file 13750_2022_267_MOESM3_ESM.docx]

**Appendix C: search concepts and search strings**

We compiled our search strings in a PECO structure, organised as a set of concepts for each of the Population, Population context, Exposure, and Outcomes. We refined our concepts and their associated terms within the initial stakeholder engagement phase; changes made following stakeholder input are highlighted in green. Concepts for each component are shown in the following tables.

Table 3 ‘Population’ concepts

| **Concept** | **Search string fragment** |
| --- | --- |
| Plants (general) | ("plant?" OR "flora" OR "vegetation" OR "Plantae" OR "vegetational" OR "pal$eovegetation" OR "floristic") OR |
| Non-vascular plants | ("bryophyte" OR "Bryophyta" OR "liverwort?" OR "moss" OR "mosses") OR |
| Vascular plants | ("shrub?" OR "woody plant" OR "vascular plant" OR "fern?" OR "pteridophyt?" OR "spermatopht?" OR "xerophyte" OR "tree species" OR "coniferous" OR "conifer" OR "deciduous" OR "herb" OR “forb?”) OR |
| Boreal forest / treeline species, shrub species | ("forest species" OR "treeline species" OR "tree-line species" OR "larch" OR "Larix" OR "spruce" OR "picea" OR "western hemlock" OR "Tsuga" OR "Sitka" OR "alnus" OR “alder” OR "cottonwood" OR "Populus" OR “poplar” OR "Pinus" OR “pine” OR "Betula" OR “birch” OR "willow" OR “salix”) OR |
| Fungi | ("lichen?" OR "sporomiella" OR "dung fungal spore" OR “fungi” OR “fungal”) OR |
| Diatoms | (“diatom” OR “Bacillariophyceae”) OR |
| Molluscs | ("Mollusca" OR "mollusk" OR "mollusc" OR "Gastropoda" OR "gastropod" OR "Bivalvia" OR "bivalve") OR |
| Tetrapods | ("mammal" OR "mammalian" OR "mammoth" OR "reindeer" OR "caribou" OR "Rangifer tarandus" OR "polar bear" OR "Mammuthus primigenius" OR "steppe bison" OR "Bison priscus" OR "muskox" OR "Ovibos moschatus" OR "moose" OR "Alcos alces" OR "horse" OR "Equus caballus" OR "wolf" OR "wolves" OR "Canis lupus" OR "dog" OR "squirrel" OR "rodent" OR "Rodentia" OR "Urocitellus" OR "arctic fox" OR "vulpes lagopus" OR "alopex lagopus" OR “reptile” OR “bird?” OR “Aves”) OR |
| Ostracods | ("ostracod" OR "ostracode" OR “ostracoda”) OR |
| Beetles | (“coleoptera” OR “beetle”) OR |
| Chironomids | (“chironomid” OR “Chironomidae”) OR |
| Invertebrates – general concepts | ("insect" OR "macroinvertebrate" OR "macro-invertebrate") OR |
| Aquatic – general concepts | ("amphibian" OR "Amphibia" OR "aquatic animal" OR "algae" OR "algal") OR |
| Vertebrates – general concepts | ("animal" OR "fauna" OR "faunal" OR "megafauna" OR "megaherbivore" OR "vertebrata" OR "vertebrate") |

Table 4 ‘Population Context’ concepts

| **Concept** | **Search string fragment** |
| --- | --- |
| Labels for the Arctic | ("arctic" OR "high-latitude" OR "North Polar region" OR "subarctic" OR "northern environ*") OR |
| Arctic countries | ("Canada" OR “Canadian” OR "Russia" OR "Russian Federation" OR "USSR" OR "RSFSR" OR "Russian Federation" OR “Russian” OR "Norway" OR “Norwegian” OR "Finland" OR “Finnish” OR "Sweden" OR “Iceland” OR “Icelandic” OR “Greenland” OR “Greenlandic” OR “Faroe”) OR |
| Arctic biomes | ("tundra" OR "permafrost" OR "mammoth steppe") OR |
| Canada / USA | ("Yukon Territory" OR "Northwest Territories" OR "Nunavut" OR "Baffin Island" OR "Belcher Islands" OR "Ellesmere Island" OR "Southampton Island" OR "Mackenzie River" OR "Great Slave Lake" OR "Great Bear Lake" OR "Yukon River" OR "Alaska" OR "Teshekpuk Lake" OR "Queen Elizabeth Islands" OR "Victoria Island" OR "Banks Island" OR "Wrangel Island" OR "Seward peninsula" OR "Hudson Bay") OR |
| Russia | ("Siberia" OR “Siberian” OR "Lake Baikal" OR "Lena River" OR "New Siberian Islands" OR "Severnaya Zemlya" OR "Franz Josef Land" OR "Novaya Zemlya" OR "Chukotka Autonomous Okrug" OR "Kamchatka Krai" OR "Magadan Oblast" OR "Murmansk Oblast" OR "Sakha" OR "Arkhangelsk Oblast" OR "Irkutsk Oblast" OR "Khabarovsk Krai" OR "Komi Republic" OR "Krasnoyarsk Krai" OR "Republic of Karelia" OR "Sakhalin Oblast" OR "Tuva" OR "Tyumen Oblast" OR "Polar Urals" OR "Yamalia") OR |
| Europe (Finland / Sweden / Norway) | ("Svalbard" OR "Spitsbergen" OR "Bjørnøya" OR "Jan Mayen" OR "Finnmark" OR "Troms" OR "Lapland" or "Lappi" OR "Grímsey" OR "Lappland" OR "Norrbotten" OR OR “Västerbotten” OR "North West Europe" OR "Northwest Europe" OR "north* Europe" OR "Scandes" OR "Kola Peninsula") OR |
| Beringia | ("beringia" OR "beringian") OR |
| Global datasets | ("global data*") |

Table 5 'Exposure' concepts

| **Concept** | **New search string** |
| --- | --- |
| Reconstructions | ("reconstruct" OR “reconstruction” OR “reconstructed”) OR |
| Palaeoecology etc. | ("pal$eo*" OR "micropal$entol*") OR |
| Archeology | ("arch$eolog*" OR "artefact") OR |
| Historical | ("history" OR "historic site" OR "historical record") OR |
| Wood ring dating | ("dendrochron*" OR "dendroclim*" OR "dendroeco*" OR "growth ring" OR "tree ring" OR “wood ring”) OR |
| Radiocarbon dating | ("radiocarbon" OR "radio-carbon" OR "AMS" OR "accelerator mass spectrometry") OR |
| Age determination | ("age determination" OR "years before present" OR "yr BP" OR "calibration of age" OR "age model" OR "age-depth model") OR |
| Old dates | ("1?,??? 14C yr B$P" OR "1? ??? 14C Yr B$P" OR "1?,??? yr B$P" OR "1? ??? yr B$P" OR "1?,??? cal yr B$P" OR "1? ??? cal yr B?P" OR "?,??? 14C yr B?P" OR "? ??? 14C Yr B?P" OR "?,??? yr B?P" OR "? ??? yr B?P" OR "?,??? cal yr B?P" OR "? ??? cal yr B?P" OR "pre-1???" OR "pre-20th century" OR "1?th century" OR "last ?,??? years" OR "last 1?,??? years" OR "14C dates" OR "*th century" OR "? Cal Ka" OR "1? Cal Ka" OR “one century” OR “preindustrial” OR “pre-industrial”) OR |
| Fossils | ("fossil" OR "sub-fossil" OR "macrofossil" OR "megafossil" OR "microfossil" OR "subfossil") OR |
| Palynology (method) | ("palynolog*") OR |
| Archeology time periods | ("prehistoric" OR "prehistory" OR "Iron Age" OR “Bronze Age” OR “Neolithic” OR “Mesolithic” OR “Late Upper Pal$eolithic” OR "BC") OR |
| Time periods (Holocene etc.) | ("Holocene" OR "MidHolocene" OR "Mid-Holocene" OR "late Pleistocene" OR "end of the Pleistocene" OR "Late Quaternary" OR "Little Ice Age" OR "Medieval Climate Anomaly" OR "Younger Dryas" OR "MIS 1" OR "marine isotope stage 1") OR |
| Temporal variability | ("centennial" OR "centuries" OR "millenial" OR "millenia" OR "submillenial" OR "chronolog*" OR "temporal trend") OR |
| Time periods: chronozones | ("preboreal chronozone" OR "boreal chronozone" OR "atlantic chronozone" OR "subboreal chronozone") OR |
| Sediment cores | ("sediment core" OR "sedimentary sequence" OR "lake sediment" OR "multiproxy sediment* analysis" OR "transfer function" OR “peat core” OR “peat bog core” OR “peat monolith”) OR |
| aDNA | ("ancient DNA" OR "aDNA" OR "sedDNA" OR "sedimentary DNA") OR |
| Glaciers | ("postglacial" OR "post-glacial" OR "deglaciation" OR "deglacial") OR |
| Persistence | ("last appearance" OR "refugia") OR |
| Proxies | ("multiproxy" OR "proxy record" OR "proxy archive" OR "proxy" OR "proxy study" OR "proxy studies") OR |
| Research Fields | ("long-term ecolog*" OR "long-term record") OR |

Table 6 'Outcomes' concepts

| **Concept** | **New search string** |
| --- | --- |
| Composition | ("composition" OR "assemblage" OR "pal$eocommunit*" OR "compositional shift" OR "species dominance") OR |
| Diversity | ("diversity" OR “evenness”) OR |
| Richness | ("richness" OR "number of species") OR |
| Biodiversity | ("biodiversity") OR |
| Presence / Absence | ("presence" OR "present" OR "absence" OR "absent" OR "occurrence" OR "occurred") OR |
| Persistence | ("stability" OR "persistence" OR "persisted" OR "disappeared" OR "extinction") OR |
| Abundance | ("abundance") OR |
| Succession | ("succession" OR "vegetation development" OR "plant cover" OR "zonation pattern") OR |
| Distribution | ("distribution" OR "distributed" OR “distributional”) OR |
| Colonisation / Establishment | ("coloni?ed" OR "coloni?ation" OR "established" OR "spread to") OR |
| Ecotone / Zonation / Treeline | ("northern limit" OR "northern treeline" OR "ecotone" OR “habitat diversity”) |

In addition to the Population, Population Context, Exposure, and Outcomes, calibration of the search string against the test list indicated that many sources that contained information that may be used to assess the effect of centennial-scale temporal dynamics on Arctic biodiversity, but for which the authors: (a) used the information to reconstruct climate or other environmental properties rather than biodiversity outcomes; and / or (b) did not establish the links between fossil remains and the originating taxon. We assessed the appropriateness of including additional concepts to capture these studies through additional test searches in Scopus; these indicated that adding the additional concepts did not substantially increase the number of false negatives returned but did capture the identified articles in the test list. The concepts added to the Population and Outcomes are shown in the two tables below.

Table 7 Additional concepts for 'Population' for cases where methods did not establish taxonomic identities, or biota missing from title and abstract.

| **Concept** | **New search string** |
| --- | --- |
| Pollen morphotypes | ("pollen" OR "palynology" OR "macrofossil" OR "pal?eobotanical" OR "leaf wax" OR "palynomorph") OR |
| Individual biotic proxies | ("organic biomarker") OR |
| Dendrochronology – plant parts | ("ring width" OR "wood ring" OR "tree ring" OR "stem sample" OR "driftwood") OR |
| General terms | ("terrestrial proxies" OR "terrestrial proxy") |

Table 8 Additional concepts for ‘Outcome’ for cases where methods did not reconstruct biodiversity but rather used a biotic proxy to reconstruct other environmental factors.

| Concept | New search string |
| --- | --- |
| Temperature | ("warming" OR "cooling" OR "temperature" OR "temperatures" OR "warm period" OR "cold period" OR "summer warmth") OR |
| Precipitation and Hydrology | ("hydroclimate" OR "precipitation") OR |
| Past climates | ("pal?eoclimat*" OR "past climate" OR "climate of the past") OR |
| Lake levels | ("lake level") OR |
| Vegetation productivity / Individual performance | ("vegetation productivity" OR "plant productivity" OR "radial growth" OR "annual growth" OR "growth rate" OR "fuel load") OR |
| Climate (general) | ("climate variable" OR "climate interpretation" OR "proxy climate record") OR |
| Snow patterns | ("snow cover" OR "snow extent") OR |
| Carbon storage | ("organic carbon release" OR "permafrost carbon" OR "soil carbon release") OR |
| Sea ice extent | ("sea ice extent" OR "relative sea level" OR "sea level rise") OR |
| Human interactions with animals and plants | ("hunting" OR "husbandry" OR "domestication" OR "construction activity" OR "settlement history") |

# Search strings – compiled

The search concepts and search terms were compiled into database-specific search strings, which are given below.

Scopus

TITLE-ABS-KEY(

(

("biodiversity") OR ("diversity" OR "evenness") OR ("richness" OR "number of species") OR ("composition" OR "assemblage" OR "pal*eocommunit*" OR "compositional shift" OR "species dominance") OR ("presence" OR "present" OR "absence" OR "absent" OR "occurrence" OR "occurred") OR ("stability" OR "persistence" OR "persisted" OR "disappeared" OR "extinction") OR ("abundance") OR ("succession" OR "vegetation development" OR "plant cover" OR "zonation pattern") OR ("distribution" OR "distributed" OR "distributional") OR ("coloni?ed" OR "coloni?ation" OR "established" OR "spread to") OR ("northern limit" OR "northern treeline" OR "ecotone" OR "habitat diversity") OR ("warming" OR "cooling" OR "temperature" OR "temperatures" OR "warm period" OR "cold period" OR "summer warmth") OR ("hydroclimate" OR "precipitation") OR ("pal*eoclimat*" OR "past climate" OR "climate of the past") OR ("lake level") OR ("vegetation productivity" OR "plant productivity" OR "radial growth" OR "annual growth" OR "growth rate" OR "fuel load") OR ("climate variable" OR "climate interpretation" OR "proxy climate record") OR ("snow cover" OR "snow extent") OR ("organic carbon release" OR "permafrost carbon" OR "soil carbon release") OR ("sea ice extent" OR "relative sea level" OR "sea level rise") OR ("hunting" OR "husbandry" OR "domestication" OR "construction activity" OR "settlement history")

)

AND

(

("arctic" OR "high-latitude" OR "oro-arctic" OR "North Polar region" OR "subarctic" OR "northern environ*") OR ("Canada" OR "Canadian" OR "Russia" OR "Russian Federation" OR "USSR" OR "RSFSR" OR "Russian Federation" OR "Russian" OR "Norway" OR "Norwegian" OR "Finland" OR "Finnish" OR "Sweden" OR "Iceland" OR "Icelandic" OR "Greenland" OR "Greenlandic" OR "Faroe") OR ("tundra" OR "permafrost" OR "mammoth steppe") OR ("Yukon Territory" OR "Northwest Territories" OR "Nunavut" OR "Baffin Island" OR "Belcher Islands" OR "Ellesmere Island" OR "Southampton Island" OR "Mackenzie River" OR "Great Slave Lake" OR "Great Bear Lake" OR "Yukon River" OR "Alaska" OR "Teshekpuk Lake" OR "Queen Elizabeth Islands" OR "Victoria Island" OR "Banks Island" OR "Wrangel Island" OR "Seward peninsula" OR "Hudson Bay") OR ("Siberia" OR "Siberian" OR "Lake Baikal" OR "Lena River" OR "New Siberian Islands" OR "Severnaya Zemlya" OR "Franz Josef Land" OR "Novaya Zemlya" OR "Chukotka Autonomous Okrug" OR "Kamchatka Krai" OR "Magadan Oblast" OR "Murmansk Oblast" OR "Sakha" OR "Arkhangelsk Oblast" OR "Irkutsk Oblast" OR "Khabarovsk Krai" OR "Komi Republic" OR "Krasnoyarsk Krai" OR "Republic of Karelia" OR "Sakhalin Oblast" OR "Tuva" OR "Tyumen Oblast" OR "Polar Urals" OR "Yamalia") OR ("Svalbard" OR "Spitsbergen" OR "Bjørnøya" OR "Jan Mayen" OR "Finnmark" OR "Troms" OR "Lapland" or "Lappi" OR "Grímsey" OR "Lappland" OR "Norrbotten" OR "Västerbotten" OR "North West Europe" OR "Northwest Europe" OR "north* Europe" OR "Scandes" OR "Kola Peninsula") OR ("beringia" OR "beringian") OR ("global data*")

)

AND

(

("reconstruct" OR "reconstruction" OR "reconstructed") OR ("pal*eo*" OR "micropal*entol*") OR ("arch*eolog*" OR "artefact") OR ("history" OR "historic site" OR "historical record") OR ("dendrochron*" OR "dendroclim*" OR "dendroeco*" OR "growth ring" OR "tree ring" OR "wood ring") OR ("radiocarbon" OR "radio-carbon" OR "AMS" OR "accelerator mass spectrometry") OR ("age determination" OR "years before present" OR "yr BP" OR "calibration of age" OR "age model" OR "age-depth model") OR ("1?,??? 14C yr B*P" OR "1? ??? 14C Yr B*P" OR "1?,??? yr B*P" OR "1? ??? yr B*P" OR "1?,??? cal yr B*P" OR "1? ??? cal yr B*P" OR "pre-1???" OR "pre-20th century" OR "1?th century" OR "last ?,??? years" OR "last 1?,??? years" OR "14C dates" OR "1? Cal Ka" OR "one century" OR "pre-industrial" OR "preindustrial") OR ("fossil" OR "sub-fossil" OR "macrofossil" OR "megafossil" OR "microfossil" OR "subfossil") OR ("palynolog*") OR ("prehistoric" OR "prehistory" OR "Iron Age" OR "Bronze Age" OR "Neolithic" OR "Mesolithic" OR "Late Upper Pal*eolithic" OR "BC") OR ("Holocene" OR "MidHolocene" OR "Mid-Holocene" OR "late Pleistocene" OR "end of the Pleistocene" OR "Late Quaternary" OR "Little Ice Age" OR "Medieval Climate Anomoly" OR "Younger Dryas" OR "MIS 1" OR "marine isotope stage 1") OR ("centennial" OR "centuries" OR "millenial" OR "millenia" OR "submillenial" OR "chronolog*" OR "temporal trend") OR ("preboreal chronozone" OR "boreal chronozone" OR "atlantic chronozone" OR "subboreal chronozone") OR ("sediment core" OR "sedimentary sequence" OR "lake sediment" OR "transfer function" OR "peat core" OR "peat bog core" OR "peat monolith") OR ("ancient DNA" OR "aDNA" OR "sedDNA" OR "sedimentary DNA") OR ("postglacial" OR "post-glacial" OR "deglaciation" OR "deglacial") OR ("last appearance date" OR "refugia") OR ("multiproxy" OR "proxy record" OR "proxy archive" OR "proxy" OR "proxy study" OR "proxy studies") OR ("long-term ecolog*" OR "long-term record")

)

AND

(

("plant?" OR "flora" OR "vegetation" OR "Plantae" OR "vegetational" OR "pal?eovegetation" OR "floristic") OR ("bryophyte" OR "Bryophyta" OR "liverwort?" OR "moss" OR "mosses") OR ("shrub?" OR "woody plant" OR "vascular plant" OR "fern?" OR "pteridophyt?" OR "spermatopht?" OR "xerophyte" OR "tree species" OR "coniferous" OR "conifer" OR "deciduous" OR "herb" OR "forb?") OR ("forest species" OR "treeline species" OR "tree-line species" OR "larch" OR "Larix" OR "spruce" OR "picea" OR "western hemlock" OR "Tsuga" OR "Sitka" OR "alnus" OR "alder" OR "cottonwood" OR "Populus" OR "poplar" OR "Pinus" OR "pine" OR "Betula" OR "birch" OR "willow" OR "salix") OR ("lichen?" OR "sporomiella" OR "dung fungal spore" OR "fungi" OR "fungal") OR ("diatom" OR "Bacillariophyceae") OR ("Mollusca" OR "mollusk" OR "mollusc" OR "Gastropoda" OR "gastropod" OR "Bivalvia" OR "bivalve") OR ("mammal" OR "mammalian" OR "mammoth" OR "reindeer" OR "caribou" OR "Rangifer tarandus" OR "polar bear" OR "Mammuthus primigenius" OR "steppe bison" OR "Bison priscus" OR "muskox" OR "Ovibos moschatus" OR "moose" OR "Alcos alces" OR "horse" OR "Equus caballus" OR "wolf" OR "wolves" OR "Canis lupus" OR "dog" OR "squirrel" OR "rodent" OR "Rodentia" OR "Urocitellus" OR "arctic fox" OR "vulpes lagopus" OR "alopex lagopus" OR "reptile" OR "bird?" OR "Aves") OR ("ostracod" OR "ostracode" OR "ostracoda") OR ("coleoptera" OR "beetle") OR ("chironomid" OR "Chironomidae") OR ("insect" OR "macroinvertebrate" OR "macro-invertebrate") OR ("amphibian" OR "Amphibia" OR "aquatic animal" OR "algae" OR "algal") OR ("animal" OR "fauna" OR "faunal" OR "megafauna" OR "megaherbivore" OR "vertebrata" OR "vertebrate") OR ("pollen" OR "palynology" OR "macrofossil" OR "pal*eobotanical" OR "leaf wax" OR "palynomorph") OR ("organic biomarker") OR ("ring width" OR "wood ring" OR "tree ring" OR "stem sample" OR "driftwood") OR ("terrestrial proxies" OR "terrestrial proxy")

)

)

BIOSIS via Web of Science

(TI=(

("biodiversity") OR ("diversity" OR "evenness") OR ("richness" OR "number of species") OR ("composition" OR "assemblage" OR "pal$eocommunit*" OR "compositional shift" OR "species dominance") OR ("presence" OR "present" OR "absence" OR "absent" OR "occurrence" OR "occurred") OR ("stability" OR "persistence" OR "persisted" OR "disappeared" OR "extinction") OR ("abundance") OR ("succession" OR "vegetation development" OR "plant cover" OR "zonation pattern") OR ("distribution" OR "distributed" OR "distributional") OR ("coloni?ed" OR "coloni?ation" OR "established" OR "spread to") OR ("northern limit" OR "northern treeline" OR "ecotone" OR "habitat diversity") OR ("warming" OR "cooling" OR "temperature" OR "temperatures" OR "warm period" OR "cold period" OR "summer warmth") OR ("hydroclimate" OR "precipitation") OR ("pal$eoclimat*" OR "past climate" OR "climate of the past") OR ("lake level") OR ("vegetation productivity" OR "plant productivity" OR "radial growth" OR "annual growth" OR "growth rate" OR "fuel load") OR ("climate variable" OR "climate interpretation" OR "proxy climate record") OR ("snow cover" OR "snow extent") OR ("organic carbon release" OR "permafrost carbon" OR "soil carbon release") OR ("sea ice extent" OR "relative sea level" OR "sea level rise") OR ("hunting" OR "husbandry" OR "domestication" OR "construction activity" OR "settlement history")

) OR TS=(

("biodiversity") OR ("diversity" OR "evenness") OR ("richness" OR "number of species") OR ("composition" OR "assemblage" OR "pal$eocommunit*" OR "compositional shift" OR "species dominance") OR ("presence" OR "present" OR "absence" OR "absent" OR "occurrence" OR "occurred") OR ("stability" OR "persistence" OR "persisted" OR "disappeared" OR "extinction") OR ("abundance") OR ("succession" OR "vegetation development" OR "plant cover" OR "zonation pattern") OR ("distribution" OR "distributed" OR "distributional") OR ("coloni?ed" OR "coloni?ation" OR "established" OR "spread to") OR ("northern limit" OR "northern treeline" OR "ecotone" OR "habitat diversity") OR ("warming" OR "cooling" OR "temperature" OR "temperatures" OR "warm period" OR "cold period" OR "summer warmth") OR ("hydroclimate" OR "precipitation") OR ("pal$eoclimat*" OR "past climate" OR "climate of the past") OR ("lake level") OR ("vegetation productivity" OR "plant productivity" OR "radial growth" OR "annual growth" OR "growth rate" OR "fuel load") OR ("climate variable" OR "climate interpretation" OR "proxy climate record") OR ("snow cover" OR "snow extent") OR ("organic carbon release" OR "permafrost carbon" OR "soil carbon release") OR ("sea ice extent" OR "relative sea level" OR "sea level rise") OR ("hunting" OR "husbandry" OR "domestication" OR "construction activity" OR "settlement history")

) OR CC=(

("biodiversity") OR ("diversity" OR "evenness") OR ("richness" OR "number of species") OR ("composition" OR "assemblage" OR "pal$eocommunit*" OR "compositional shift" OR "species dominance") OR ("presence" OR "present" OR "absence" OR "absent" OR "occurrence" OR "occurred") OR ("stability" OR "persistence" OR "persisted" OR "disappeared" OR "extinction") OR ("abundance") OR ("succession" OR "vegetation development" OR "plant cover" OR "zonation pattern") OR ("distribution" OR "distributed" OR "distributional") OR ("coloni?ed" OR "coloni?ation" OR "established" OR "spread to") OR ("northern limit" OR "northern treeline" OR "ecotone" OR "habitat diversity") OR ("warming" OR "cooling" OR "temperature" OR "temperatures" OR "warm period" OR "cold period" OR "summer warmth") OR ("hydroclimate" OR "precipitation") OR ("pal$eoclimat*" OR "past climate" OR "climate of the past") OR ("lake level") OR ("vegetation productivity" OR "plant productivity" OR "radial growth" OR "annual growth" OR "growth rate" OR "fuel load") OR ("climate variable" OR "climate interpretation" OR "proxy climate record") OR ("snow cover" OR "snow extent") OR ("organic carbon release" OR "permafrost carbon" OR "soil carbon release") OR ("sea ice extent" OR "relative sea level" OR "sea level rise") OR ("hunting" OR "husbandry" OR "domestication" OR "construction activity" OR "settlement history")

) OR AB=(

("biodiversity") OR ("diversity" OR "evenness") OR ("richness" OR "number of species") OR ("composition" OR "assemblage" OR "pal$eocommunit*" OR "compositional shift" OR "species dominance") OR ("presence" OR "present" OR "absence" OR "absent" OR "occurrence" OR "occurred") OR ("stability" OR "persistence" OR "persisted" OR "disappeared" OR "extinction") OR ("abundance") OR ("succession" OR "vegetation development" OR "plant cover" OR "zonation pattern") OR ("distribution" OR "distributed" OR "distributional") OR ("coloni?ed" OR "coloni?ation" OR "established" OR "spread to") OR ("northern limit" OR "northern treeline" OR "ecotone" OR "habitat diversity") OR ("warming" OR "cooling" OR "temperature" OR "temperatures" OR "warm period" OR "cold period" OR "summer warmth") OR ("hydroclimate" OR "precipitation") OR ("pal$eoclimat*" OR "past climate" OR "climate of the past") OR ("lake level") OR ("vegetation productivity" OR "plant productivity" OR "radial growth" OR "annual growth" OR "growth rate" OR "fuel load") OR ("climate variable" OR "climate interpretation" OR "proxy climate record") OR ("snow cover" OR "snow extent") OR ("organic carbon release" OR "permafrost carbon" OR "soil carbon release") OR ("sea ice extent" OR "relative sea level" OR "sea level rise") OR ("hunting" OR "husbandry" OR "domestication" OR "construction activity" OR "settlement history")

) OR DE=(

("biodiversity") OR ("diversity" OR "evenness") OR ("richness" OR "number of species") OR ("composition" OR "assemblage" OR "pal$eocommunit*" OR "compositional shift" OR "species dominance") OR ("presence" OR "present" OR "absence" OR "absent" OR "occurrence" OR "occurred") OR ("stability" OR "persistence" OR "persisted" OR "disappeared" OR "extinction") OR ("abundance") OR ("succession" OR "vegetation development" OR "plant cover" OR "zonation pattern") OR ("distribution" OR "distributed" OR "distributional") OR ("coloni?ed" OR "coloni?ation" OR "established" OR "spread to") OR ("northern limit" OR "northern treeline" OR "ecotone" OR "habitat diversity") OR ("warming" OR "cooling" OR "temperature" OR "temperatures" OR "warm period" OR "cold period" OR "summer warmth") OR ("hydroclimate" OR "precipitation") OR ("pal$eoclimat*" OR "past climate" OR "climate of the past") OR ("lake level") OR ("vegetation productivity" OR "plant productivity" OR "radial growth" OR "annual growth" OR "growth rate" OR "fuel load") OR ("climate variable" OR "climate interpretation" OR "proxy climate record") OR ("snow cover" OR "snow extent") OR ("organic carbon release" OR "permafrost carbon" OR "soil carbon release") OR ("sea ice extent" OR "relative sea level" OR "sea level rise") OR ("hunting" OR "husbandry" OR "domestication" OR "construction activity" OR "settlement history")

))

AND

(TI=(

("arctic" OR "high-latitude" OR "oro-arctic" OR "North Polar region" OR "subarctic" OR "northern environ*") OR ("Canada" OR "Canadian" OR "Russia" OR "Russian Federation" OR "USSR" OR "RSFSR" OR "Russian Federation" OR "Russian" OR "Norway" OR "Norwegian" OR "Finland" OR "Finnish" OR "Sweden" OR "Iceland" OR "Icelandic" OR "Greenland" OR "Greenlandic" OR "Faroe") OR ("tundra" OR "permafrost" OR "mammoth steppe") OR ("Yukon Territory" OR "Northwest Territories" OR "Nunavut" OR "Baffin Island" OR "Belcher Islands" OR "Ellesmere Island" OR "Southampton Island" OR "Mackenzie River" OR "Great Slave Lake" OR "Great Bear Lake" OR "Yukon River" OR "Alaska" OR "Teshekpuk Lake" OR "Queen Elizabeth Islands" OR "Victoria Island" OR "Banks Island" OR "Wrangel Island" OR "Seward peninsula" OR "Hudson Bay") OR ("Siberia" OR "Siberian" OR "Lake Baikal" OR "Lena River" OR "New Siberian Islands" OR "Severnaya Zemlya" OR "Franz Josef Land" OR "Novaya Zemlya" OR "Chukotka Autonomous Okrug" OR "Kamchatka Krai" OR "Magadan Oblast" OR "Murmansk Oblast" OR "Sakha" OR "Arkhangelsk Oblast" OR "Irkutsk Oblast" OR "Khabarovsk Krai" OR "Komi Republic" OR "Krasnoyarsk Krai" OR "Republic of Karelia" OR "Sakhalin Oblast" OR "Tuva" OR "Tyumen Oblast" OR "Polar Urals" OR "Yamalia") OR ("Svalbard" OR "Spitsbergen" OR "Bjørnøya" OR "Jan Mayen" OR "Finnmark" OR "Troms" OR "Lapland" or "Lappi" OR "Grímsey" OR "Lappland" OR "Norrbotten" OR "Västerbotten" OR "North West Europe" OR "Northwest Europe" OR "north* Europe" OR "Scandes" OR "Kola Peninsula") OR ("beringia" OR "beringian") OR ("global data*")

) OR TS=(

("arctic" OR "high-latitude" OR "oro-arctic" OR "North Polar region" OR "subarctic" OR "northern environ*") OR ("Canada" OR "Canadian" OR "Russia" OR "Russian Federation" OR "USSR" OR "RSFSR" OR "Russian Federation" OR "Russian" OR "Norway" OR "Norwegian" OR "Finland" OR "Finnish" OR "Sweden" OR "Iceland" OR "Icelandic" OR "Greenland" OR "Greenlandic" OR "Faroe") OR ("tundra" OR "permafrost" OR "mammoth steppe") OR ("Yukon Territory" OR "Northwest Territories" OR "Nunavut" OR "Baffin Island" OR "Belcher Islands" OR "Ellesmere Island" OR "Southampton Island" OR "Mackenzie River" OR "Great Slave Lake" OR "Great Bear Lake" OR "Yukon River" OR "Alaska" OR "Teshekpuk Lake" OR "Queen Elizabeth Islands" OR "Victoria Island" OR "Banks Island" OR "Wrangel Island" OR "Seward peninsula" OR "Hudson Bay") OR ("Siberia" OR "Siberian" OR "Lake Baikal" OR "Lena River" OR "New Siberian Islands" OR "Severnaya Zemlya" OR "Franz Josef Land" OR "Novaya Zemlya" OR "Chukotka Autonomous Okrug" OR "Kamchatka Krai" OR "Magadan Oblast" OR "Murmansk Oblast" OR "Sakha" OR "Arkhangelsk Oblast" OR "Irkutsk Oblast" OR "Khabarovsk Krai" OR "Komi Republic" OR "Krasnoyarsk Krai" OR "Republic of Karelia" OR "Sakhalin Oblast" OR "Tuva" OR "Tyumen Oblast" OR "Polar Urals" OR "Yamalia") OR ("Svalbard" OR "Spitsbergen" OR "Bjørnøya" OR "Jan Mayen" OR "Finnmark" OR "Troms" OR "Lapland" or "Lappi" OR "Grímsey" OR "Lappland" OR "Norrbotten" OR "Västerbotten" OR "North West Europe" OR "Northwest Europe" OR "north* Europe" OR "Scandes" OR "Kola Peninsula") OR ("beringia" OR "beringian") OR ("global data*")

) OR CC=(

("arctic" OR "high-latitude" OR "oro-arctic" OR "North Polar region" OR "subarctic" OR "northern environ*") OR ("Canada" OR "Canadian" OR "Russia" OR "Russian Federation" OR "USSR" OR "RSFSR" OR "Russian Federation" OR "Russian" OR "Norway" OR "Norwegian" OR "Finland" OR "Finnish" OR "Sweden" OR "Iceland" OR "Icelandic" OR "Greenland" OR "Greenlandic" OR "Faroe") OR ("tundra" OR "permafrost" OR "mammoth steppe") OR ("Yukon Territory" OR "Northwest Territories" OR "Nunavut" OR "Baffin Island" OR "Belcher Islands" OR "Ellesmere Island" OR "Southampton Island" OR "Mackenzie River" OR "Great Slave Lake" OR "Great Bear Lake" OR "Yukon River" OR "Alaska" OR "Teshekpuk Lake" OR "Queen Elizabeth Islands" OR "Victoria Island" OR "Banks Island" OR "Wrangel Island" OR "Seward peninsula" OR "Hudson Bay") OR ("Siberia" OR "Siberian" OR "Lake Baikal" OR "Lena River" OR "New Siberian Islands" OR "Severnaya Zemlya" OR "Franz Josef Land" OR "Novaya Zemlya" OR "Chukotka Autonomous Okrug" OR "Kamchatka Krai" OR "Magadan Oblast" OR "Murmansk Oblast" OR "Sakha" OR "Arkhangelsk Oblast" OR "Irkutsk Oblast" OR "Khabarovsk Krai" OR "Komi Republic" OR "Krasnoyarsk Krai" OR "Republic of Karelia" OR "Sakhalin Oblast" OR "Tuva" OR "Tyumen Oblast" OR "Polar Urals" OR "Yamalia") OR ("Svalbard" OR "Spitsbergen" OR "Bjørnøya" OR "Jan Mayen" OR "Finnmark" OR "Troms" OR "Lapland" or "Lappi" OR "Grímsey" OR "Lappland" OR "Norrbotten" OR "Västerbotten" OR "North West Europe" OR "Northwest Europe" OR "north* Europe" OR "Scandes" OR "Kola Peninsula") OR ("beringia" OR "beringian") OR ("global data*")

) OR AB=(

("arctic" OR "high-latitude" OR "oro-arctic" OR "North Polar region" OR "subarctic" OR "northern environ*") OR ("Canada" OR "Canadian" OR "Russia" OR "Russian Federation" OR "USSR" OR "RSFSR" OR "Russian Federation" OR "Russian" OR "Norway" OR "Norwegian" OR "Finland" OR "Finnish" OR "Sweden" OR "Iceland" OR "Icelandic" OR "Greenland" OR "Greenlandic" OR "Faroe") OR ("tundra" OR "permafrost" OR "mammoth steppe") OR ("Yukon Territory" OR "Northwest Territories" OR "Nunavut" OR "Baffin Island" OR "Belcher Islands" OR "Ellesmere Island" OR "Southampton Island" OR "Mackenzie River" OR "Great Slave Lake" OR "Great Bear Lake" OR "Yukon River" OR "Alaska" OR "Teshekpuk Lake" OR "Queen Elizabeth Islands" OR "Victoria Island" OR "Banks Island" OR "Wrangel Island" OR "Seward peninsula" OR "Hudson Bay") OR ("Siberia" OR "Siberian" OR "Lake Baikal" OR "Lena River" OR "New Siberian Islands" OR "Severnaya Zemlya" OR "Franz Josef Land" OR "Novaya Zemlya" OR "Chukotka Autonomous Okrug" OR "Kamchatka Krai" OR "Magadan Oblast" OR "Murmansk Oblast" OR "Sakha" OR "Arkhangelsk Oblast" OR "Irkutsk Oblast" OR "Khabarovsk Krai" OR "Komi Republic" OR "Krasnoyarsk Krai" OR "Republic of Karelia" OR "Sakhalin Oblast" OR "Tuva" OR "Tyumen Oblast" OR "Polar Urals" OR "Yamalia") OR ("Svalbard" OR "Spitsbergen" OR "Bjørnøya" OR "Jan Mayen" OR "Finnmark" OR "Troms" OR "Lapland" or "Lappi" OR "Grímsey" OR "Lappland" OR "Norrbotten" OR "Västerbotten" OR "North West Europe" OR "Northwest Europe" OR "north* Europe" OR "Scandes" OR "Kola Peninsula") OR ("beringia" OR "beringian") OR ("global data*")

) OR DE=(

("arctic" OR "high-latitude" OR "oro-arctic" OR "North Polar region" OR "subarctic" OR "northern environ*") OR ("Canada" OR "Canadian" OR "Russia" OR "Russian Federation" OR "USSR" OR "RSFSR" OR "Russian Federation" OR "Russian" OR "Norway" OR "Norwegian" OR "Finland" OR "Finnish" OR "Sweden" OR "Iceland" OR "Icelandic" OR "Greenland" OR "Greenlandic" OR "Faroe") OR ("tundra" OR "permafrost" OR "mammoth steppe") OR ("Yukon Territory" OR "Northwest Territories" OR "Nunavut" OR "Baffin Island" OR "Belcher Islands" OR "Ellesmere Island" OR "Southampton Island" OR "Mackenzie River" OR "Great Slave Lake" OR "Great Bear Lake" OR "Yukon River" OR "Alaska" OR "Teshekpuk Lake" OR "Queen Elizabeth Islands" OR "Victoria Island" OR "Banks Island" OR "Wrangel Island" OR "Seward peninsula" OR "Hudson Bay") OR ("Siberia" OR "Siberian" OR "Lake Baikal" OR "Lena River" OR "New Siberian Islands" OR "Severnaya Zemlya" OR "Franz Josef Land" OR "Novaya Zemlya" OR "Chukotka Autonomous Okrug" OR "Kamchatka Krai" OR "Magadan Oblast" OR "Murmansk Oblast" OR "Sakha" OR "Arkhangelsk Oblast" OR "Irkutsk Oblast" OR "Khabarovsk Krai" OR "Komi Republic" OR "Krasnoyarsk Krai" OR "Republic of Karelia" OR "Sakhalin Oblast" OR "Tuva" OR "Tyumen Oblast" OR "Polar Urals" OR "Yamalia") OR ("Svalbard" OR "Spitsbergen" OR "Bjørnøya" OR "Jan Mayen" OR "Finnmark" OR "Troms" OR "Lapland" or "Lappi" OR "Grímsey" OR "Lappland" OR "Norrbotten" OR "Västerbotten" OR "North West Europe" OR "Northwest Europe" OR "north* Europe" OR "Scandes" OR "Kola Peninsula") OR ("beringia" OR "beringian") OR ("global data*")

) OR GE=(

("arctic" OR "high-latitude" OR "oro-arctic" OR "North Polar region" OR "subarctic" OR "northern environ*") OR ("Canada" OR "Canadian" OR "Russia" OR "Russian Federation" OR "USSR" OR "RSFSR" OR "Russian Federation" OR "Russian" OR "Norway" OR "Norwegian" OR "Finland" OR "Finnish" OR "Sweden" OR "Iceland" OR "Icelandic" OR "Greenland" OR "Greenlandic" OR "Faroe") OR ("tundra" OR "permafrost" OR "mammoth steppe") OR ("Yukon Territory" OR "Northwest Territories" OR "Nunavut" OR "Baffin Island" OR "Belcher Islands" OR "Ellesmere Island" OR "Southampton Island" OR "Mackenzie River" OR "Great Slave Lake" OR "Great Bear Lake" OR "Yukon River" OR "Alaska" OR "Teshekpuk Lake" OR "Queen Elizabeth Islands" OR "Victoria Island" OR "Banks Island" OR "Wrangel Island" OR "Seward peninsula" OR "Hudson Bay") OR ("Siberia" OR "Siberian" OR "Lake Baikal" OR "Lena River" OR "New Siberian Islands" OR "Severnaya Zemlya" OR "Franz Josef Land" OR "Novaya Zemlya" OR "Chukotka Autonomous Okrug" OR "Kamchatka Krai" OR "Magadan Oblast" OR "Murmansk Oblast" OR "Sakha" OR "Arkhangelsk Oblast" OR "Irkutsk Oblast" OR "Khabarovsk Krai" OR "Komi Republic" OR "Krasnoyarsk Krai" OR "Republic of Karelia" OR "Sakhalin Oblast" OR "Tuva" OR "Tyumen Oblast" OR "Polar Urals" OR "Yamalia") OR ("Svalbard" OR "Spitsbergen" OR "Bjørnøya" OR "Jan Mayen" OR "Finnmark" OR "Troms" OR "Lapland" or "Lappi" OR "Grímsey" OR "Lappland" OR "Norrbotten" OR "Västerbotten" OR "North West Europe" OR "Northwest Europe" OR "north* Europe" OR "Scandes" OR "Kola Peninsula") OR ("beringia" OR "beringian") OR ("global data*")

))

AND

(TI=(

("reconstruct" OR "reconstruction" OR "reconstructed") OR ("pal$eo*" OR "micropal$entol*") OR ("arch$eolog*" OR "artefact") OR ("history" OR "historic site" OR "historical record") OR ("dendrochron*" OR "dendroclim*" OR "dendroeco*" OR "growth ring" OR "tree ring" OR "wood ring") OR ("radiocarbon" OR "radio-carbon" OR "AMS" OR "accelerator mass spectrometry") OR ("age determination" OR "years before present" OR "yr BP" OR "calibration of age" OR "age model" OR "age-depth model") OR ("1?,??? 14C yr B$P" OR "1? ??? 14C Yr B$P" OR "1?,??? yr B$P" OR "1? ??? yr B$P" OR "1?,??? cal yr B$P" OR "1? ??? cal yr B$P" OR "pre-1???" OR "pre-20th century" OR "1?th century" OR "last ?,??? years" OR "last 1?,??? years" OR "14C dates" OR "1? Cal Ka" OR "one century" OR "pre-industrial" OR "preindustrial") OR ("fossil" OR "sub-fossil" OR "macrofossil" OR "megafossil" OR "microfossil" OR "subfossil") OR ("palynolog*") OR ("prehistoric" OR "prehistory" OR "Iron Age" OR "Bronze Age" OR "Neolithic" OR "Mesolithic" OR "Late Upper Pal$eolithic" OR "BC") OR ("Holocene" OR "MidHolocene" OR "Mid-Holocene" OR "late Pleistocene" OR "end of the Pleistocene" OR "Late Quaternary" OR "Little Ice Age" OR "Medieval Climate Anomoly" OR "Younger Dryas" OR "MIS 1" OR "marine isotope stage 1") OR ("centennial" OR "centuries" OR "millenial" OR "millenia" OR "submillenial" OR "chronolog*" OR "temporal trend") OR ("preboreal chronozone" OR "boreal chronozone" OR "atlantic chronozone" OR "subboreal chronozone") OR ("sediment core" OR "sedimentary sequence" OR "lake sediment" OR "transfer function" OR "peat core" OR "peat bog core" OR "peat monolith") OR ("ancient DNA" OR "aDNA" OR "sedDNA" OR "sedimentary DNA") OR ("postglacial" OR "post-glacial" OR "deglaciation" OR "deglacial") OR ("last appearance date" OR "refugia") OR ("multiproxy" OR "proxy record" OR "proxy archive" OR "proxy" OR "proxy study" OR "proxy studies") OR ("long-term ecolog*" OR "long-term record")

) OR TS=(

("reconstruct" OR "reconstruction" OR "reconstructed") OR ("pal$eo*" OR "micropal$entol*") OR ("arch$eolog*" OR "artefact") OR ("history" OR "historic site" OR "historical record") OR ("dendrochron*" OR "dendroclim*" OR "dendroeco*" OR "growth ring" OR "tree ring" OR "wood ring") OR ("radiocarbon" OR "radio-carbon" OR "AMS" OR "accelerator mass spectrometry") OR ("age determination" OR "years before present" OR "yr BP" OR "calibration of age" OR "age model" OR "age-depth model") OR ("1?,??? 14C yr B$P" OR "1? ??? 14C Yr B$P" OR "1?,??? yr B$P" OR "1? ??? yr B$P" OR "1?,??? cal yr B$P" OR "1? ??? cal yr B$P" OR "pre-1???" OR "pre-20th century" OR "1?th century" OR "last ?,??? years" OR "last 1?,??? years" OR "14C dates" OR "1? Cal Ka" OR "one century" OR "pre-industrial" OR "preindustrial") OR ("fossil" OR "sub-fossil" OR "macrofossil" OR "megafossil" OR "microfossil" OR "subfossil") OR ("palynolog*") OR ("prehistoric" OR "prehistory" OR "Iron Age" OR "Bronze Age" OR "Neolithic" OR "Mesolithic" OR "Late Upper Pal$eolithic" OR "BC") OR ("Holocene" OR "MidHolocene" OR "Mid-Holocene" OR "late Pleistocene" OR "end of the Pleistocene" OR "Late Quaternary" OR "Little Ice Age" OR "Medieval Climate Anomoly" OR "Younger Dryas" OR "MIS 1" OR "marine isotope stage 1") OR ("centennial" OR "centuries" OR "millenial" OR "millenia" OR "submillenial" OR "chronolog*" OR "temporal trend") OR ("preboreal chronozone" OR "boreal chronozone" OR "atlantic chronozone" OR "subboreal chronozone") OR ("sediment core" OR "sedimentary sequence" OR "lake sediment" OR "transfer function" OR "peat core" OR "peat bog core" OR "peat monolith") OR ("ancient DNA" OR "aDNA" OR "sedDNA" OR "sedimentary DNA") OR ("postglacial" OR "post-glacial" OR "deglaciation" OR "deglacial") OR ("last appearance date" OR "refugia") OR ("multiproxy" OR "proxy record" OR "proxy archive" OR "proxy" OR "proxy study" OR "proxy studies") OR ("long-term ecolog*" OR "long-term record")

) OR CC=(

("reconstruct" OR "reconstruction" OR "reconstructed") OR ("pal$eo*" OR "micropal$entol*") OR ("arch$eolog*" OR "artefact") OR ("history" OR "historic site" OR "historical record") OR ("dendrochron*" OR "dendroclim*" OR "dendroeco*" OR "growth ring" OR "tree ring" OR "wood ring") OR ("radiocarbon" OR "radio-carbon" OR "AMS" OR "accelerator mass spectrometry") OR ("age determination" OR "years before present" OR "yr BP" OR "calibration of age" OR "age model" OR "age-depth model") OR ("1?,??? 14C yr B$P" OR "1? ??? 14C Yr B$P" OR "1?,??? yr B$P" OR "1? ??? yr B$P" OR "1?,??? cal yr B$P" OR "1? ??? cal yr B$P" OR "pre-1???" OR "pre-20th century" OR "1?th century" OR "last ?,??? years" OR "last 1?,??? years" OR "14C dates" OR "1? Cal Ka" OR "one century" OR "pre-industrial" OR "preindustrial") OR ("fossil" OR "sub-fossil" OR "macrofossil" OR "megafossil" OR "microfossil" OR "subfossil") OR ("palynolog*") OR ("prehistoric" OR "prehistory" OR "Iron Age" OR "Bronze Age" OR "Neolithic" OR "Mesolithic" OR "Late Upper Pal$eolithic" OR "BC") OR ("Holocene" OR "MidHolocene" OR "Mid-Holocene" OR "late Pleistocene" OR "end of the Pleistocene" OR "Late Quaternary" OR "Little Ice Age" OR "Medieval Climate Anomoly" OR "Younger Dryas" OR "MIS 1" OR "marine isotope stage 1") OR ("centennial" OR "centuries" OR "millenial" OR "millenia" OR "submillenial" OR "chronolog*" OR "temporal trend") OR ("preboreal chronozone" OR "boreal chronozone" OR "atlantic chronozone" OR "subboreal chronozone") OR ("sediment core" OR "sedimentary sequence" OR "lake sediment" OR "transfer function" OR "peat core" OR "peat bog core" OR "peat monolith") OR ("ancient DNA" OR "aDNA" OR "sedDNA" OR "sedimentary DNA") OR ("postglacial" OR "post-glacial" OR "deglaciation" OR "deglacial") OR ("last appearance date" OR "refugia") OR ("multiproxy" OR "proxy record" OR "proxy archive" OR "proxy" OR "proxy study" OR "proxy studies") OR ("long-term ecolog*" OR "long-term record")

) OR AB=(

("reconstruct" OR "reconstruction" OR "reconstructed") OR ("pal$eo*" OR "micropal$entol*") OR ("arch$eolog*" OR "artefact") OR ("history" OR "historic site" OR "historical record") OR ("dendrochron*" OR "dendroclim*" OR "dendroeco*" OR "growth ring" OR "tree ring" OR "wood ring") OR ("radiocarbon" OR "radio-carbon" OR "AMS" OR "accelerator mass spectrometry") OR ("age determination" OR "years before present" OR "yr BP" OR "calibration of age" OR "age model" OR "age-depth model") OR ("1?,??? 14C yr B$P" OR "1? ??? 14C Yr B$P" OR "1?,??? yr B$P" OR "1? ??? yr B$P" OR "1?,??? cal yr B$P" OR "1? ??? cal yr B$P" OR "pre-1???" OR "pre-20th century" OR "1?th century" OR "last ?,??? years" OR "last 1?,??? years" OR "14C dates" OR "1? Cal Ka" OR "one century" OR "pre-industrial" OR "preindustrial") OR ("fossil" OR "sub-fossil" OR "macrofossil" OR "megafossil" OR "microfossil" OR "subfossil") OR ("palynolog*") OR ("prehistoric" OR "prehistory" OR "Iron Age" OR "Bronze Age" OR "Neolithic" OR "Mesolithic" OR "Late Upper Pal$eolithic" OR "BC") OR ("Holocene" OR "MidHolocene" OR "Mid-Holocene" OR "late Pleistocene" OR "end of the Pleistocene" OR "Late Quaternary" OR "Little Ice Age" OR "Medieval Climate Anomoly" OR "Younger Dryas" OR "MIS 1" OR "marine isotope stage 1") OR ("centennial" OR "centuries" OR "millenial" OR "millenia" OR "submillenial" OR "chronolog*" OR "temporal trend") OR ("preboreal chronozone" OR "boreal chronozone" OR "atlantic chronozone" OR "subboreal chronozone") OR ("sediment core" OR "sedimentary sequence" OR "lake sediment" OR "transfer function" OR "peat core" OR "peat bog core" OR "peat monolith") OR ("ancient DNA" OR "aDNA" OR "sedDNA" OR "sedimentary DNA") OR ("postglacial" OR "post-glacial" OR "deglaciation" OR "deglacial") OR ("last appearance date" OR "refugia") OR ("multiproxy" OR "proxy record" OR "proxy archive" OR "proxy" OR "proxy study" OR "proxy studies") OR ("long-term ecolog*" OR "long-term record")

) OR DE=(

("reconstruct" OR "reconstruction" OR "reconstructed") OR ("pal$eo*" OR "micropal$entol*") OR ("arch$eolog*" OR "artefact") OR ("history" OR "historic site" OR "historical record") OR ("dendrochron*" OR "dendroclim*" OR "dendroeco*" OR "growth ring" OR "tree ring" OR "wood ring") OR ("radiocarbon" OR "radio-carbon" OR "AMS" OR "accelerator mass spectrometry") OR ("age determination" OR "years before present" OR "yr BP" OR "calibration of age" OR "age model" OR "age-depth model") OR ("1?,??? 14C yr B$P" OR "1? ??? 14C Yr B$P" OR "1?,??? yr B$P" OR "1? ??? yr B$P" OR "1?,??? cal yr B$P" OR "1? ??? cal yr B$P" OR "pre-1???" OR "pre-20th century" OR "1?th century" OR "last ?,??? years" OR "last 1?,??? years" OR "14C dates" OR "1? Cal Ka" OR "one century" OR "pre-industrial" OR "preindustrial") OR ("fossil" OR "sub-fossil" OR "macrofossil" OR "megafossil" OR "microfossil" OR "subfossil") OR ("palynolog*") OR ("prehistoric" OR "prehistory" OR "Iron Age" OR "Bronze Age" OR "Neolithic" OR "Mesolithic" OR "Late Upper Pal$eolithic" OR "BC") OR ("Holocene" OR "MidHolocene" OR "Mid-Holocene" OR "late Pleistocene" OR "end of the Pleistocene" OR "Late Quaternary" OR "Little Ice Age" OR "Medieval Climate Anomoly" OR "Younger Dryas" OR "MIS 1" OR "marine isotope stage 1") OR ("centennial" OR "centuries" OR "millenial" OR "millenia" OR "submillenial" OR "chronolog*" OR "temporal trend") OR ("preboreal chronozone" OR "boreal chronozone" OR "atlantic chronozone" OR "subboreal chronozone") OR ("sediment core" OR "sedimentary sequence" OR "lake sediment" OR "transfer function" OR "peat core" OR "peat bog core" OR "peat monolith") OR ("ancient DNA" OR "aDNA" OR "sedDNA" OR "sedimentary DNA") OR ("postglacial" OR "post-glacial" OR "deglaciation" OR "deglacial") OR ("last appearance date" OR "refugia") OR ("multiproxy" OR "proxy record" OR "proxy archive" OR "proxy" OR "proxy study" OR "proxy studies") OR ("long-term ecolog*" OR "long-term record")

) OR GT=(

("reconstruct" OR "reconstruction" OR "reconstructed") OR ("pal$eo*" OR "micropal$entol*") OR ("arch$eolog*" OR "artefact") OR ("history" OR "historic site" OR "historical record") OR ("dendrochron*" OR "dendroclim*" OR "dendroeco*" OR "growth ring" OR "tree ring" OR "wood ring") OR ("radiocarbon" OR "radio-carbon" OR "AMS" OR "accelerator mass spectrometry") OR ("age determination" OR "years before present" OR "yr BP" OR "calibration of age" OR "age model" OR "age-depth model") OR ("1?,??? 14C yr B$P" OR "1? ??? 14C Yr B$P" OR "1?,??? yr B$P" OR "1? ??? yr B$P" OR "1?,??? cal yr B$P" OR "1? ??? cal yr B$P" OR "pre-1???" OR "pre-20th century" OR "1?th century" OR "last ?,??? years" OR "last 1?,??? years" OR "14C dates" OR "1? Cal Ka" OR "one century" OR "pre-industrial" OR "preindustrial") OR ("fossil" OR "sub-fossil" OR "macrofossil" OR "megafossil" OR "microfossil" OR "subfossil") OR ("palynolog*") OR ("prehistoric" OR "prehistory" OR "Iron Age" OR "Bronze Age" OR "Neolithic" OR "Mesolithic" OR "Late Upper Pal$eolithic" OR "BC") OR ("Holocene" OR "MidHolocene" OR "Mid-Holocene" OR "late Pleistocene" OR "end of the Pleistocene" OR "Late Quaternary" OR "Little Ice Age" OR "Medieval Climate Anomoly" OR "Younger Dryas" OR "MIS 1" OR "marine isotope stage 1") OR ("centennial" OR "centuries" OR "millenial" OR "millenia" OR "submillenial" OR "chronolog*" OR "temporal trend") OR ("preboreal chronozone" OR "boreal chronozone" OR "atlantic chronozone" OR "subboreal chronozone") OR ("sediment core" OR "sedimentary sequence" OR "lake sediment" OR "transfer function" OR "peat core" OR "peat bog core" OR "peat monolith") OR ("ancient DNA" OR "aDNA" OR "sedDNA" OR "sedimentary DNA") OR ("postglacial" OR "post-glacial" OR "deglaciation" OR "deglacial") OR ("last appearance date" OR "refugia") OR ("multiproxy" OR "proxy record" OR "proxy archive" OR "proxy" OR "proxy study" OR "proxy studies") OR ("long-term ecolog*" OR "long-term record")

))

AND

(TI=(

("plant?" OR "flora" OR "vegetation" OR "Plantae" OR "vegetational" OR "pal?eovegetation" OR "floristic") OR ("bryophyte" OR "Bryophyta" OR "liverwort?" OR "moss" OR "mosses") OR ("shrub?" OR "woody plant" OR "vascular plant" OR "fern?" OR "pteridophyt?" OR "spermatopht?" OR "xerophyte" OR "tree species" OR "coniferous" OR "conifer" OR "deciduous" OR "herb" OR "forb?") OR ("forest species" OR "treeline species" OR "tree-line species" OR "larch" OR "Larix" OR "spruce" OR "picea" OR "western hemlock" OR "Tsuga" OR "Sitka" OR "alnus" OR "alder" OR "cottonwood" OR "Populus" OR "poplar" OR "Pinus" OR "pine" OR "Betula" OR "birch" OR "willow" OR "salix") OR ("lichen?" OR "sporomiella" OR "dung fungal spore" OR "fungi" OR "fungal") OR ("diatom" OR "Bacillariophyceae") OR ("Mollusca" OR "mollusk" OR "mollusc" OR "Gastropoda" OR "gastropod" OR "Bivalvia" OR "bivalve") OR ("mammal" OR "mammalian" OR "mammoth" OR "reindeer" OR "caribou" OR "Rangifer tarandus" OR "polar bear" OR "Mammuthus primigenius" OR "steppe bison" OR "Bison priscus" OR "muskox" OR "Ovibos moschatus" OR "moose" OR "Alcos alces" OR "horse" OR "Equus caballus" OR "wolf" OR "wolves" OR "Canis lupus" OR "dog" OR "squirrel" OR "rodent" OR "Rodentia" OR "Urocitellus" OR "arctic fox" OR "vulpes lagopus" OR "alopex lagopus" OR "reptile" OR "bird?" OR "Aves") OR ("ostracod" OR "ostracode" OR "ostracoda") OR ("coleoptera" OR "beetle") OR ("chironomid" OR "Chironomidae") OR ("insect" OR "macroinvertebrate" OR "macro-invertebrate") OR ("amphibian" OR "Amphibia" OR "aquatic animal" OR "algae" OR "algal") OR ("animal" OR "fauna" OR "faunal" OR "megafauna" OR "megaherbivore" OR "vertebrata" OR "vertebrate") OR ("pollen" OR "palynology" OR "macrofossil" OR "pal$eobotanical" OR "leaf wax" OR "palynomorph") OR ("organic biomarker") OR ("ring width" OR "wood ring" OR "tree ring" OR "stem sample" OR "driftwood") OR ("terrestrial proxies" OR "terrestrial proxy") ) OR TS=(

("plant?" OR "flora" OR "vegetation" OR "Plantae" OR "vegetational" OR "pal?eovegetation" OR "floristic") OR ("bryophyte" OR "Bryophyta" OR "liverwort?" OR "moss" OR "mosses") OR ("shrub?" OR "woody plant" OR "vascular plant" OR "fern?" OR "pteridophyt?" OR "spermatopht?" OR "xerophyte" OR "tree species" OR "coniferous" OR "conifer" OR "deciduous" OR "herb" OR "forb?") OR ("forest species" OR "treeline species" OR "tree-line species" OR "larch" OR "Larix" OR "spruce" OR "picea" OR "western hemlock" OR "Tsuga" OR "Sitka" OR "alnus" OR "alder" OR "cottonwood" OR "Populus" OR "poplar" OR "Pinus" OR "pine" OR "Betula" OR "birch" OR "willow" OR "salix") OR ("lichen?" OR "sporomiella" OR "dung fungal spore" OR "fungi" OR "fungal") OR ("diatom" OR "Bacillariophyceae") OR ("Mollusca" OR "mollusk" OR "mollusc" OR "Gastropoda" OR "gastropod" OR "Bivalvia" OR "bivalve") OR ("mammal" OR "mammalian" OR "mammoth" OR "reindeer" OR "caribou" OR "Rangifer tarandus" OR "polar bear" OR "Mammuthus primigenius" OR "steppe bison" OR "Bison priscus" OR "muskox" OR "Ovibos moschatus" OR "moose" OR "Alcos alces" OR "horse" OR "Equus caballus" OR "wolf" OR "wolves" OR "Canis lupus" OR "dog" OR "squirrel" OR "rodent" OR "Rodentia" OR "Urocitellus" OR "arctic fox" OR "vulpes lagopus" OR "alopex lagopus" OR "reptile" OR "bird?" OR "Aves") OR ("ostracod" OR "ostracode" OR "ostracoda") OR ("coleoptera" OR "beetle") OR ("chironomid" OR "Chironomidae") OR ("insect" OR "macroinvertebrate" OR "macro-invertebrate") OR ("amphibian" OR "Amphibia" OR "aquatic animal" OR "algae" OR "algal") OR ("animal" OR "fauna" OR "faunal" OR "megafauna" OR "megaherbivore" OR "vertebrata" OR "vertebrate") OR ("pollen" OR "palynology" OR "macrofossil" OR "pal$eobotanical" OR "leaf wax" OR "palynomorph") OR ("organic biomarker") OR ("ring width" OR "wood ring" OR "tree ring" OR "stem sample" OR "driftwood") OR ("terrestrial proxies" OR "terrestrial proxy")

) OR CC=(

("plant?" OR "flora" OR "vegetation" OR "Plantae" OR "vegetational" OR "pal?eovegetation" OR "floristic") OR ("bryophyte" OR "Bryophyta" OR "liverwort?" OR "moss" OR "mosses") OR ("shrub?" OR "woody plant" OR "vascular plant" OR "fern?" OR "pteridophyt?" OR "spermatopht?" OR "xerophyte" OR "tree species" OR "coniferous" OR "conifer" OR "deciduous" OR "herb" OR "forb?") OR ("forest species" OR "treeline species" OR "tree-line species" OR "larch" OR "Larix" OR "spruce" OR "picea" OR "western hemlock" OR "Tsuga" OR "Sitka" OR "alnus" OR "alder" OR "cottonwood" OR "Populus" OR "poplar" OR "Pinus" OR "pine" OR "Betula" OR "birch" OR "willow" OR "salix") OR ("lichen?" OR "sporomiella" OR "dung fungal spore" OR "fungi" OR "fungal") OR ("diatom" OR "Bacillariophyceae") OR ("Mollusca" OR "mollusk" OR "mollusc" OR "Gastropoda" OR "gastropod" OR "Bivalvia" OR "bivalve") OR ("mammal" OR "mammalian" OR "mammoth" OR "reindeer" OR "caribou" OR "Rangifer tarandus" OR "polar bear" OR "Mammuthus primigenius" OR "steppe bison" OR "Bison priscus" OR "muskox" OR "Ovibos moschatus" OR "moose" OR "Alcos alces" OR "horse" OR "Equus caballus" OR "wolf" OR "wolves" OR "Canis lupus" OR "dog" OR "squirrel" OR "rodent" OR "Rodentia" OR "Urocitellus" OR "arctic fox" OR "vulpes lagopus" OR "alopex lagopus" OR "reptile" OR "bird?" OR "Aves") OR ("ostracod" OR "ostracode" OR "ostracoda") OR ("coleoptera" OR "beetle") OR ("chironomid" OR "Chironomidae") OR ("insect" OR "macroinvertebrate" OR "macro-invertebrate") OR ("amphibian" OR "Amphibia" OR "aquatic animal" OR "algae" OR "algal") OR ("animal" OR "fauna" OR "faunal" OR "megafauna" OR "megaherbivore" OR "vertebrata" OR "vertebrate") OR ("pollen" OR "palynology" OR "macrofossil" OR "pal$eobotanical" OR "leaf wax" OR "palynomorph") OR ("organic biomarker") OR ("ring width" OR "wood ring" OR "tree ring" OR "stem sample" OR "driftwood") OR ("terrestrial proxies" OR "terrestrial proxy")

) OR AB=(

("plant?" OR "flora" OR "vegetation" OR "Plantae" OR "vegetational" OR "pal?eovegetation" OR "floristic") OR ("bryophyte" OR "Bryophyta" OR "liverwort?" OR "moss" OR "mosses") OR ("shrub?" OR "woody plant" OR "vascular plant" OR "fern?" OR "pteridophyt?" OR "spermatopht?" OR "xerophyte" OR "tree species" OR "coniferous" OR "conifer" OR "deciduous" OR "herb" OR "forb?") OR ("forest species" OR "treeline species" OR "tree-line species" OR "larch" OR "Larix" OR "spruce" OR "picea" OR "western hemlock" OR "Tsuga" OR "Sitka" OR "alnus" OR "alder" OR "cottonwood" OR "Populus" OR "poplar" OR "Pinus" OR "pine" OR "Betula" OR "birch" OR "willow" OR "salix") OR ("lichen?" OR "sporomiella" OR "dung fungal spore" OR "fungi" OR "fungal") OR ("diatom" OR "Bacillariophyceae") OR ("Mollusca" OR "mollusk" OR "mollusc" OR "Gastropoda" OR "gastropod" OR "Bivalvia" OR "bivalve") OR ("mammal" OR "mammalian" OR "mammoth" OR "reindeer" OR "caribou" OR "Rangifer tarandus" OR "polar bear" OR "Mammuthus primigenius" OR "steppe bison" OR "Bison priscus" OR "muskox" OR "Ovibos moschatus" OR "moose" OR "Alcos alces" OR "horse" OR "Equus caballus" OR "wolf" OR "wolves" OR "Canis lupus" OR "dog" OR "squirrel" OR "rodent" OR "Rodentia" OR "Urocitellus" OR "arctic fox" OR "vulpes lagopus" OR "alopex lagopus" OR "reptile" OR "bird?" OR "Aves") OR ("ostracod" OR "ostracode" OR "ostracoda") OR ("coleoptera" OR "beetle") OR ("chironomid" OR "Chironomidae") OR ("insect" OR "macroinvertebrate" OR "macro-invertebrate") OR ("amphibian" OR "Amphibia" OR "aquatic animal" OR "algae" OR "algal") OR ("animal" OR "fauna" OR "faunal" OR "megafauna" OR "megaherbivore" OR "vertebrata" OR "vertebrate") OR ("pollen" OR "palynology" OR "macrofossil" OR "pal$eobotanical" OR "leaf wax" OR "palynomorph") OR ("organic biomarker") OR ("ring width" OR "wood ring" OR "tree ring" OR "stem sample" OR "driftwood") OR ("terrestrial proxies" OR "terrestrial proxy")

) OR DE=(

("plant?" OR "flora" OR "vegetation" OR "Plantae" OR "vegetational" OR "pal?eovegetation" OR "floristic") OR ("bryophyte" OR "Bryophyta" OR "liverwort?" OR "moss" OR "mosses") OR ("shrub?" OR "woody plant" OR "vascular plant" OR "fern?" OR "pteridophyt?" OR "spermatopht?" OR "xerophyte" OR "tree species" OR "coniferous" OR "conifer" OR "deciduous" OR "herb" OR "forb?") OR ("forest species" OR "treeline species" OR "tree-line species" OR "larch" OR "Larix" OR "spruce" OR "picea" OR "western hemlock" OR "Tsuga" OR "Sitka" OR "alnus" OR "alder" OR "cottonwood" OR "Populus" OR "poplar" OR "Pinus" OR "pine" OR "Betula" OR "birch" OR "willow" OR "salix") OR ("lichen?" OR "sporomiella" OR "dung fungal spore" OR "fungi" OR "fungal") OR ("diatom" OR "Bacillariophyceae") OR ("Mollusca" OR "mollusk" OR "mollusc" OR "Gastropoda" OR "gastropod" OR "Bivalvia" OR "bivalve") OR ("mammal" OR "mammalian" OR "mammoth" OR "reindeer" OR "caribou" OR "Rangifer tarandus" OR "polar bear" OR "Mammuthus primigenius" OR "steppe bison" OR "Bison priscus" OR "muskox" OR "Ovibos moschatus" OR "moose" OR "Alcos alces" OR "horse" OR "Equus caballus" OR "wolf" OR "wolves" OR "Canis lupus" OR "dog" OR "squirrel" OR "rodent" OR "Rodentia" OR "Urocitellus" OR "arctic fox" OR "vulpes lagopus" OR "alopex lagopus" OR "reptile" OR "bird?" OR "Aves") OR ("ostracod" OR "ostracode" OR "ostracoda") OR ("coleoptera" OR "beetle") OR ("chironomid" OR "Chironomidae") OR ("insect" OR "macroinvertebrate" OR "macro-invertebrate") OR ("amphibian" OR "Amphibia" OR "aquatic animal" OR "algae" OR "algal") OR ("animal" OR "fauna" OR "faunal" OR "megafauna" OR "megaherbivore" OR "vertebrata" OR "vertebrate") OR ("pollen" OR "palynology" OR "macrofossil" OR "pal$eobotanical" OR "leaf wax" OR "palynomorph") OR ("organic biomarker") OR ("ring width" OR "wood ring" OR "tree ring" OR "stem sample" OR "driftwood") OR ("terrestrial proxies" OR "terrestrial proxy")

) OR TA=(

("plant?" OR "flora" OR "vegetation" OR "Plantae" OR "vegetational" OR "pal?eovegetation" OR "floristic") OR ("bryophyte" OR "Bryophyta" OR "liverwort?" OR "moss" OR "mosses") OR ("shrub?" OR "woody plant" OR "vascular plant" OR "fern?" OR "pteridophyt?" OR "spermatopht?" OR "xerophyte" OR "tree species" OR "coniferous" OR "conifer" OR "deciduous" OR "herb" OR "forb?") OR ("forest species" OR "treeline species" OR "tree-line species" OR "larch" OR "Larix" OR "spruce" OR "picea" OR "western hemlock" OR "Tsuga" OR "Sitka" OR "alnus" OR "alder" OR "cottonwood" OR "Populus" OR "poplar" OR "Pinus" OR "pine" OR "Betula" OR "birch" OR "willow" OR "salix") OR ("lichen?" OR "sporomiella" OR "dung fungal spore" OR "fungi" OR "fungal") OR ("diatom" OR "Bacillariophyceae") OR ("Mollusca" OR "mollusk" OR "mollusc" OR "Gastropoda" OR "gastropod" OR "Bivalvia" OR "bivalve") OR ("mammal" OR "mammalian" OR "mammoth" OR "reindeer" OR "caribou" OR "Rangifer tarandus" OR "polar bear" OR "Mammuthus primigenius" OR "steppe bison" OR "Bison priscus" OR "muskox" OR "Ovibos moschatus" OR "moose" OR "Alcos alces" OR "horse" OR "Equus caballus" OR "wolf" OR "wolves" OR "Canis lupus" OR "dog" OR "squirrel" OR "rodent" OR "Rodentia" OR "Urocitellus" OR "arctic fox" OR "vulpes lagopus" OR "alopex lagopus" OR "reptile" OR "bird?" OR "Aves") OR ("ostracod" OR "ostracode" OR "ostracoda") OR ("coleoptera" OR "beetle") OR ("chironomid" OR "Chironomidae") OR ("insect" OR "macroinvertebrate" OR "macro-invertebrate") OR ("amphibian" OR "Amphibia" OR "aquatic animal" OR "algae" OR "algal") OR ("animal" OR "fauna" OR "faunal" OR "megafauna" OR "megaherbivore" OR "vertebrata" OR "vertebrate") OR ("pollen" OR "palynology" OR "macrofossil" OR "pal$eobotanical" OR "leaf wax" OR "palynomorph") OR ("organic biomarker") OR ("ring width" OR "wood ring" OR "tree ring" OR "stem sample" OR "driftwood") OR ("terrestrial proxies" OR "terrestrial proxy")

))

## CAB Abstracts via Ovid

Searches 1 to 4 combined using AND:

1. ("arctic" or "high-latitude" or "oro-arctic" or "North Polar region" or "subarctic" or "northern environ*" or ("Canada" or "Canadian" or "Russia" or "Russian Federation" or "USSR" or "RSFSR" or "Russian Federation" or "Russian" or "Norway" or "Norwegian" or "Finland" or "Finnish" or "Sweden" or "Iceland" or "Icelandic" or "Greenland" or "Greenlandic" or "Faroe") or ("tundra" or "permafrost" or "mammoth steppe") or ("Yukon Territory" or "Northwest Territories" or "Nunavut" or "Baffin Island" or "Belcher Islands" or "Ellesmere Island" or "Southampton Island" or "Mackenzie River" or "Great Slave Lake" or "Great Bear Lake" or "Yukon River" or "Alaska" or "Teshekpuk Lake" or "Queen Elizabeth Islands" or "Victoria Island" or "Banks Island" or "Wrangel Island" or "Seward peninsula" or "Hudson Bay") or ("Siberia" or "Siberian" or "Lake Baikal" or "Lena River" or "New Siberian Islands" or "Severnaya Zemlya" or "Franz Josef Land" or "Novaya Zemlya" or "Chukotka Autonomous Okrug" or "Kamchatka Krai" or "Magadan Oblast" or "Murmansk Oblast" or "Sakha" or "Arkhangelsk Oblast" or "Irkutsk Oblast" or "Khabarovsk Krai" or "Komi Republic" or "Krasnoyarsk Krai" or "Republic of Karelia" or "Sakhalin Oblast" or "Tuva" or "Tyumen Oblast" or "Polar Urals" or "Yamalia") or ("Svalbard" or "Spitsbergen" or "Bjørnøya" or "Jan Mayen" or "Finnmark" or "Troms" or "Lapland" or "Lappi" or "Grímsey" or "Lappland" or "Norrbotten" or "Västerbotten" or "North West Europe" or "Northwest Europe" or "north* Europe" or "Scandes" or "Kola Peninsula") or ("beringia" or "beringian") or "global data*").ti. or ("arctic" or "high-latitude" or "oro-arctic" or "North Polar region" or "subarctic" or "northern environ*" or ("Canada" or "Canadian" or "Russia" or "Russian Federation" or "USSR" or "RSFSR" or "Russian Federation" or "Russian" or "Norway" or "Norwegian" or "Finland" or "Finnish" or "Sweden" or "Iceland" or "Icelandic" or "Greenland" or "Greenlandic" or "Faroe") or ("tundra" or "permafrost" or "mammoth steppe") or ("Yukon Territory" or "Northwest Territories" or "Nunavut" or "Baffin Island" or "Belcher Islands" or "Ellesmere Island" or "Southampton Island" or "Mackenzie River" or "Great Slave Lake" or "Great Bear Lake" or "Yukon River" or "Alaska" or "Teshekpuk Lake" or "Queen Elizabeth Islands" or "Victoria Island" or "Banks Island" or "Wrangel Island" or "Seward peninsula" or "Hudson Bay") or ("Siberia" or "Siberian" or "Lake Baikal" or "Lena River" or "New Siberian Islands" or "Severnaya Zemlya" or "Franz Josef Land" or "Novaya Zemlya" or "Chukotka Autonomous Okrug" or "Kamchatka Krai" or "Magadan Oblast" or "Murmansk Oblast" or "Sakha" or "Arkhangelsk Oblast" or "Irkutsk Oblast" or "Khabarovsk Krai" or "Komi Republic" or "Krasnoyarsk Krai" or "Republic of Karelia" or "Sakhalin Oblast" or "Tuva" or "Tyumen Oblast" or "Polar Urals" or "Yamalia") or ("Svalbard" or "Spitsbergen" or "Bjørnøya" or "Jan Mayen" or "Finnmark" or "Troms" or "Lapland" or "Lappi" or "Grímsey" or "Lappland" or "Norrbotten" or "Västerbotten" or "North West Europe" or "Northwest Europe" or "north* Europe" or "Scandes" or "Kola Peninsula") or ("beringia" or "beringian") or "global data*").gl. or ("arctic" or "high-latitude" or "oro-arctic" or "North Polar region" or "subarctic" or "northern environ*" or ("Canada" or "Canadian" or "Russia" or "Russian Federation" or "USSR" or "RSFSR" or "Russian Federation" or "Russian" or "Norway" or "Norwegian" or "Finland" or "Finnish" or "Sweden" or "Iceland" or "Icelandic" or "Greenland" or "Greenlandic" or "Faroe") or ("tundra" or "permafrost" or "mammoth steppe") or ("Yukon Territory" or "Northwest Territories" or "Nunavut" or "Baffin Island" or "Belcher Islands" or "Ellesmere Island" or "Southampton Island" or "Mackenzie River" or "Great Slave Lake" or "Great Bear Lake" or "Yukon River" or "Alaska" or "Teshekpuk Lake" or "Queen Elizabeth Islands" or "Victoria Island" or "Banks Island" or "Wrangel Island" or "Seward peninsula" or "Hudson Bay") or ("Siberia" or "Siberian" or "Lake Baikal" or "Lena River" or "New Siberian Islands" or "Severnaya Zemlya" or "Franz Josef Land" or "Novaya Zemlya" or "Chukotka Autonomous Okrug" or "Kamchatka Krai" or "Magadan Oblast" or "Murmansk Oblast" or "Sakha" or "Arkhangelsk Oblast" or "Irkutsk Oblast" or "Khabarovsk Krai" or "Komi Republic" or "Krasnoyarsk Krai" or "Republic of Karelia" or "Sakhalin Oblast" or "Tuva" or "Tyumen Oblast" or "Polar Urals" or "Yamalia") or ("Svalbard" or "Spitsbergen" or "Bjørnøya" or "Jan Mayen" or "Finnmark" or "Troms" or "Lapland" or "Lappi" or "Grímsey" or "Lappland" or "Norrbotten" or "Västerbotten" or "North West Europe" or "Northwest Europe" or "north* Europe" or "Scandes" or "Kola Peninsula") or ("beringia" or "beringian") or "global data*").bt. or ("arctic" or "high-latitude" or "oro-arctic" or "North Polar region" or "subarctic" or "northern environ*" or ("Canada" or "Canadian" or "Russia" or "Russian Federation" or "USSR" or "RSFSR" or "Russian Federation" or "Russian" or "Norway" or "Norwegian" or "Finland" or "Finnish" or "Sweden" or "Iceland" or "Icelandic" or "Greenland" or "Greenlandic" or "Faroe") or ("tundra" or "permafrost" or "mammoth steppe") or ("Yukon Territory" or "Northwest Territories" or "Nunavut" or "Baffin Island" or "Belcher Islands" or "Ellesmere Island" or "Southampton Island" or "Mackenzie River" or "Great Slave Lake" or "Great Bear Lake" or "Yukon River" or "Alaska" or "Teshekpuk Lake" or "Queen Elizabeth Islands" or "Victoria Island" or "Banks Island" or "Wrangel Island" or "Seward peninsula" or "Hudson Bay") or ("Siberia" or "Siberian" or "Lake Baikal" or "Lena River" or "New Siberian Islands" or "Severnaya Zemlya" or "Franz Josef Land" or "Novaya Zemlya" or "Chukotka Autonomous Okrug" or "Kamchatka Krai" or "Magadan Oblast" or "Murmansk Oblast" or "Sakha" or "Arkhangelsk Oblast" or "Irkutsk Oblast" or "Khabarovsk Krai" or "Komi Republic" or "Krasnoyarsk Krai" or "Republic of Karelia" or "Sakhalin Oblast" or "Tuva" or "Tyumen Oblast" or "Polar Urals" or "Yamalia") or ("Svalbard" or "Spitsbergen" or "Bjørnøya" or "Jan Mayen" or "Finnmark" or "Troms" or "Lapland" or "Lappi" or "Grímsey" or "Lappland" or "Norrbotten" or "Västerbotten" or "North West Europe" or "Northwest Europe" or "north* Europe" or "Scandes" or "Kola Peninsula") or ("beringia" or "beringian") or "global data*").cw. or ("arctic" or "high-latitude" or "oro-arctic" or "North Polar region" or "subarctic" or "northern environ*" or ("Canada" or "Canadian" or "Russia" or "Russian Federation" or "USSR" or "RSFSR" or "Russian Federation" or "Russian" or "Norway" or "Norwegian" or "Finland" or "Finnish" or "Sweden" or "Iceland" or "Icelandic" or "Greenland" or "Greenlandic" or "Faroe") or ("tundra" or "permafrost" or "mammoth steppe") or ("Yukon Territory" or "Northwest Territories" or "Nunavut" or "Baffin Island" or "Belcher Islands" or "Ellesmere Island" or "Southampton Island" or "Mackenzie River" or "Great Slave Lake" or "Great Bear Lake" or "Yukon River" or "Alaska" or "Teshekpuk Lake" or "Queen Elizabeth Islands" or "Victoria Island" or "Banks Island" or "Wrangel Island" or "Seward peninsula" or "Hudson Bay") or ("Siberia" or "Siberian" or "Lake Baikal" or "Lena River" or "New Siberian Islands" or "Severnaya Zemlya" or "Franz Josef Land" or "Novaya Zemlya" or "Chukotka Autonomous Okrug" or "Kamchatka Krai" or "Magadan Oblast" or "Murmansk Oblast" or "Sakha" or "Arkhangelsk Oblast" or "Irkutsk Oblast" or "Khabarovsk Krai" or "Komi Republic" or "Krasnoyarsk Krai" or "Republic of Karelia" or "Sakhalin Oblast" or "Tuva" or "Tyumen Oblast" or "Polar Urals" or "Yamalia") or ("Svalbard" or "Spitsbergen" or "Bjørnøya" or "Jan Mayen" or "Finnmark" or "Troms" or "Lapland" or "Lappi" or "Grímsey" or "Lappland" or "Norrbotten" or "Västerbotten" or "North West Europe" or "Northwest Europe" or "north* Europe" or "Scandes" or "Kola Peninsula") or ("beringia" or "beringian") or "global data*").id. or ("arctic" or "high-latitude" or "oro-arctic" or "North Polar region" or "subarctic" or "northern environ*" or ("Canada" or "Canadian" or "Russia" or "Russian Federation" or "USSR" or "RSFSR" or "Russian Federation" or "Russian" or "Norway" or "Norwegian" or "Finland" or "Finnish" or "Sweden" or "Iceland" or "Icelandic" or "Greenland" or "Greenlandic" or "Faroe") or ("tundra" or "permafrost" or "mammoth steppe") or ("Yukon Territory" or "Northwest Territories" or "Nunavut" or "Baffin Island" or "Belcher Islands" or "Ellesmere Island" or "Southampton Island" or "Mackenzie River" or "Great Slave Lake" or "Great Bear Lake" or "Yukon River" or "Alaska" or "Teshekpuk Lake" or "Queen Elizabeth Islands" or "Victoria Island" or "Banks Island" or "Wrangel Island" or "Seward peninsula" or "Hudson Bay") or ("Siberia" or "Siberian" or "Lake Baikal" or "Lena River" or "New Siberian Islands" or "Severnaya Zemlya" or "Franz Josef Land" or "Novaya Zemlya" or "Chukotka Autonomous Okrug" or "Kamchatka Krai" or "Magadan Oblast" or "Murmansk Oblast" or "Sakha" or "Arkhangelsk Oblast" or "Irkutsk Oblast" or "Khabarovsk Krai" or "Komi Republic" or "Krasnoyarsk Krai" or "Republic of Karelia" or "Sakhalin Oblast" or "Tuva" or "Tyumen Oblast" or "Polar Urals" or "Yamalia") or ("Svalbard" or "Spitsbergen" or "Bjørnøya" or "Jan Mayen" or "Finnmark" or "Troms" or "Lapland" or "Lappi" or "Grímsey" or "Lappland" or "Norrbotten" or "Västerbotten" or "North West Europe" or "Northwest Europe" or "north* Europe" or "Scandes" or "Kola Peninsula") or ("beringia" or "beringian") or "global data*").ab.
2. ("reconstruct" or "reconstruction" or "reconstructed" or ("pal$eo*" or "micropal$entol*") or ("arch$eolog*" or "artefact") or ("history" or "historic site" or "historical record") or ("dendrochron*" or "dendroclim*" or "dendroeco*" or "growth ring" or "tree ring" or "wood ring") or ("radiocarbon" or "radio-carbon" or "AMS" or "accelerator mass spectrometry") or ("age determination" or "years before present" or "yr BP" or "calibration of age" or "age model" or "age-depth model") or ("1?,??? 14C yr B$P" or "1? ??? 14C Yr B$P" or "1?,??? yr B$P" or "1? ??? yr B$P" or "1?,??? cal yr B$P" or "1? ??? cal yr B$P" or "pre-1???" or "pre-20th century" or "1?th century" or "last ?,??? years" or "last 1?,??? years" or "14C dates" or "1? Cal Ka" or "one century" or "pre-industrial" or "preindustrial") or ("fossil" or "sub-fossil" or "macrofossil" or "megafossil" or "microfossil" or "subfossil") or "palynolog*" or ("prehistoric" or "prehistory" or "Iron Age" or "Bronze Age" or "Neolithic" or "Mesolithic" or "Late Upper Pal$eolithic" or "BC") or ("Holocene" or "MidHolocene" or "Mid-Holocene" or "late Pleistocene" or "end of the Pleistocene" or "Late Quaternary" or "Little Ice Age" or "Medieval Climate Anomoly" or "Younger Dryas" or "MIS 1" or "marine isotope stage 1") or ("centennial" or "centuries" or "millenial" or "millenia" or "submillenial" or "chronolog*" or "temporal trend") or ("preboreal chronozone" or "boreal chronozone" or "atlantic chronozone" or "subboreal chronozone") or ("sediment core" or "sedimentary sequence" or "lake sediment" or "transfer function" or "peat core" or "peat bog core" or "peat monolith") or ("ancient DNA" or "aDNA" or "sedDNA" or "sedimentary DNA") or ("postglacial" or "post-glacial" or "deglaciation" or "deglacial") or ("last appearance date" or "refugia") or ("multiproxy" or "proxy record" or "proxy archive" or "proxy" or "proxy study" or "proxy studies") or ("long-term ecolog*" or "long-term record")).ti. or ("reconstruct" or "reconstruction" or "reconstructed" or ("pal$eo*" or "micropal$entol*") or ("arch$eolog*" or "artefact") or ("history" or "historic site" or "historical record") or ("dendrochron*" or "dendroclim*" or "dendroeco*" or "growth ring" or "tree ring" or "wood ring") or ("radiocarbon" or "radio-carbon" or "AMS" or "accelerator mass spectrometry") or ("age determination" or "years before present" or "yr BP" or "calibration of age" or "age model" or "age-depth model") or ("1?,??? 14C yr B$P" or "1? ??? 14C Yr B$P" or "1?,??? yr B$P" or "1? ??? yr B$P" or "1?,??? cal yr B$P" or "1? ??? cal yr B$P" or "pre-1???" or "pre-20th century" or "1?th century" or "last ?,??? years" or "last 1?,??? years" or "14C dates" or "1? Cal Ka" or "one century" or "pre- industrial" or "preindustrial") or ("fossil" or "sub-fossil" or "macrofossil" or "megafossil" or "microfossil" or "subfossil") or "palynolog*" or ("prehistoric" or "prehistory" or "Iron Age" or "Bronze Age" or "Neolithic" or "Mesolithic" or "Late Upper Pal$eolithic" or "BC") or ("Holocene" or "MidHolocene" or "Mid- Holocene" or "late Pleistocene" or "end of the Pleistocene" or "Late Quaternary" or "Little Ice Age" or "Medieval Climate Anomoly" or "Younger Dryas" or "MIS 1" or "marine isotope stage 1") or ("centennial" or "centuries" or "millenial" or "millenia" or "submillenial" or "chronolog*" or "temporal trend") or ("preboreal chronozone" or "boreal chronozone" or "atlantic chronozone" or "subboreal chronozone") or ("sediment core" or "sedimentary sequence" or "lake sediment" or "transfer function" or "peat core" or "peat bog core" or "peat monolith") or ("ancient DNA" or "aDNA" or "sedDNA" or "sedimentary DNA") or ("postglacial" or "post-glacial" or "deglaciation" or "deglacial") or ("last appearance date" or "refugia") or ("multiproxy" or "proxy record" or "proxy archive" or "proxy" or "proxy study" or "proxy studies") or ("long-term ecolog*" or "long-term record")).ab. or ("reconstruct" or "reconstruction" or "reconstructed" or ("pal$eo*" or "micropal$entol*") or ("arch$eolog*" or "artefact") or ("history" or "historic site" or "historical record") or ("dendrochron*" or "dendroclim*" or "dendroeco*" or "growth ring" or "tree ring" or "wood ring") or ("radiocarbon" or "radio-carbon" or "AMS" or "accelerator mass spectrometry") or ("age determination" or "years before present" or "yr BP" or "calibration of age" or "age model" or "age-depth model") or ("1?,??? 14C yr B$P" or "1? ??? 14C Yr B$P" or "1?,??? yr B$P" or "1? ??? yr B$P" or "1?,??? cal yr B$P" or "1? ??? cal yr B$P" or "pre-1???" or "pre-20th century" or "1?th century" or "last ?,??? years" or "last 1?,??? years" or "14C dates" or "1? Cal Ka" or "one century" or "pre-industrial" or "preindustrial") or ("fossil" or "sub-fossil" or "macrofossil" or "megafossil" or "microfossil" or "subfossil") or "palynolog*" or ("prehistoric" or "prehistory" or "Iron Age" or "Bronze Age" or "Neolithic" or "Mesolithic" or "Late Upper Pal$eolithic" or "BC") or ("Holocene" or "MidHolocene" or "Mid-Holocene" or "late Pleistocene" or "end of the Pleistocene" or "Late Quaternary" or "Little Ice Age" or "Medieval Climate Anomoly" or "Younger Dryas" or "MIS 1" or "marine isotope stage 1") or ("centennial" or "centuries" or "millenial" or "millenia" or "submillenial" or "chronolog*" or "temporal trend") or ("preboreal chronozone" or "boreal chronozone" or "atlantic chronozone" or "subboreal chronozone") or ("sediment core" or "sedimentary sequence" or "lake sediment" or "transfer function" or "peat core" or "peat bog core" or "peat monolith") or ("ancient DNA" or "aDNA" or "sedDNA" or "sedimentary DNA") or ("postglacial" or "post-glacial" or "deglaciation" or "deglacial") or ("last appearance date" or "refugia") or ("multiproxy" or "proxy record" or "proxy archive" or "proxy" or "proxy study" or "proxy studies") or ("long-term ecolog*" or "long-term record")).bt. or ("reconstruct" or "reconstruction" or "reconstructed" or ("pal$eo*" or "micropal$entol*") or ("arch$eolog*" or "artefact") or ("history" or "historic site" or "historical record") or ("dendrochron*" or "dendroclim*" or "dendroeco*" or "growth ring" or "tree ring" or "wood ring") or ("radiocarbon" or "radio-carbon" or "AMS" or "accelerator mass spectrometry") or ("age determination" or "years before present" or "yr BP" or "calibration of age" or "age model" or "age-depth model") or ("1?,??? 14C yr B$P" or "1? ??? 14C Yr B$P" or "1?,??? yr B$P" or "1? ??? yr B$P" or "1?,??? cal yr B$P" or "1? ??? cal yr B$P" or "pre-1???" or "pre-20th century" or "1?th century" or "last ?,??? years" or "last 1?,??? years" or "14C dates" or "1? Cal Ka" or "one century" or "pre- industrial" or "preindustrial") or ("fossil" or "sub-fossil" or "macrofossil" or "megafossil" or "microfossil" or "subfossil") or "palynolog*" or ("prehistoric" or "prehistory" or "Iron Age" or "Bronze Age" or "Neolithic" or "Mesolithic" or "Late Upper Pal$eolithic" or "BC") or ("Holocene" or "MidHolocene" or "Mid- Holocene" or "late Pleistocene" or "end of the Pleistocene" or "Late Quaternary" or "Little Ice Age" or "Medieval Climate Anomoly" or "Younger Dryas" or "MIS 1" or "marine isotope stage 1") or ("centennial" or "centuries" or "millenial" or "millenia" or "submillenial" or "chronolog*" or "temporal trend") or ("preboreal chronozone" or "boreal chronozone" or "atlantic chronozone" or "subboreal chronozone") or ("sediment core" or "sedimentary sequence" or "lake sediment" or "transfer function" or "peat core" or "peat bog core" or "peat monolith") or ("ancient DNA" or "aDNA" or "sedDNA" or "sedimentary DNA") or ("postglacial" or "post-glacial" or "deglaciation" or "deglacial") or ("last appearance date" or "refugia") or ("multiproxy" or "proxy record" or "proxy archive" or "proxy" or "proxy study" or "proxy studies") or ("long-term ecolog*" or "long-term record")).cc. or ("reconstruct" or "reconstruction" or "reconstructed" or ("pal$eo*" or "micropal$entol*") or ("arch$eolog*" or "artefact") or ("history" or "historic site" or "historical record") or ("dendrochron*" or "dendroclim*" or "dendroeco*" or "growth ring" or "tree ring" or "wood ring") or ("radiocarbon" or "radio-carbon" or "AMS" or "accelerator mass spectrometry") or ("age determination" or "years before present" or "yr BP" or "calibration of age" or "age model" or "age-depth model") or ("1?,??? 14C yr B$P" or "1? ??? 14C Yr B$P" or "1?,??? yr B$P" or "1? ??? yr B$P" or "1?,??? cal yr B$P" or "1? ??? cal yr B$P" or "pre-1???" or "pre-20th century" or "1?th century" or "last ?,??? years" or "last 1?,??? years" or "14C dates" or "1? Cal Ka" or "one century" or "pre-industrial" or "preindustrial") or ("fossil" or "sub-fossil" or "macrofossil" or "megafossil" or "microfossil" or "subfossil") or "palynolog*" or ("prehistoric" or "prehistory" or "Iron Age" or "Bronze Age" or "Neolithic" or "Mesolithic" or "Late Upper Pal$eolithic" or "BC") or ("Holocene" or "MidHolocene" or "Mid-Holocene" or "late Pleistocene" or "end of the Pleistocene" or "Late Quaternary" or "Little Ice Age" or "Medieval Climate Anomoly" or "Younger Dryas" or "MIS 1" or "marine isotope stage 1") or ("centennial" or "centuries" or "millenial" or "millenia" or "submillenial" or "chronolog*" or "temporal trend") or ("preboreal chronozone" or "boreal chronozone" or "atlantic chronozone" or "subboreal chronozone") or ("sediment core" or "sedimentary sequence" or "lake sediment" or "transfer function" or "peat core" or "peat bog core" or "peat monolith") or ("ancient DNA" or "aDNA" or "sedDNA" or "sedimentary DNA") or ("postglacial" or "post-glacial" or "deglaciation" or "deglacial") or ("last appearance date" or "refugia") or ("multiproxy" or "proxy record" or "proxy archive" or "proxy" or "proxy study" or "proxy studies") or ("long-term ecolog*" or "long-term record")).id. or ("reconstruct" or "reconstruction" or "reconstructed" or ("pal$eo*" or "micropal$entol*") or ("arch$eolog*" or "artefact") or ("history" or "historic site" or "historical record") or ("dendrochron*" or "dendroclim*" or "dendroeco*" or "growth ring" or "tree ring" or "wood ring") or ("radiocarbon" or "radio-carbon" or "AMS" or "accelerator mass spectrometry") or ("age determination" or "years before present" or "yr BP" or "calibration of age" or "age model" or "age-depth model") or ("1?,??? 14C yr B$P" or "1? ??? 14C Yr B$P" or "1?,??? yr B$P" or "1? ??? yr B$P" or "1?,??? cal yr B$P" or "1? ??? cal yr B$P" or "pre-1???" or "pre-20th century" or "1?th century" or "last ?,??? years" or "last 1?,??? years" or "14C dates" or "1? Cal Ka" or "one century" or "pre- industrial" or "preindustrial") or ("fossil" or "sub-fossil" or "macrofossil" or "megafossil" or "microfossil" or "subfossil") or "palynolog*" or ("prehistoric" or "prehistory" or "Iron Age" or "Bronze Age" or "Neolithic" or "Mesolithic" or "Late Upper Pal$eolithic" or "BC") or ("Holocene" or "MidHolocene" or "Mid- Holocene" or "late Pleistocene" or "end of the Pleistocene" or "Late Quaternary" or "Little Ice Age" or "Medieval Climate Anomoly" or "Younger Dryas" or "MIS 1" or "marine isotope stage 1") or ("centennial" or "centuries" or "millenial" or "millenia" or "submillenial" or "chronolog*" or "temporal trend") or ("preboreal chronozone" or "boreal chronozone" or "atlantic chronozone" or "subboreal chronozone") or ("sediment core" or "sedimentary sequence" or "lake sediment" or "transfer function" or "peat core" or "peat bog core" or "peat monolith") or ("ancient DNA" or "aDNA" or "sedDNA" or "sedimentary DNA") or ("postglacial" or "post-glacial" or "deglaciation" or "deglacial") or ("last appearance date" or "refugia") or ("multiproxy" or "proxy record" or "proxy archive" or "proxy" or "proxy study" or "proxy studies") or ("long-term ecolog*" or "long-term record")).ot.
3. ("plant?" or "flora" or "vegetation" or "Plantae" or "vegetational" or "pal?eovegetation" or "floristic" or ("bryophyte" or "Bryophyta" or "liverwort?" or "moss" or "mosses") or ("shrub?" or "woody plant" or "vascular plant" or "fern?" or "pteridophyt?" or "spermatopht?" or "xerophyte" or "tree species" or "coniferous" or "conifer" or "deciduous" or "herb" or "forb?") or ("forest species" or "treeline species" or "tree-line species" or "larch" or "Larix" or "spruce" or "picea" or "western hemlock" or "Tsuga" or "Sitka" or "alnus" or "alder" or "cottonwood" or "Populus" or "poplar" or "Pinus" or "pine" or "Betula" or "birch" or "willow" or "salix") or ("lichen?" or "sporomiella" or "dung fungal spore" or "fungi" or "fungal") or ("diatom" or "Bacillariophyceae") or ("Mollusca" or "mollusk" or "mollusc" or "Gastropoda" or "gastropod" or "Bivalvia" or "bivalve") or ("mammal" or "mammalian" or "mammoth" or "reindeer" or "caribou" or "Rangifer tarandus" or "polar bear" or "Mammuthus primigenius" or "steppe bison" or "Bison priscus" or "muskox" or "Ovibos moschatus" or "moose" or "Alcos alces" or "horse" or "Equus caballus" or "wolf" or "wolves" or "Canis lupus" or "dog" or "squirrel" or "rodent" or "Rodentia" or "Urocitellus" or "arctic fox" or "vulpes lagopus" or "alopex lagopus" or "reptile" or "bird?" or "Aves") or ("ostracod" or "ostracode" or "ostracoda") or ("coleoptera" or "beetle") or ("chironomid" or "Chironomidae") or ("insect" or "macroinvertebrate" or "macro-invertebrate") or ("amphibian" or "Amphibia" or "aquatic animal" or "algae" or "algal") or ("animal" or "fauna" or "faunal" or "megafauna" or "megaherbivore" or "vertebrata" or "vertebrate") or ("pollen" or "palynology" or "macrofossil" or "pal$eobotanical" or "leaf wax" or "palynomorph") or "organic biomarker" or ("ring width" or "wood ring" or "tree ring" or "stem sample" or "driftwood") or ("terrestrial proxies" or "terrestrial proxy")).ti. or ("plant?" or "flora" or "vegetation" or "Plantae" or "vegetational" or "pal?eovegetation" or "floristic" or ("bryophyte" or "Bryophyta" or "liverwort?" or "moss" or "mosses") or ("shrub?" or "woody plant" or "vascular plant" or "fern?" or "pteridophyt?" or "spermatopht?" or "xerophyte" or "tree species" or "coniferous" or "conifer" or "deciduous" or "herb" or "forb?") or ("forest species" or "treeline species" or "tree-line species" or "larch" or "Larix" or "spruce" or "picea" or "western hemlock" or "Tsuga" or "Sitka" or "alnus" or "alder" or "cottonwood" or "Populus" or "poplar" or "Pinus" or "pine" or "Betula" or "birch" or "willow" or "salix") or ("lichen?" or "sporomiella" or "dung fungal spore" or "fungi" or "fungal") or ("diatom" or "Bacillariophyceae") or ("Mollusca" or "mollusk" or "mollusc" or "Gastropoda" or "gastropod" or "Bivalvia" or "bivalve") or ("mammal" or "mammalian" or "mammoth" or "reindeer" or "caribou" or "Rangifer tarandus" or "polar bear" or "Mammuthus primigenius" or "steppe bison" or "Bison priscus" or "muskox" or "Ovibos moschatus" or "moose" or "Alcos alces" or "horse" or "Equus caballus" or "wolf" or "wolves" or "Canis lupus" or "dog" or "squirrel" or "rodent" or "Rodentia" or "Urocitellus" or "arctic fox" or "vulpes lagopus" or "alopex lagopus" or "reptile" or "bird?" or "Aves") or ("ostracod" or "ostracode" or "ostracoda") or ("coleoptera" or "beetle") or ("chironomid" or "Chironomidae") or ("insect" or "macroinvertebrate" or "macro-invertebrate") or ("amphibian" or "Amphibia" or "aquatic animal" or "algae" or "algal") or ("animal" or "fauna" or "faunal" or "megafauna" or "megaherbivore" or "vertebrata" or "vertebrate") or ("pollen" or "palynology" or "macrofossil" or "pal$eobotanical" or "leaf wax" or "palynomorph") or "organic biomarker" or ("ring width" or "wood ring" or "tree ring" or "stem sample" or "driftwood") or ("terrestrial proxies" or "terrestrial proxy")).ab. or ("plant?" or "flora" or "vegetation" or "Plantae" or "vegetational" or "pal?eovegetation" or "floristic" or ("bryophyte" or "Bryophyta" or "liverwort?" or "moss" or "mosses") or ("shrub?" or "woody plant" or "vascular plant" or "fern?" or "pteridophyt?" or "spermatopht?" or "xerophyte" or "tree species" or "coniferous" or "conifer" or "deciduous" or "herb" or "forb?") or ("forest species" or "treeline species" or "tree-line species" or "larch" or "Larix" or "spruce" or "picea" or "western hemlock" or "Tsuga" or "Sitka" or "alnus" or "alder" or "cottonwood" or "Populus" or "poplar" or "Pinus" or "pine" or "Betula" or "birch" or "willow" or "salix") or ("lichen?" or "sporomiella" or "dung fungal spore" or "fungi" or "fungal") or ("diatom" or "Bacillariophyceae") or ("Mollusca" or "mollusk" or "mollusc" or "Gastropoda" or "gastropod" or "Bivalvia" or "bivalve") or ("mammal" or "mammalian" or "mammoth" or "reindeer" or "caribou" or "Rangifer tarandus" or "polar bear" or "Mammuthus primigenius" or "steppe bison" or "Bison priscus" or "muskox" or "Ovibos moschatus" or "moose" or "Alcos alces" or "horse" or "Equus caballus" or "wolf" or "wolves" or "Canis lupus" or "dog" or "squirrel" or "rodent" or "Rodentia" or "Urocitellus" or "arctic fox" or "vulpes lagopus" or "alopex lagopus" or "reptile" or "bird?" or "Aves") or ("ostracod" or "ostracode" or "ostracoda") or ("coleoptera" or "beetle") or ("chironomid" or "Chironomidae") or ("insect" or "macroinvertebrate" or "macro-invertebrate") or ("amphibian" or "Amphibia" or "aquatic animal" or "algae" or "algal") or ("animal" or "fauna" or "faunal" or "megafauna" or "megaherbivore" or "vertebrata" or "vertebrate") or ("pollen" or "palynology" or "macrofossil" or "pal$eobotanical" or "leaf wax" or "palynomorph") or "organic biomarker" or ("ring width" or "wood ring" or "tree ring" or "stem sample" or "driftwood") or ("terrestrial proxies" or "terrestrial proxy")).bt. or ("plant?" or "flora" or "vegetation" or "Plantae" or "vegetational" or "pal?eovegetation" or "floristic" or ("bryophyte" or "Bryophyta" or "liverwort?" or "moss" or "mosses") or ("shrub?" or "woody plant" or "vascular plant" or "fern?" or "pteridophyt?" or "spermatopht?" or "xerophyte" or "tree species" or "coniferous" or "conifer" or "deciduous" or "herb" or "forb?") or ("forest species" or "treeline species" or "tree-line species" or "larch" or "Larix" or "spruce" or "picea" or "western hemlock" or "Tsuga" or "Sitka" or "alnus" or "alder" or "cottonwood" or "Populus" or "poplar" or "Pinus" or "pine" or "Betula" or "birch" or "willow" or "salix") or ("lichen?" or "sporomiella" or "dung fungal spore" or "fungi" or "fungal") or ("diatom" or "Bacillariophyceae") or ("Mollusca" or "mollusk" or "mollusc" or "Gastropoda" or "gastropod" or "Bivalvia" or "bivalve") or ("mammal" or "mammalian" or "mammoth" or "reindeer" or "caribou" or "Rangifer tarandus" or "polar bear" or "Mammuthus primigenius" or "steppe bison" or "Bison priscus" or "muskox" or "Ovibos moschatus" or "moose" or "Alcos alces" or "horse" or "Equus caballus" or "wolf" or "wolves" or "Canis lupus" or "dog" or "squirrel" or "rodent" or "Rodentia" or "Urocitellus" or "arctic fox" or "vulpes lagopus" or "alopex lagopus" or "reptile" or "bird?" or "Aves") or ("ostracod" or "ostracode" or "ostracoda") or ("coleoptera" or "beetle") or ("chironomid" or "Chironomidae") or ("insect" or "macroinvertebrate" or "macro-invertebrate") or ("amphibian" or "Amphibia" or "aquatic animal" or "algae" or "algal") or ("animal" or "fauna" or "faunal" or "megafauna" or "megaherbivore" or "vertebrata" or "vertebrate") or ("pollen" or "palynology" or "macrofossil" or "pal$eobotanical" or "leaf wax" or "palynomorph") or "organic biomarker" or ("ring width" or "wood ring" or "tree ring" or "stem sample" or "driftwood") or ("terrestrial proxies" or "terrestrial proxy")).cc. or ("plant?" or "flora" or "vegetation" or "Plantae" or "vegetational" or "pal?eovegetation" or "floristic" or ("bryophyte" or "Bryophyta" or "liverwort?" or "moss" or "mosses") or ("shrub?" or "woody plant" or "vascular plant" or "fern?" or "pteridophyt?" or "spermatopht?" or "xerophyte" or "tree species" or "coniferous" or "conifer" or "deciduous" or "herb" or "forb?") or ("forest species" or "treeline species" or "tree-line species" or "larch" or "Larix" or "spruce" or "picea" or "western hemlock" or "Tsuga" or "Sitka" or "alnus" or "alder" or "cottonwood" or "Populus" or "poplar" or "Pinus" or "pine" or "Betula" or "birch" or "willow" or "salix") or ("lichen?" or "sporomiella" or "dung fungal spore" or "fungi" or "fungal") or ("diatom" or "Bacillariophyceae") or ("Mollusca" or "mollusk" or "mollusc" or "Gastropoda" or "gastropod" or "Bivalvia" or "bivalve") or ("mammal" or "mammalian" or "mammoth" or "reindeer" or "caribou" or "Rangifer tarandus" or "polar bear" or "Mammuthus primigenius" or "steppe bison" or "Bison priscus" or "muskox" or "Ovibos moschatus" or "moose" or "Alcos alces" or "horse" or "Equus caballus" or "wolf" or "wolves" or "Canis lupus" or "dog" or "squirrel" or "rodent" or "Rodentia" or "Urocitellus" or "arctic fox" or "vulpes lagopus" or "alopex lagopus" or "reptile" or "bird?" or "Aves") or ("ostracod" or "ostracode" or "ostracoda") or ("coleoptera" or "beetle") or ("chironomid" or "Chironomidae") or ("insect" or "macroinvertebrate" or "macro-invertebrate") or ("amphibian" or "Amphibia" or "aquatic animal" or "algae" or "algal") or ("animal" or "fauna" or "faunal" or "megafauna" or "megaherbivore" or "vertebrata" or "vertebrate") or ("pollen" or "palynology" or "macrofossil" or "pal$eobotanical" or "leaf wax" or "palynomorph") or "organic biomarker" or ("ring width" or "wood ring" or "tree ring" or "stem sample" or "driftwood") or ("terrestrial proxies" or "terrestrial proxy")).id. or ("plant?" or "flora" or "vegetation" or "Plantae" or "vegetational" or "pal?eovegetation" or "floristic" or ("bryophyte" or "Bryophyta" or "liverwort?" or "moss" or "mosses") or ("shrub?" or "woody plant" or "vascular plant" or "fern?" or "pteridophyt?" or "spermatopht?" or "xerophyte" or "tree species" or "coniferous" or "conifer" or "deciduous" or "herb" or "forb?") or ("forest species" or "treeline species" or "tree-line species" or "larch" or "Larix" or "spruce" or "picea" or "western hemlock" or "Tsuga" or "Sitka" or "alnus" or "alder" or "cottonwood" or "Populus" or "poplar" or "Pinus" or "pine" or "Betula" or "birch" or "willow" or "salix") or ("lichen?" or "sporomiella" or "dung fungal spore" or "fungi" or "fungal") or ("diatom" or "Bacillariophyceae") or ("Mollusca" or "mollusk" or "mollusc" or "Gastropoda" or "gastropod" or "Bivalvia" or "bivalve") or ("mammal" or "mammalian" or "mammoth" or "reindeer" or "caribou" or "Rangifer tarandus" or "polar bear" or "Mammuthus primigenius" or "steppe bison" or "Bison priscus" or "muskox" or "Ovibos moschatus" or "moose" or "Alcos alces" or "horse" or "Equus caballus" or "wolf" or "wolves" or "Canis lupus" or "dog" or "squirrel" or "rodent" or "Rodentia" or "Urocitellus" or "arctic fox" or "vulpes lagopus" or "alopex lagopus" or "reptile" or "bird?" or "Aves") or ("ostracod" or "ostracode" or "ostracoda") or ("coleoptera" or "beetle") or ("chironomid" or "Chironomidae") or ("insect" or "macroinvertebrate" or "macro-invertebrate") or ("amphibian" or "Amphibia" or "aquatic animal" or "algae" or "algal") or ("animal" or "fauna" or "faunal" or "megafauna" or "megaherbivore" or "vertebrata" or "vertebrate") or ("pollen" or "palynology" or "macrofossil" or "pal$eobotanical" or "leaf wax" or "palynomorph") or "organic biomarker" or ("ring width" or "wood ring" or "tree ring" or "stem sample" or "driftwood") or ("terrestrial proxies" or "terrestrial proxy")).od.
4. ("biodiversity" or ("diversity" or "evenness") or ("richness" or "number of species") or ("composition" or "assemblage" or "pal$eocommunit*" or "compositional shift" or "species dominance") or ("presence" or "present" or "absence" or "absent" or "occurrence" or "occurred") or ("stability" or "persistence" or "persisted" or "disappeared" or "extinction") or "abundance" or ("succession" or "vegetation development" or "plant cover" or "zonation pattern") or ("distribution" or "distributed" or "distributional") or ("coloni?ed" or "coloni?ation" or "established" or "spread to") or ("northern limit" or "northern treeline" or "ecotone" or "habitat diversity") or ("warming" or "cooling" or "temperature" or "temperatures" or "warm period" or "cold period" or "summer warmth") or ("hydroclimate" or "precipitation") or ("pal$eoclimat*" or "past climate" or "climate of the past") or "lake level" or ("vegetation productivity" or "plant productivity" or "radial growth" or "annual growth" or "growth rate" or "fuel load") or ("climate variable" or "climate interpretation" or "proxy climate record") or ("snow cover" or "snow extent") or ("organic carbon release" or "permafrost carbon" or "soil carbon release") or ("sea ice extent" or "relative sea level" or "sea level rise") or ("hunting" or "husbandry" or "domestication" or "construction activity" or "settlement history")).ti. or ("biodiversity" or ("diversity" or "evenness") or ("richness" or "number of species") or ("composition" or "assemblage" or "pal$eocommunit*" or "compositional shift" or "species dominance") or ("presence" or "present" or "absence" or "absent" or "occurrence" or "occurred") or ("stability" or "persistence" or "persisted" or "disappeared" or "extinction") or "abundance" or ("succession" or "vegetation development" or "plant cover" or "zonation pattern") or ("distribution" or "distributed" or "distributional") or ("coloni?ed" or "coloni?ation" or "established" or "spread to") or ("northern limit" or "northern treeline" or "ecotone" or "habitat diversity") or ("warming" or "cooling" or "temperature" or "temperatures" or "warm period" or "cold period" or "summer warmth") or ("hydroclimate" or "precipitation") or ("pal$eoclimat*" or "past climate" or "climate of the past") or "lake level" or ("vegetation productivity" or "plant productivity" or "radial growth" or "annual growth" or "growth rate" or "fuel load") or ("climate variable" or "climate interpretation" or "proxy climate record") or ("snow cover" or "snow extent") or ("organic carbon release" or "permafrost carbon" or "soil carbon release") or ("sea ice extent" or "relative sea level" or "sea level rise") or ("hunting" or "husbandry" or "domestication" or "construction activity" or "settlement history")).ab. or ("biodiversity" or ("diversity" or "evenness") or ("richness" or "number of species") or ("composition" or "assemblage" or "pal$eocommunit*" or "compositional shift" or "species dominance") or ("presence" or "present" or "absence" or "absent" or "occurrence" or "occurred") or ("stability" or "persistence" or "persisted" or "disappeared" or "extinction") or "abundance" or ("succession" or "vegetation development" or "plant cover" or "zonation pattern") or ("distribution" or "distributed" or "distributional") or ("coloni?ed" or "coloni?ation" or "established" or "spread to") or ("northern limit" or "northern treeline" or "ecotone" or "habitat diversity") or ("warming" or "cooling" or "temperature" or "temperatures" or "warm period" or "cold period" or "summer warmth") or ("hydroclimate" or "precipitation") or ("pal$eoclimat*" or "past climate" or "climate of the past") or "lake level" or ("vegetation productivity" or "plant productivity" or "radial growth" or "annual growth" or "growth rate" or "fuel load") or ("climate variable" or "climate interpretation" or "proxy climate record") or ("snow cover" or "snow extent") or ("organic carbon release" or "permafrost carbon" or "soil carbon release") or ("sea ice extent" or "relative sea level" or "sea level rise") or ("hunting" or "husbandry" or "domestication" or "construction activity" or "settlement history")).br.

## BIOSIS Archive (pre-1968) via Ovid

Searches 1 to 4 combined using AND:

1. ("arctic" or "high-latitude" or "oro-arctic" or "North Polar region" or "subarctic" or "northern environ*" or ("Canada" or "Canadian" or "Russia" or "Russian Federation" or "USSR" or "RSFSR" or "Russian Federation" or "Russian" or "Norway" or "Norwegian" or "Finland" or "Finnish" or "Sweden" or "Iceland" or "Icelandic" or "Greenland" or "Greenlandic" or "Faroe") or ("tundra" or "permafrost" or "mammoth steppe") or ("Yukon Territory" or "Northwest Territories" or "Nunavut" or "Baffin Island" or "Belcher Islands" or "Ellesmere Island" or "Southampton Island" or "Mackenzie River" or "Great Slave Lake" or "Great Bear Lake" or "Yukon River" or "Alaska" or "Teshekpuk Lake" or "Queen Elizabeth Islands" or "Victoria Island" or "Banks Island" or "Wrangel Island" or "Seward peninsula" or "Hudson Bay") or ("Siberia" or "Siberian" or "Lake Baikal" or "Lena River" or "New Siberian Islands" or "Severnaya Zemlya" or "Franz Josef Land" or "Novaya Zemlya" or "Chukotka Autonomous Okrug" or "Kamchatka Krai" or "Magadan Oblast" or "Murmansk Oblast" or "Sakha" or "Arkhangelsk Oblast" or "Irkutsk Oblast" or "Khabarovsk Krai" or "Komi Republic" or "Krasnoyarsk Krai" or "Republic of Karelia" or "Sakhalin Oblast" or "Tuva" or "Tyumen Oblast" or "Polar Urals" or "Yamalia") or ("Svalbard" or "Spitsbergen" or "Bjørnøya" or "Jan Mayen" or "Finnmark" or "Troms" or "Lapland" or "Lappi" or "Grímsey" or "Lappland" or "Norrbotten" or "Västerbotten" or "North West Europe" or "Northwest Europe" or "north* Europe" or "Scandes" or "Kola Peninsula") or ("beringia" or "beringian") or "global data*").ti. or ("arctic" or "high-latitude" or "oro-arctic" or "North Polar region" or "subarctic" or "northern environ*" or ("Canada" or "Canadian" or "Russia" or "Russian Federation" or "USSR" or "RSFSR" or "Russian Federation" or "Russian" or "Norway" or "Norwegian" or "Finland" or "Finnish" or "Sweden" or "Iceland" or "Icelandic" or "Greenland" or "Greenlandic" or "Faroe") or ("tundra" or "permafrost" or "mammoth steppe") or ("Yukon Territory" or "Northwest Territories" or "Nunavut" or "Baffin Island" or "Belcher Islands" or "Ellesmere Island" or "Southampton Island" or "Mackenzie River" or "Great Slave Lake" or "Great Bear Lake" or "Yukon River" or "Alaska" or "Teshekpuk Lake" or "Queen Elizabeth Islands" or "Victoria Island" or "Banks Island" or "Wrangel Island" or "Seward peninsula" or "Hudson Bay") or ("Siberia" or "Siberian" or "Lake Baikal" or "Lena River" or "New Siberian Islands" or "Severnaya Zemlya" or "Franz Josef Land" or "Novaya Zemlya" or "Chukotka Autonomous Okrug" or "Kamchatka Krai" or "Magadan Oblast" or "Murmansk Oblast" or "Sakha" or "Arkhangelsk Oblast" or "Irkutsk Oblast" or "Khabarovsk Krai" or "Komi Republic" or "Krasnoyarsk Krai" or "Republic of Karelia" or "Sakhalin Oblast" or "Tuva" or "Tyumen Oblast" or "Polar Urals" or "Yamalia") or ("Svalbard" or "Spitsbergen" or "Bjørnøya" or "Jan Mayen" or "Finnmark" or "Troms" or "Lapland" or "Lappi" or "Grímsey" or "Lappland" or "Norrbotten" or "Västerbotten" or "North West Europe" or "Northwest Europe" or "north* Europe" or "Scandes" or "Kola Peninsula") or ("beringia" or "beringian") or "global data*").ab. or ("arctic" or "high-latitude" or "oro-arctic" or "North Polar region" or "subarctic" or "northern environ*" or ("Canada" or "Canadian" or "Russia" or "Russian Federation" or "USSR" or "RSFSR" or "Russian Federation" or "Russian" or "Norway" or "Norwegian" or "Finland" or "Finnish" or "Sweden" or "Iceland" or "Icelandic" or "Greenland" or "Greenlandic" or "Faroe") or ("tundra" or "permafrost" or "mammoth steppe") or ("Yukon Territory" or "Northwest Territories" or "Nunavut" or "Baffin Island" or "Belcher Islands" or "Ellesmere Island" or "Southampton Island" or "Mackenzie River" or "Great Slave Lake" or "Great Bear Lake" or "Yukon River" or "Alaska" or "Teshekpuk Lake" or "Queen Elizabeth Islands" or "Victoria Island" or "Banks Island" or "Wrangel Island" or "Seward peninsula" or "Hudson Bay") or ("Siberia" or "Siberian" or "Lake Baikal" or "Lena River" or "New Siberian Islands" or "Severnaya Zemlya" or "Franz Josef Land" or "Novaya Zemlya" or "Chukotka Autonomous Okrug" or "Kamchatka Krai" or "Magadan Oblast" or "Murmansk Oblast" or "Sakha" or "Arkhangelsk Oblast" or "Irkutsk Oblast" or "Khabarovsk Krai" or "Komi Republic" or "Krasnoyarsk Krai" or "Republic of Karelia" or "Sakhalin Oblast" or "Tuva" or "Tyumen Oblast" or "Polar Urals" or "Yamalia") or ("Svalbard" or "Spitsbergen" or "Bjørnøya" or "Jan Mayen" or "Finnmark" or "Troms" or "Lapland" or "Lappi" or "Grímsey" or "Lappland" or "Norrbotten" or "Västerbotten" or "North West Europe" or "Northwest Europe" or "north* Europe" or "Scandes" or "Kola Peninsula") or ("beringia" or "beringian") or "global data*").bt. or ("arctic" or "high-latitude" or "oro-arctic" or "North Polar region" or "subarctic" or "northern environ*" or ("Canada" or "Canadian" or "Russia" or "Russian Federation" or "USSR" or "RSFSR" or "Russian Federation" or "Russian" or "Norway" or "Norwegian" or "Finland" or "Finnish" or "Sweden" or "Iceland" or "Icelandic" or "Greenland" or "Greenlandic" or "Faroe") or ("tundra" or "permafrost" or "mammoth steppe") or ("Yukon Territory" or "Northwest Territories" or "Nunavut" or "Baffin Island" or "Belcher Islands" or "Ellesmere Island" or "Southampton Island" or "Mackenzie River" or "Great Slave Lake" or "Great Bear Lake" or "Yukon River" or "Alaska" or "Teshekpuk Lake" or "Queen Elizabeth Islands" or "Victoria Island" or "Banks Island" or "Wrangel Island" or "Seward peninsula" or "Hudson Bay") or ("Siberia" or "Siberian" or "Lake Baikal" or "Lena River" or "New Siberian Islands" or "Severnaya Zemlya" or "Franz Josef Land" or "Novaya Zemlya" or "Chukotka Autonomous Okrug" or "Kamchatka Krai" or "Magadan Oblast" or "Murmansk Oblast" or "Sakha" or "Arkhangelsk Oblast" or "Irkutsk Oblast" or "Khabarovsk Krai" or "Komi Republic" or "Krasnoyarsk Krai" or "Republic of Karelia" or "Sakhalin Oblast" or "Tuva" or "Tyumen Oblast" or "Polar Urals" or "Yamalia") or ("Svalbard" or "Spitsbergen" or "Bjørnøya" or "Jan Mayen" or "Finnmark" or "Troms" or "Lapland" or "Lappi" or "Grímsey" or "Lappland" or "Norrbotten" or "Västerbotten" or "North West Europe" or "Northwest Europe" or "north* Europe" or "Scandes" or "Kola Peninsula") or ("beringia" or "beringian") or "global data*").bo. or ("arctic" or "high-latitude" or "oro-arctic" or "North Polar region" or "subarctic" or "northern environ*" or ("Canada" or "Canadian" or "Russia" or "Russian Federation" or "USSR" or "RSFSR" or "Russian Federation" or "Russian" or "Norway" or "Norwegian" or "Finland" or "Finnish" or "Sweden" or "Iceland" or "Icelandic" or "Greenland" or "Greenlandic" or "Faroe") or ("tundra" or "permafrost" or "mammoth steppe") or ("Yukon Territory" or "Northwest Territories" or "Nunavut" or "Baffin Island" or "Belcher Islands" or "Ellesmere Island" or "Southampton Island" or "Mackenzie River" or "Great Slave Lake" or "Great Bear Lake" or "Yukon River" or "Alaska" or "Teshekpuk Lake" or "Queen Elizabeth Islands" or "Victoria Island" or "Banks Island" or "Wrangel Island" or "Seward peninsula" or "Hudson Bay") or ("Siberia" or "Siberian" or "Lake Baikal" or "Lena River" or "New Siberian Islands" or "Severnaya Zemlya" or "Franz Josef Land" or "Novaya Zemlya" or "Chukotka Autonomous Okrug" or "Kamchatka Krai" or "Magadan Oblast" or "Murmansk Oblast" or "Sakha" or "Arkhangelsk Oblast" or "Irkutsk Oblast" or "Khabarovsk Krai" or "Komi Republic" or "Krasnoyarsk Krai" or "Republic of Karelia" or "Sakhalin Oblast" or "Tuva" or "Tyumen Oblast" or "Polar Urals" or "Yamalia") or ("Svalbard" or "Spitsbergen" or "Bjørnøya" or "Jan Mayen" or "Finnmark" or "Troms" or "Lapland" or "Lappi" or "Grímsey" or "Lappland" or "Norrbotten" or "Västerbotten" or "North West Europe" or "Northwest Europe" or "north* Europe" or "Scandes" or "Kola Peninsula") or ("beringia" or "beringian") or "global data*").hw.
2. ("reconstruct" or "reconstruction" or "reconstructed" or ("pal$eo*" or "micropal$entol*") or ("arch$eolog*" or "artefact") or ("history" or "historic site" or "historical record") or ("dendrochron*" or "dendroclim*" or "dendroeco*" or "growth ring" or "tree ring" or "wood ring") or ("radiocarbon" or "radio-carbon" or "AMS" or "accelerator mass spectrometry") or ("age determination" or "years before present" or "yr BP" or "calibration of age" or "age model" or "age-depth model") or ("1?,??? 14C yr B$P" or "1? ??? 14C Yr B$P" or "1?,??? yr B$P" or "1? ??? yr B$P" or "1?,??? cal yr B$P" or "1? ??? cal yr B$P" or "pre-1???" or "pre-20th century" or "1?th century" or "last ?,??? years" or "last 1?,??? years" or "14C dates" or "1? Cal Ka" or "one century" or "pre-industrial" or "preindustrial") or ("fossil" or "sub-fossil" or "macrofossil" or "megafossil" or "microfossil" or "subfossil") or "palynolog*" or ("prehistoric" or "prehistory" or "Iron Age" or "Bronze Age" or "Neolithic" or "Mesolithic" or "Late Upper Pal$eolithic" or "BC") or ("Holocene" or "MidHolocene" or "Mid-Holocene" or "late Pleistocene" or "end of the Pleistocene" or "Late Quaternary" or "Little Ice Age" or "Medieval Climate Anomoly" or "Younger Dryas" or "MIS 1" or "marine isotope stage 1") or ("centennial" or "centuries" or "millenial" or "millenia" or "submillenial" or "chronolog*" or "temporal trend") or ("preboreal chronozone" or "boreal chronozone" or "atlantic chronozone" or "subboreal chronozone") or ("sediment core" or "sedimentary sequence" or "lake sediment" or "transfer function" or "peat core" or "peat bog core" or "peat monolith") or ("ancient DNA" or "aDNA" or "sedDNA" or "sedimentary DNA") or ("postglacial" or "post-glacial" or "deglaciation" or "deglacial") or ("last appearance date" or "refugia") or ("multiproxy" or "proxy record" or "proxy archive" or "proxy" or "proxy study" or "proxy studies") or ("long-term ecolog*" or "long-term record")).ti. or ("reconstruct" or "reconstruction" or "reconstructed" or ("pal$eo*" or "micropal$entol*") or ("arch$eolog*" or "artefact") or ("history" or "historic site" or "historical record") or ("dendrochron*" or "dendroclim*" or "dendroeco*" or "growth ring" or "tree ring" or "wood ring") or ("radiocarbon" or "radio-carbon" or "AMS" or "accelerator mass spectrometry") or ("age determination" or "years before present" or "yr BP" or "calibration of age" or "age model" or "age-depth model") or ("1?,??? 14C yr B$P" or "1? ??? 14C Yr B$P" or "1?,??? yr B$P" or "1? ??? yr B$P" or "1?,??? cal yr B$P" or "1? ??? cal yr B$P" or "pre-1???" or "pre-20th century" or "1?th century" or "last ?,??? years" or "last 1?,??? years" or "14C dates" or "1? Cal Ka" or "one century" or "pre- industrial" or "preindustrial") or ("fossil" or "sub-fossil" or "macrofossil" or "megafossil" or "microfossil" or "subfossil") or "palynolog*" or ("prehistoric" or "prehistory" or "Iron Age" or "Bronze Age" or "Neolithic" or "Mesolithic" or "Late Upper Pal$eolithic" or "BC") or ("Holocene" or "MidHolocene" or "Mid- Holocene" or "late Pleistocene" or "end of the Pleistocene" or "Late Quaternary" or "Little Ice Age" or "Medieval Climate Anomoly" or "Younger Dryas" or "MIS 1" or "marine isotope stage 1") or ("centennial" or "centuries" or "millenial" or "millenia" or "submillenial" or "chronolog*" or "temporal trend") or ("preboreal chronozone" or "boreal chronozone" or "atlantic chronozone" or "subboreal chronozone") or ("sediment core" or "sedimentary sequence" or "lake sediment" or "transfer function" or "peat core" or "peat bog core" or "peat monolith") or ("ancient DNA" or "aDNA" or "sedDNA" or "sedimentary DNA") or ("postglacial" or "post-glacial" or "deglaciation" or "deglacial") or ("last appearance date" or "refugia") or ("multiproxy" or "proxy record" or "proxy archive" or "proxy" or "proxy study" or "proxy studies") or ("long-term ecolog*" or "long-term record")).ab. or ("reconstruct" or "reconstruction" or "reconstructed" or ("pal$eo*" or "micropal$entol*") or ("arch$eolog*" or "artefact") or ("history" or "historic site" or "historical record") or ("dendrochron*" or "dendroclim*" or "dendroeco*" or "growth ring" or "tree ring" or "wood ring") or ("radiocarbon" or "radio-carbon" or "AMS" or "accelerator mass spectrometry") or ("age determination" or "years before present" or "yr BP" or "calibration of age" or "age model" or "age-depth model") or ("1?,??? 14C yr B$P" or "1? ??? 14C Yr B$P" or "1?,??? yr B$P" or "1? ??? yr B$P" or "1?,??? cal yr B$P" or "1? ??? cal yr B$P" or "pre-1???" or "pre-20th century" or "1?th century" or "last ?,??? years" or "last 1?,??? years" or "14C dates" or "1? Cal Ka" or "one century" or "pre-industrial" or "preindustrial") or ("fossil" or "sub-fossil" or "macrofossil" or "megafossil" or "microfossil" or "subfossil") or "palynolog*" or ("prehistoric" or "prehistory" or "Iron Age" or "Bronze Age" or "Neolithic" or "Mesolithic" or "Late Upper Pal$eolithic" or "BC") or ("Holocene" or "MidHolocene" or "Mid-Holocene" or "late Pleistocene" or "end of the Pleistocene" or "Late Quaternary" or "Little Ice Age" or "Medieval Climate Anomoly" or "Younger Dryas" or "MIS 1" or "marine isotope stage 1") or ("centennial" or "centuries" or "millenial" or "millenia" or "submillenial" or "chronolog*" or "temporal trend") or ("preboreal chronozone" or "boreal chronozone" or "atlantic chronozone" or "subboreal chronozone") or ("sediment core" or "sedimentary sequence" or "lake sediment" or "transfer function" or "peat core" or "peat bog core" or "peat monolith") or ("ancient DNA" or "aDNA" or "sedDNA" or "sedimentary DNA") or ("postglacial" or "post-glacial" or "deglaciation" or "deglacial") or ("last appearance date" or "refugia") or ("multiproxy" or "proxy record" or "proxy archive" or "proxy" or "proxy study" or "proxy studies") or ("long-term ecolog*" or "long-term record")).bt. or ("reconstruct" or "reconstruction" or "reconstructed" or ("pal$eo*" or "micropal$entol*") or ("arch$eolog*" or "artefact") or ("history" or "historic site" or "historical record") or ("dendrochron*" or "dendroclim*" or "dendroeco*" or "growth ring" or "tree ring" or "wood ring") or ("radiocarbon" or "radio-carbon" or "AMS" or "accelerator mass spectrometry") or ("age determination" or "years before present" or "yr BP" or "calibration of age" or "age model" or "age-depth model") or ("1?,??? 14C yr B$P" or "1? ??? 14C Yr B$P" or "1?,??? yr B$P" or "1? ??? yr B$P" or "1?,??? cal yr B$P" or "1? ??? cal yr B$P" or "pre-1???" or "pre-20th century" or "1?th century" or "last ?,??? years" or "last 1?,??? years" or "14C dates" or "1? Cal Ka" or "one century" or "pre- industrial" or "preindustrial") or ("fossil" or "sub-fossil" or "macrofossil" or "megafossil" or "microfossil" or "subfossil") or "palynolog*" or ("prehistoric" or "prehistory" or "Iron Age" or "Bronze Age" or "Neolithic" or "Mesolithic" or "Late Upper Pal$eolithic" or "BC") or ("Holocene" or "MidHolocene" or "Mid- Holocene" or "late Pleistocene" or "end of the Pleistocene" or "Late Quaternary" or "Little Ice Age" or "Medieval Climate Anomoly" or "Younger Dryas" or "MIS 1" or "marine isotope stage 1") or ("centennial" or "centuries" or "millenial" or "millenia" or "submillenial" or "chronolog*" or "temporal trend") or ("preboreal chronozone" or "boreal chronozone" or "atlantic chronozone" or "subboreal chronozone") or ("sediment core" or "sedimentary sequence" or "lake sediment" or "transfer function" or "peat core" or "peat bog core" or "peat monolith") or ("ancient DNA" or "aDNA" or "sedDNA" or "sedimentary DNA") or ("postglacial" or "post-glacial" or "deglaciation" or "deglacial") or ("last appearance date" or "refugia") or ("multiproxy" or "proxy record" or "proxy archive" or "proxy" or "proxy study" or "proxy studies") or ("long-term ecolog*" or "long-term record")).bo. or ("reconstruct" or "reconstruction" or "reconstructed" or ("pal$eo*" or "micropal$entol*") or ("arch$eolog*" or "artefact") or ("history" or "historic site" or "historical record") or ("dendrochron*" or "dendroclim*" or "dendroeco*" or "growth ring" or "tree ring" or "wood ring") or ("radiocarbon" or "radio-carbon" or "AMS" or "accelerator mass spectrometry") or ("age determination" or "years before present" or "yr BP" or "calibration of age" or "age model" or "age-depth model") or ("1?,??? 14C yr B$P" or "1? ??? 14C Yr B$P" or "1?,??? yr B$P" or "1? ??? yr B$P" or "1?,??? cal yr B$P" or "1? ??? cal yr B$P" or "pre-1???" or "pre-20th century" or "1?th century" or "last ?,??? years" or "last 1?,??? years" or "14C dates" or "1? Cal Ka" or "one century" or "pre-industrial" or "preindustrial") or ("fossil" or "sub-fossil" or "macrofossil" or "megafossil" or "microfossil" or "subfossil") or "palynolog*" or ("prehistoric" or "prehistory" or "Iron Age" or "Bronze Age" or "Neolithic" or "Mesolithic" or "Late Upper Pal$eolithic" or "BC") or ("Holocene" or "MidHolocene" or "Mid-Holocene" or "late Pleistocene" or "end of the Pleistocene" or "Late Quaternary" or "Little Ice Age" or "Medieval Climate Anomoly" or "Younger Dryas" or "MIS 1" or "marine isotope stage 1") or ("centennial" or "centuries" or "millenial" or "millenia" or "submillenial" or "chronolog*" or "temporal trend") or ("preboreal chronozone" or "boreal chronozone" or "atlantic chronozone" or "subboreal chronozone") or ("sediment core" or "sedimentary sequence" or "lake sediment" or "transfer function" or "peat core" or "peat bog core" or "peat monolith") or ("ancient DNA" or "aDNA" or "sedDNA" or "sedimentary DNA") or ("postglacial" or "post-glacial" or "deglaciation" or "deglacial") or ("last appearance date" or "refugia") or ("multiproxy" or "proxy record" or "proxy archive" or "proxy" or "proxy study" or "proxy studies") or ("long-term ecolog*" or "long-term record")).hw.
3. ("plant?" or "flora" or "vegetation" or "Plantae" or "vegetational" or "pal?eovegetation" or "floristic" or ("bryophyte" or "Bryophyta" or "liverwort?" or "moss" or "mosses") or ("shrub?" or "woody plant" or "vascular plant" or "fern?" or "pteridophyt?" or "spermatopht?" or "xerophyte" or "tree species" or "coniferous" or "conifer" or "deciduous" or "herb" or "forb?") or ("forest species" or "treeline species" or "tree-line species" or "larch" or "Larix" or "spruce" or "picea" or "western hemlock" or "Tsuga" or "Sitka" or "alnus" or "alder" or "cottonwood" or "Populus" or "poplar" or "Pinus" or "pine" or "Betula" or "birch" or "willow" or "salix") or ("lichen?" or "sporomiella" or "dung fungal spore" or "fungi" or "fungal") or ("diatom" or "Bacillariophyceae") or ("Mollusca" or "mollusk" or "mollusc" or "Gastropoda" or "gastropod" or "Bivalvia" or "bivalve") or ("mammal" or "mammalian" or "mammoth" or "reindeer" or "caribou" or "Rangifer tarandus" or "polar bear" or "Mammuthus primigenius" or "steppe bison" or "Bison priscus" or "muskox" or "Ovibos moschatus" or "moose" or "Alcos alces" or "horse" or "Equus caballus" or "wolf" or "wolves" or "Canis lupus" or "dog" or "squirrel" or "rodent" or "Rodentia" or "Urocitellus" or "arctic fox" or "vulpes lagopus" or "alopex lagopus" or "reptile" or "bird?" or "Aves") or ("ostracod" or "ostracode" or "ostracoda") or ("coleoptera" or "beetle") or ("chironomid" or "Chironomidae") or ("insect" or "macroinvertebrate" or "macro-invertebrate") or ("amphibian" or "Amphibia" or "aquatic animal" or "algae" or "algal") or ("animal" or "fauna" or "faunal" or "megafauna" or "megaherbivore" or "vertebrata" or "vertebrate") or ("pollen" or "palynology" or "macrofossil" or "pal$eobotanical" or "leaf wax" or "palynomorph") or "organic biomarker" or ("ring width" or "wood ring" or "tree ring" or "stem sample" or "driftwood") or ("terrestrial proxies" or "terrestrial proxy")).ti. or ("plant?" or "flora" or "vegetation" or "Plantae" or "vegetational" or "pal?eovegetation" or "floristic" or ("bryophyte" or "Bryophyta" or "liverwort?" or "moss" or "mosses") or ("shrub?" or "woody plant" or "vascular plant" or "fern?" or "pteridophyt?" or "spermatopht?" or "xerophyte" or "tree species" or "coniferous" or "conifer" or "deciduous" or "herb" or "forb?") or ("forest species" or "treeline species" or "tree-line species" or "larch" or "Larix" or "spruce" or "picea" or "western hemlock" or "Tsuga" or "Sitka" or "alnus" or "alder" or "cottonwood" or "Populus" or "poplar" or "Pinus" or "pine" or "Betula" or "birch" or "willow" or "salix") or ("lichen?" or "sporomiella" or "dung fungal spore" or "fungi" or "fungal") or ("diatom" or "Bacillariophyceae") or ("Mollusca" or "mollusk" or "mollusc" or "Gastropoda" or "gastropod" or "Bivalvia" or "bivalve") or ("mammal" or "mammalian" or "mammoth" or "reindeer" or "caribou" or "Rangifer tarandus" or "polar bear" or "Mammuthus primigenius" or "steppe bison" or "Bison priscus" or "muskox" or "Ovibos moschatus" or "moose" or "Alcos alces" or "horse" or "Equus caballus" or "wolf" or "wolves" or "Canis lupus" or "dog" or "squirrel" or "rodent" or "Rodentia" or "Urocitellus" or "arctic fox" or "vulpes lagopus" or "alopex lagopus" or "reptile" or "bird?" or "Aves") or ("ostracod" or "ostracode" or "ostracoda") or ("coleoptera" or "beetle") or ("chironomid" or "Chironomidae") or ("insect" or "macroinvertebrate" or "macro-invertebrate") or ("amphibian" or "Amphibia" or "aquatic animal" or "algae" or "algal") or ("animal" or "fauna" or "faunal" or "megafauna" or "megaherbivore" or "vertebrata" or "vertebrate") or ("pollen" or "palynology" or "macrofossil" or "pal$eobotanical" or "leaf wax" or "palynomorph") or "organic biomarker" or ("ring width" or "wood ring" or "tree ring" or "stem sample" or "driftwood") or ("terrestrial proxies" or "terrestrial proxy")).ab. or ("plant?" or "flora" or "vegetation" or "Plantae" or "vegetational" or "pal?eovegetation" or "floristic" or ("bryophyte" or "Bryophyta" or "liverwort?" or "moss" or "mosses") or ("shrub?" or "woody plant" or "vascular plant" or "fern?" or "pteridophyt?" or "spermatopht?" or "xerophyte" or "tree species" or "coniferous" or "conifer" or "deciduous" or "herb" or "forb?") or ("forest species" or "treeline species" or "tree-line species" or "larch" or "Larix" or "spruce" or "picea" or "western hemlock" or "Tsuga" or "Sitka" or "alnus" or "alder" or "cottonwood" or "Populus" or "poplar" or "Pinus" or "pine" or "Betula" or "birch" or "willow" or "salix") or ("lichen?" or "sporomiella" or "dung fungal spore" or "fungi" or "fungal") or ("diatom" or "Bacillariophyceae") or ("Mollusca" or "mollusk" or "mollusc" or "Gastropoda" or "gastropod" or "Bivalvia" or "bivalve") or ("mammal" or "mammalian" or "mammoth" or "reindeer" or "caribou" or "Rangifer tarandus" or "polar bear" or "Mammuthus primigenius" or "steppe bison" or "Bison priscus" or "muskox" or "Ovibos moschatus" or "moose" or "Alcos alces" or "horse" or "Equus caballus" or "wolf" or "wolves" or "Canis lupus" or "dog" or "squirrel" or "rodent" or "Rodentia" or "Urocitellus" or "arctic fox" or "vulpes lagopus" or "alopex lagopus" or "reptile" or "bird?" or "Aves") or ("ostracod" or "ostracode" or "ostracoda") or ("coleoptera" or "beetle") or ("chironomid" or "Chironomidae") or ("insect" or "macroinvertebrate" or "macro-invertebrate") or ("amphibian" or "Amphibia" or "aquatic animal" or "algae" or "algal") or ("animal" or "fauna" or "faunal" or "megafauna" or "megaherbivore" or "vertebrata" or "vertebrate") or ("pollen" or "palynology" or "macrofossil" or "pal$eobotanical" or "leaf wax" or "palynomorph") or "organic biomarker" or ("ring width" or "wood ring" or "tree ring" or "stem sample" or "driftwood") or ("terrestrial proxies" or "terrestrial proxy")).bt. or ("plant?" or "flora" or "vegetation" or "Plantae" or "vegetational" or "pal?eovegetation" or "floristic" or ("bryophyte" or "Bryophyta" or "liverwort?" or "moss" or "mosses") or ("shrub?" or "woody plant" or "vascular plant" or "fern?" or "pteridophyt?" or "spermatopht?" or "xerophyte" or "tree species" or "coniferous" or "conifer" or "deciduous" or "herb" or "forb?") or ("forest species" or "treeline species" or "tree-line species" or "larch" or "Larix" or "spruce" or "picea" or "western hemlock" or "Tsuga" or "Sitka" or "alnus" or "alder" or "cottonwood" or "Populus" or "poplar" or "Pinus" or "pine" or "Betula" or "birch" or "willow" or "salix") or ("lichen?" or "sporomiella" or "dung fungal spore" or "fungi" or "fungal") or ("diatom" or "Bacillariophyceae") or ("Mollusca" or "mollusk" or "mollusc" or "Gastropoda" or "gastropod" or "Bivalvia" or "bivalve") or ("mammal" or "mammalian" or "mammoth" or "reindeer" or "caribou" or "Rangifer tarandus" or "polar bear" or "Mammuthus primigenius" or "steppe bison" or "Bison priscus" or "muskox" or "Ovibos moschatus" or "moose" or "Alcos alces" or "horse" or "Equus caballus" or "wolf" or "wolves" or "Canis lupus" or "dog" or "squirrel" or "rodent" or "Rodentia" or "Urocitellus" or "arctic fox" or "vulpes lagopus" or "alopex lagopus" or "reptile" or "bird?" or "Aves") or ("ostracod" or "ostracode" or "ostracoda") or ("coleoptera" or "beetle") or ("chironomid" or "Chironomidae") or ("insect" or "macroinvertebrate" or "macro-invertebrate") or ("amphibian" or "Amphibia" or "aquatic animal" or "algae" or "algal") or ("animal" or "fauna" or "faunal" or "megafauna" or "megaherbivore" or "vertebrata" or "vertebrate") or ("pollen" or "palynology" or "macrofossil" or "pal$eobotanical" or "leaf wax" or "palynomorph") or "organic biomarker" or ("ring width" or "wood ring" or "tree ring" or "stem sample" or "driftwood") or ("terrestrial proxies" or "terrestrial proxy")).bo. or ("plant?" or "flora" or "vegetation" or "Plantae" or "vegetational" or "pal?eovegetation" or "floristic" or ("bryophyte" or "Bryophyta" or "liverwort?" or "moss" or "mosses") or ("shrub?" or "woody plant" or "vascular plant" or "fern?" or "pteridophyt?" or "spermatopht?" or "xerophyte" or "tree species" or "coniferous" or "conifer" or "deciduous" or "herb" or "forb?") or ("forest species" or "treeline species" or "tree-line species" or "larch" or "Larix" or "spruce" or "picea" or "western hemlock" or "Tsuga" or "Sitka" or "alnus" or "alder" or "cottonwood" or "Populus" or "poplar" or "Pinus" or "pine" or "Betula" or "birch" or "willow" or "salix") or ("lichen?" or "sporomiella" or "dung fungal spore" or "fungi" or "fungal") or ("diatom" or "Bacillariophyceae") or ("Mollusca" or "mollusk" or "mollusc" or "Gastropoda" or "gastropod" or "Bivalvia" or "bivalve") or ("mammal" or "mammalian" or "mammoth" or "reindeer" or "caribou" or "Rangifer tarandus" or "polar bear" or "Mammuthus primigenius" or "steppe bison" or "Bison priscus" or "muskox" or "Ovibos moschatus" or "moose" or "Alcos alces" or "horse" or "Equus caballus" or "wolf" or "wolves" or "Canis lupus" or "dog" or "squirrel" or "rodent" or "Rodentia" or "Urocitellus" or "arctic fox" or "vulpes lagopus" or "alopex lagopus" or "reptile" or "bird?" or "Aves") or ("ostracod" or "ostracode" or "ostracoda") or ("coleoptera" or "beetle") or ("chironomid" or "Chironomidae") or ("insect" or "macroinvertebrate" or "macro-invertebrate") or ("amphibian" or "Amphibia" or "aquatic animal" or "algae" or "algal") or ("animal" or "fauna" or "faunal" or "megafauna" or "megaherbivore" or "vertebrata" or "vertebrate") or ("pollen" or "palynology" or "macrofossil" or "pal$eobotanical" or "leaf wax" or "palynomorph") or "organic biomarker" or ("ring width" or "wood ring" or "tree ring" or "stem sample" or "driftwood") or ("terrestrial proxies" or "terrestrial proxy")).hw.
4. ("biodiversity" or ("diversity" or "evenness") or ("richness" or "number of species") or ("composition" or "assemblage" or "pal$eocommunit*" or "compositional shift" or "species dominance") or ("presence" or "present" or "absence" or "absent" or "occurrence" or "occurred") or ("stability" or "persistence" or "persisted" or "disappeared" or "extinction") or "abundance" or ("succession" or "vegetation development" or "plant cover" or "zonation pattern") or ("distribution" or "distributed" or "distributional") or ("coloni?ed" or "coloni?ation" or "established" or "spread to") or ("northern limit" or "northern treeline" or "ecotone" or "habitat diversity") or ("warming" or "cooling" or "temperature" or "temperatures" or "warm period" or "cold period" or "summer warmth") or ("hydroclimate" or "precipitation") or ("pal$eoclimat*" or "past climate" or "climate of the past") or "lake level" or ("vegetation productivity" or "plant productivity" or "radial growth" or "annual growth" or "growth rate" or "fuel load") or ("climate variable" or "climate interpretation" or "proxy climate record") or ("snow cover" or "snow extent") or ("organic carbon release" or "permafrost carbon" or "soil carbon release") or ("sea ice extent" or "relative sea level" or "sea level rise") or ("hunting" or "husbandry" or "domestication" or "construction activity" or "settlement history")).ti. or ("biodiversity" or ("diversity" or "evenness") or ("richness" or "number of species") or ("composition" or "assemblage" or "pal$eocommunit*" or "compositional shift" or "species dominance") or ("presence" or "present" or "absence" or "absent" or "occurrence" or "occurred") or ("stability" or "persistence" or "persisted" or "disappeared" or "extinction") or "abundance" or ("succession" or "vegetation development" or "plant cover" or "zonation pattern") or ("distribution" or "distributed" or "distributional") or ("coloni?ed" or "coloni?ation" or "established" or "spread to") or ("northern limit" or "northern treeline" or "ecotone" or "habitat diversity") or ("warming" or "cooling" or "temperature" or "temperatures" or "warm period" or "cold period" or "summer warmth") or ("hydroclimate" or "precipitation") or ("pal$eoclimat*" or "past climate" or "climate of the past") or "lake level" or ("vegetation productivity" or "plant productivity" or "radial growth" or "annual growth" or "growth rate" or "fuel load") or ("climate variable" or "climate interpretation" or "proxy climate record") or ("snow cover" or "snow extent") or ("organic carbon release" or "permafrost carbon" or "soil carbon release") or ("sea ice extent" or "relative sea level" or "sea level rise") or ("hunting" or "husbandry" or "domestication" or "construction activity" or "settlement history")).ab. or ("biodiversity" or ("diversity" or "evenness") or ("richness" or "number of species") or ("composition" or "assemblage" or "pal$eocommunit*" or "compositional shift" or "species dominance") or ("presence" or "present" or "absence" or "absent" or "occurrence" or "occurred") or ("stability" or "persistence" or "persisted" or "disappeared" or "extinction") or "abundance" or ("succession" or "vegetation development" or "plant cover" or "zonation pattern") or ("distribution" or "distributed" or "distributional") or ("coloni?ed" or "coloni?ation" or "established" or "spread to") or ("northern limit" or "northern treeline" or "ecotone" or "habitat diversity") or ("warming" or "cooling" or "temperature" or "temperatures" or "warm period" or "cold period" or "summer warmth") or ("hydroclimate" or "precipitation") or ("pal$eoclimat*" or "past climate" or "climate of the past") or "lake level" or ("vegetation productivity" or "plant productivity" or "radial growth" or "annual growth" or "growth rate" or "fuel load") or ("climate variable" or "climate interpretation" or "proxy climate record") or ("snow cover" or "snow extent") or ("organic carbon release" or "permafrost carbon" or "soil carbon release") or ("sea ice extent" or "relative sea level" or "sea level rise") or ("hunting" or "husbandry" or "domestication" or "construction activity" or "settlement history")).br.

## Web of Science Core

(TI=(

("biodiversity") OR ("diversity" OR "evenness") OR ("richness" OR "number of species") OR ("composition" OR "assemblage" OR "pal$eocommunit*" OR "compositional shift" OR "species dominance") OR ("presence" OR "present" OR "absence" OR "absent" OR "occurrence" OR "occurred") OR ("stability" OR "persistence" OR "persisted" OR "disappeared" OR "extinction") OR ("abundance") OR ("succession" OR "vegetation development" OR "plant cover" OR "zonation pattern") OR ("distribution" OR "distributed" OR "distributional") OR ("coloni?ed" OR "coloni?ation" OR "established" OR "spread to") OR ("northern limit" OR "northern treeline" OR "ecotone" OR "habitat diversity") OR ("warming" OR "cooling" OR "temperature" OR "temperatures" OR "warm period" OR "cold period" OR "summer warmth") OR ("hydroclimate" OR "precipitation") OR ("pal$eoclimat*" OR "past climate" OR "climate of the past") OR ("lake level") OR ("vegetation productivity" OR "plant productivity" OR "radial growth" OR "annual growth" OR "growth rate" OR "fuel load") OR ("climate variable" OR "climate interpretation" OR "proxy climate record") OR ("snow cover" OR "snow extent") OR ("organic carbon release" OR "permafrost carbon" OR "soil carbon release") OR ("sea ice extent" OR "relative sea level" OR "sea level rise") OR ("hunting" OR "husbandry" OR "domestication" OR "construction activity" OR "settlement history")

) OR TS=(

("biodiversity") OR ("diversity" OR "evenness") OR ("richness" OR "number of species") OR ("composition" OR "assemblage" OR "pal$eocommunit*" OR "compositional shift" OR "species dominance") OR ("presence" OR "present" OR "absence" OR "absent" OR "occurrence" OR "occurred") OR ("stability" OR "persistence" OR "persisted" OR "disappeared" OR "extinction") OR ("abundance") OR ("succession" OR "vegetation development" OR "plant cover" OR "zonation pattern") OR ("distribution" OR "distributed" OR "distributional") OR ("coloni?ed" OR "coloni?ation" OR "established" OR "spread to") OR ("northern limit" OR "northern treeline" OR "ecotone" OR "habitat diversity") OR ("warming" OR "cooling" OR "temperature" OR "temperatures" OR "warm period" OR "cold period" OR "summer warmth") OR ("hydroclimate" OR "precipitation") OR ("pal$eoclimat*" OR "past climate" OR "climate of the past") OR ("lake level") OR ("vegetation productivity" OR "plant productivity" OR "radial growth" OR "annual growth" OR "growth rate" OR "fuel load") OR ("climate variable" OR "climate interpretation" OR "proxy climate record") OR ("snow cover" OR "snow extent") OR ("organic carbon release" OR "permafrost carbon" OR "soil carbon release") OR ("sea ice extent" OR "relative sea level" OR "sea level rise") OR ("hunting" OR "husbandry" OR "domestication" OR "construction activity" OR "settlement history")

) OR AK=(

("biodiversity") OR ("diversity" OR "evenness") OR ("richness" OR "number of species") OR ("composition" OR "assemblage" OR "pal$eocommunit*" OR "compositional shift" OR "species dominance") OR ("presence" OR "present" OR "absence" OR "absent" OR "occurrence" OR "occurred") OR ("stability" OR "persistence" OR "persisted" OR "disappeared" OR "extinction") OR ("abundance") OR ("succession" OR "vegetation development" OR "plant cover" OR "zonation pattern") OR ("distribution" OR "distributed" OR "distributional") OR ("coloni?ed" OR "coloni?ation" OR "established" OR "spread to") OR ("northern limit" OR "northern treeline" OR "ecotone" OR "habitat diversity") OR ("warming" OR "cooling" OR "temperature" OR "temperatures" OR "warm period" OR "cold period" OR "summer warmth") OR ("hydroclimate" OR "precipitation") OR ("pal$eoclimat*" OR "past climate" OR "climate of the past") OR ("lake level") OR ("vegetation productivity" OR "plant productivity" OR "radial growth" OR "annual growth" OR "growth rate" OR "fuel load") OR ("climate variable" OR "climate interpretation" OR "proxy climate record") OR ("snow cover" OR "snow extent") OR ("organic carbon release" OR "permafrost carbon" OR "soil carbon release") OR ("sea ice extent" OR "relative sea level" OR "sea level rise") OR ("hunting" OR "husbandry" OR "domestication" OR "construction activity" OR "settlement history")

) OR AB=(

("biodiversity") OR ("diversity" OR "evenness") OR ("richness" OR "number of species") OR ("composition" OR "assemblage" OR "pal$eocommunit*" OR "compositional shift" OR "species dominance") OR ("presence" OR "present" OR "absence" OR "absent" OR "occurrence" OR "occurred") OR ("stability" OR "persistence" OR "persisted" OR "disappeared" OR "extinction") OR ("abundance") OR ("succession" OR "vegetation development" OR "plant cover" OR "zonation pattern") OR ("distribution" OR "distributed" OR "distributional") OR ("coloni?ed" OR "coloni?ation" OR "established" OR "spread to") OR ("northern limit" OR "northern treeline" OR "ecotone" OR "habitat diversity") OR ("warming" OR "cooling" OR "temperature" OR "temperatures" OR "warm period" OR "cold period" OR "summer warmth") OR ("hydroclimate" OR "precipitation") OR ("pal$eoclimat*" OR "past climate" OR "climate of the past") OR ("lake level") OR ("vegetation productivity" OR "plant productivity" OR "radial growth" OR "annual growth" OR "growth rate" OR "fuel load") OR ("climate variable" OR "climate interpretation" OR "proxy climate record") OR ("snow cover" OR "snow extent") OR ("organic carbon release" OR "permafrost carbon" OR "soil carbon release") OR ("sea ice extent" OR "relative sea level" OR "sea level rise") OR ("hunting" OR "husbandry" OR "domestication" OR "construction activity" OR "settlement history")

) OR KP=(

("biodiversity") OR ("diversity" OR "evenness") OR ("richness" OR "number of species") OR ("composition" OR "assemblage" OR "pal$eocommunit*" OR "compositional shift" OR "species dominance") OR ("presence" OR "present" OR "absence" OR "absent" OR "occurrence" OR "occurred") OR ("stability" OR "persistence" OR "persisted" OR "disappeared" OR "extinction") OR ("abundance") OR ("succession" OR "vegetation development" OR "plant cover" OR "zonation pattern") OR ("distribution" OR "distributed" OR "distributional") OR ("coloni?ed" OR "coloni?ation" OR "established" OR "spread to") OR ("northern limit" OR "northern treeline" OR "ecotone" OR "habitat diversity") OR ("warming" OR "cooling" OR "temperature" OR "temperatures" OR "warm period" OR "cold period" OR "summer warmth") OR ("hydroclimate" OR "precipitation") OR ("pal$eoclimat*" OR "past climate" OR "climate of the past") OR ("lake level") OR ("vegetation productivity" OR "plant productivity" OR "radial growth" OR "annual growth" OR "growth rate" OR "fuel load") OR ("climate variable" OR "climate interpretation" OR "proxy climate record") OR ("snow cover" OR "snow extent") OR ("organic carbon release" OR "permafrost carbon" OR "soil carbon release") OR ("sea ice extent" OR "relative sea level" OR "sea level rise") OR ("hunting" OR "husbandry" OR "domestication" OR "construction activity" OR "settlement history")

))

AND

(TI=(

("arctic" OR "high-latitude" OR "oro-arctic" OR "North Polar region" OR "subarctic" OR "northern environ*") OR ("Canada" OR "Canadian" OR "Russia" OR "Russian Federation" OR "USSR" OR "RSFSR" OR "Russian Federation" OR "Russian" OR "Norway" OR "Norwegian" OR "Finland" OR "Finnish" OR "Sweden" OR "Iceland" OR "Icelandic" OR "Greenland" OR "Greenlandic" OR "Faroe") OR ("tundra" OR "permafrost" OR "mammoth steppe") OR ("Yukon Territory" OR "Northwest Territories" OR "Nunavut" OR "Baffin Island" OR "Belcher Islands" OR "Ellesmere Island" OR "Southampton Island" OR "Mackenzie River" OR "Great Slave Lake" OR "Great Bear Lake" OR "Yukon River" OR "Alaska" OR "Teshekpuk Lake" OR "Queen Elizabeth Islands" OR "Victoria Island" OR "Banks Island" OR "Wrangel Island" OR "Seward peninsula" OR "Hudson Bay") OR ("Siberia" OR "Siberian" OR "Lake Baikal" OR "Lena River" OR "New Siberian Islands" OR "Severnaya Zemlya" OR "Franz Josef Land" OR "Novaya Zemlya" OR "Chukotka Autonomous Okrug" OR "Kamchatka Krai" OR "Magadan Oblast" OR "Murmansk Oblast" OR "Sakha" OR "Arkhangelsk Oblast" OR "Irkutsk Oblast" OR "Khabarovsk Krai" OR "Komi Republic" OR "Krasnoyarsk Krai" OR "Republic of Karelia" OR "Sakhalin Oblast" OR "Tuva" OR "Tyumen Oblast" OR "Polar Urals" OR "Yamalia") OR ("Svalbard" OR "Spitsbergen" OR "Bjørnøya" OR "Jan Mayen" OR "Finnmark" OR "Troms" OR "Lapland" or "Lappi" OR "Grímsey" OR "Lappland" OR "Norrbotten" OR "Västerbotten" OR "North West Europe" OR "Northwest Europe" OR "north* Europe" OR "Scandes" OR "Kola Peninsula") OR ("beringia" OR "beringian") OR ("global data*")

) OR TS=(

("arctic" OR "high-latitude" OR "oro-arctic" OR "North Polar region" OR "subarctic" OR "northern environ*") OR ("Canada" OR "Canadian" OR "Russia" OR "Russian Federation" OR "USSR" OR "RSFSR" OR "Russian Federation" OR "Russian" OR "Norway" OR "Norwegian" OR "Finland" OR "Finnish" OR "Sweden" OR "Iceland" OR "Icelandic" OR "Greenland" OR "Greenlandic" OR "Faroe") OR ("tundra" OR "permafrost" OR "mammoth steppe") OR ("Yukon Territory" OR "Northwest Territories" OR "Nunavut" OR "Baffin Island" OR "Belcher Islands" OR "Ellesmere Island" OR "Southampton Island" OR "Mackenzie River" OR "Great Slave Lake" OR "Great Bear Lake" OR "Yukon River" OR "Alaska" OR "Teshekpuk Lake" OR "Queen Elizabeth Islands" OR "Victoria Island" OR "Banks Island" OR "Wrangel Island" OR "Seward peninsula" OR "Hudson Bay") OR ("Siberia" OR "Siberian" OR "Lake Baikal" OR "Lena River" OR "New Siberian Islands" OR "Severnaya Zemlya" OR "Franz Josef Land" OR "Novaya Zemlya" OR "Chukotka Autonomous Okrug" OR "Kamchatka Krai" OR "Magadan Oblast" OR "Murmansk Oblast" OR "Sakha" OR "Arkhangelsk Oblast" OR "Irkutsk Oblast" OR "Khabarovsk Krai" OR "Komi Republic" OR "Krasnoyarsk Krai" OR "Republic of Karelia" OR "Sakhalin Oblast" OR "Tuva" OR "Tyumen Oblast" OR "Polar Urals" OR "Yamalia") OR ("Svalbard" OR "Spitsbergen" OR "Bjørnøya" OR "Jan Mayen" OR "Finnmark" OR "Troms" OR "Lapland" or "Lappi" OR "Grímsey" OR "Lappland" OR "Norrbotten" OR "Västerbotten" OR "North West Europe" OR "Northwest Europe" OR "north* Europe" OR "Scandes" OR "Kola Peninsula") OR ("beringia" OR "beringian") OR ("global data*")

) OR AK=(

("arctic" OR "high-latitude" OR "oro-arctic" OR "North Polar region" OR "subarctic" OR "northern environ*") OR ("Canada" OR "Canadian" OR "Russia" OR "Russian Federation" OR "USSR" OR "RSFSR" OR "Russian Federation" OR "Russian" OR "Norway" OR "Norwegian" OR "Finland" OR "Finnish" OR "Sweden" OR "Iceland" OR "Icelandic" OR "Greenland" OR "Greenlandic" OR "Faroe") OR ("tundra" OR "permafrost" OR "mammoth steppe") OR ("Yukon Territory" OR "Northwest Territories" OR "Nunavut" OR "Baffin Island" OR "Belcher Islands" OR "Ellesmere Island" OR "Southampton Island" OR "Mackenzie River" OR "Great Slave Lake" OR "Great Bear Lake" OR "Yukon River" OR "Alaska" OR "Teshekpuk Lake" OR "Queen Elizabeth Islands" OR "Victoria Island" OR "Banks Island" OR "Wrangel Island" OR "Seward peninsula" OR "Hudson Bay") OR ("Siberia" OR "Siberian" OR "Lake Baikal" OR "Lena River" OR "New Siberian Islands" OR "Severnaya Zemlya" OR "Franz Josef Land" OR "Novaya Zemlya" OR "Chukotka Autonomous Okrug" OR "Kamchatka Krai" OR "Magadan Oblast" OR "Murmansk Oblast" OR "Sakha" OR "Arkhangelsk Oblast" OR "Irkutsk Oblast" OR "Khabarovsk Krai" OR "Komi Republic" OR "Krasnoyarsk Krai" OR "Republic of Karelia" OR "Sakhalin Oblast" OR "Tuva" OR "Tyumen Oblast" OR "Polar Urals" OR "Yamalia") OR ("Svalbard" OR "Spitsbergen" OR "Bjørnøya" OR "Jan Mayen" OR "Finnmark" OR "Troms" OR "Lapland" or "Lappi" OR "Grímsey" OR "Lappland" OR "Norrbotten" OR "Västerbotten" OR "North West Europe" OR "Northwest Europe" OR "north* Europe" OR "Scandes" OR "Kola Peninsula") OR ("beringia" OR "beringian") OR ("global data*")

) OR AB=(

("arctic" OR "high-latitude" OR "oro-arctic" OR "North Polar region" OR "subarctic" OR "northern environ*") OR ("Canada" OR "Canadian" OR "Russia" OR "Russian Federation" OR "USSR" OR "RSFSR" OR "Russian Federation" OR "Russian" OR "Norway" OR "Norwegian" OR "Finland" OR "Finnish" OR "Sweden" OR "Iceland" OR "Icelandic" OR "Greenland" OR "Greenlandic" OR "Faroe") OR ("tundra" OR "permafrost" OR "mammoth steppe") OR ("Yukon Territory" OR "Northwest Territories" OR "Nunavut" OR "Baffin Island" OR "Belcher Islands" OR "Ellesmere Island" OR "Southampton Island" OR "Mackenzie River" OR "Great Slave Lake" OR "Great Bear Lake" OR "Yukon River" OR "Alaska" OR "Teshekpuk Lake" OR "Queen Elizabeth Islands" OR "Victoria Island" OR "Banks Island" OR "Wrangel Island" OR "Seward peninsula" OR "Hudson Bay") OR ("Siberia" OR "Siberian" OR "Lake Baikal" OR "Lena River" OR "New Siberian Islands" OR "Severnaya Zemlya" OR "Franz Josef Land" OR "Novaya Zemlya" OR "Chukotka Autonomous Okrug" OR "Kamchatka Krai" OR "Magadan Oblast" OR "Murmansk Oblast" OR "Sakha" OR "Arkhangelsk Oblast" OR "Irkutsk Oblast" OR "Khabarovsk Krai" OR "Komi Republic" OR "Krasnoyarsk Krai" OR "Republic of Karelia" OR "Sakhalin Oblast" OR "Tuva" OR "Tyumen Oblast" OR "Polar Urals" OR "Yamalia") OR ("Svalbard" OR "Spitsbergen" OR "Bjørnøya" OR "Jan Mayen" OR "Finnmark" OR "Troms" OR "Lapland" or "Lappi" OR "Grímsey" OR "Lappland" OR "Norrbotten" OR "Västerbotten" OR "North West Europe" OR "Northwest Europe" OR "north* Europe" OR "Scandes" OR "Kola Peninsula") OR ("beringia" OR "beringian") OR ("global data*")

) OR KP=(

("arctic" OR "high-latitude" OR "oro-arctic" OR "North Polar region" OR "subarctic" OR "northern environ*") OR ("Canada" OR "Canadian" OR "Russia" OR "Russian Federation" OR "USSR" OR "RSFSR" OR "Russian Federation" OR "Russian" OR "Norway" OR "Norwegian" OR "Finland" OR "Finnish" OR "Sweden" OR "Iceland" OR "Icelandic" OR "Greenland" OR "Greenlandic" OR "Faroe") OR ("tundra" OR "permafrost" OR "mammoth steppe") OR ("Yukon Territory" OR "Northwest Territories" OR "Nunavut" OR "Baffin Island" OR "Belcher Islands" OR "Ellesmere Island" OR "Southampton Island" OR "Mackenzie River" OR "Great Slave Lake" OR "Great Bear Lake" OR "Yukon River" OR "Alaska" OR "Teshekpuk Lake" OR "Queen Elizabeth Islands" OR "Victoria Island" OR "Banks Island" OR "Wrangel Island" OR "Seward peninsula" OR "Hudson Bay") OR ("Siberia" OR "Siberian" OR "Lake Baikal" OR "Lena River" OR "New Siberian Islands" OR "Severnaya Zemlya" OR "Franz Josef Land" OR "Novaya Zemlya" OR "Chukotka Autonomous Okrug" OR "Kamchatka Krai" OR "Magadan Oblast" OR "Murmansk Oblast" OR "Sakha" OR "Arkhangelsk Oblast" OR "Irkutsk Oblast" OR "Khabarovsk Krai" OR "Komi Republic" OR "Krasnoyarsk Krai" OR "Republic of Karelia" OR "Sakhalin Oblast" OR "Tuva" OR "Tyumen Oblast" OR "Polar Urals" OR "Yamalia") OR ("Svalbard" OR "Spitsbergen" OR "Bjørnøya" OR "Jan Mayen" OR "Finnmark" OR "Troms" OR "Lapland" or "Lappi" OR "Grímsey" OR "Lappland" OR "Norrbotten" OR "Västerbotten" OR "North West Europe" OR "Northwest Europe" OR "north* Europe" OR "Scandes" OR "Kola Peninsula") OR ("beringia" OR "beringian") OR ("global data*")

))

AND

(TI=(

("reconstruct" OR "reconstruction" OR "reconstructed") OR ("pal$eo*" OR "micropal$entol*") OR ("arch$eolog*" OR "artefact") OR ("history" OR "historic site" OR "historical record") OR ("dendrochron*" OR "dendroclim*" OR "dendroeco*" OR "growth ring" OR "tree ring" OR "wood ring") OR ("radiocarbon" OR "radio-carbon" OR "AMS" OR "accelerator mass spectrometry") OR ("age determination" OR "years before present" OR "yr BP" OR "calibration of age" OR "age model" OR "age-depth model") OR ("1?,??? 14C yr B$P" OR "1? ??? 14C Yr B$P" OR "1?,??? yr B$P" OR "1? ??? yr B$P" OR "1?,??? cal yr B$P" OR "1? ??? cal yr B$P" OR "pre-1???" OR "pre-20th century" OR "1?th century" OR "last ?,??? years" OR "last 1?,??? years" OR "14C dates" OR "1? Cal Ka" OR "one century" OR "pre-industrial" OR "preindustrial") OR ("fossil" OR "sub-fossil" OR "macrofossil" OR "megafossil" OR "microfossil" OR "subfossil") OR ("palynolog*") OR ("prehistoric" OR "prehistory" OR "Iron Age" OR "Bronze Age" OR "Neolithic" OR "Mesolithic" OR "Late Upper Pal$eolithic" OR "BC") OR ("Holocene" OR "MidHolocene" OR "Mid-Holocene" OR "late Pleistocene" OR "end of the Pleistocene" OR "Late Quaternary" OR "Little Ice Age" OR "Medieval Climate Anomoly" OR "Younger Dryas" OR "MIS 1" OR "marine isotope stage 1") OR ("centennial" OR "centuries" OR "millenial" OR "millenia" OR "submillenial" OR "chronolog*" OR "temporal trend") OR ("preboreal chronozone" OR "boreal chronozone" OR "atlantic chronozone" OR "subboreal chronozone") OR ("sediment core" OR "sedimentary sequence" OR "lake sediment" OR "transfer function" OR "peat core" OR "peat bog core" OR "peat monolith") OR ("ancient DNA" OR "aDNA" OR "sedDNA" OR "sedimentary DNA") OR ("postglacial" OR "post-glacial" OR "deglaciation" OR "deglacial") OR ("last appearance date" OR "refugia") OR ("multiproxy" OR "proxy record" OR "proxy archive" OR "proxy" OR "proxy study" OR "proxy studies") OR ("long-term ecolog*" OR "long-term record")

) OR TS=(

("reconstruct" OR "reconstruction" OR "reconstructed") OR ("pal$eo*" OR "micropal$entol*") OR ("arch$eolog*" OR "artefact") OR ("history" OR "historic site" OR "historical record") OR ("dendrochron*" OR "dendroclim*" OR "dendroeco*" OR "growth ring" OR "tree ring" OR "wood ring") OR ("radiocarbon" OR "radio-carbon" OR "AMS" OR "accelerator mass spectrometry") OR ("age determination" OR "years before present" OR "yr BP" OR "calibration of age" OR "age model" OR "age-depth model") OR ("1?,??? 14C yr B$P" OR "1? ??? 14C Yr B$P" OR "1?,??? yr B$P" OR "1? ??? yr B$P" OR "1?,??? cal yr B$P" OR "1? ??? cal yr B$P" OR "pre-1???" OR "pre-20th century" OR "1?th century" OR "last ?,??? years" OR "last 1?,??? years" OR "14C dates" OR "1? Cal Ka" OR "one century" OR "pre-industrial" OR "preindustrial") OR ("fossil" OR "sub-fossil" OR "macrofossil" OR "megafossil" OR "microfossil" OR "subfossil") OR ("palynolog*") OR ("prehistoric" OR "prehistory" OR "Iron Age" OR "Bronze Age" OR "Neolithic" OR "Mesolithic" OR "Late Upper Pal$eolithic" OR "BC") OR ("Holocene" OR "MidHolocene" OR "Mid-Holocene" OR "late Pleistocene" OR "end of the Pleistocene" OR "Late Quaternary" OR "Little Ice Age" OR "Medieval Climate Anomoly" OR "Younger Dryas" OR "MIS 1" OR "marine isotope stage 1") OR ("centennial" OR "centuries" OR "millenial" OR "millenia" OR "submillenial" OR "chronolog*" OR "temporal trend") OR ("preboreal chronozone" OR "boreal chronozone" OR "atlantic chronozone" OR "subboreal chronozone") OR ("sediment core" OR "sedimentary sequence" OR "lake sediment" OR "transfer function" OR "peat core" OR "peat bog core" OR "peat monolith") OR ("ancient DNA" OR "aDNA" OR "sedDNA" OR "sedimentary DNA") OR ("postglacial" OR "post-glacial" OR "deglaciation" OR "deglacial") OR ("last appearance date" OR "refugia") OR ("multiproxy" OR "proxy record" OR "proxy archive" OR "proxy" OR "proxy study" OR "proxy studies") OR ("long-term ecolog*" OR "long-term record")

) OR AK=(

("reconstruct" OR "reconstruction" OR "reconstructed") OR ("pal$eo*" OR "micropal$entol*") OR ("arch$eolog*" OR "artefact") OR ("history" OR "historic site" OR "historical record") OR ("dendrochron*" OR "dendroclim*" OR "dendroeco*" OR "growth ring" OR "tree ring" OR "wood ring") OR ("radiocarbon" OR "radio-carbon" OR "AMS" OR "accelerator mass spectrometry") OR ("age determination" OR "years before present" OR "yr BP" OR "calibration of age" OR "age model" OR "age-depth model") OR ("1?,??? 14C yr B$P" OR "1? ??? 14C Yr B$P" OR "1?,??? yr B$P" OR "1? ??? yr B$P" OR "1?,??? cal yr B$P" OR "1? ??? cal yr B$P" OR "pre-1???" OR "pre-20th century" OR "1?th century" OR "last ?,??? years" OR "last 1?,??? years" OR "14C dates" OR "1? Cal Ka" OR "one century" OR "pre-industrial" OR "preindustrial") OR ("fossil" OR "sub-fossil" OR "macrofossil" OR "megafossil" OR "microfossil" OR "subfossil") OR ("palynolog*") OR ("prehistoric" OR "prehistory" OR "Iron Age" OR "Bronze Age" OR "Neolithic" OR "Mesolithic" OR "Late Upper Pal$eolithic" OR "BC") OR ("Holocene" OR "MidHolocene" OR "Mid-Holocene" OR "late Pleistocene" OR "end of the Pleistocene" OR "Late Quaternary" OR "Little Ice Age" OR "Medieval Climate Anomoly" OR "Younger Dryas" OR "MIS 1" OR "marine isotope stage 1") OR ("centennial" OR "centuries" OR "millenial" OR "millenia" OR "submillenial" OR "chronolog*" OR "temporal trend") OR ("preboreal chronozone" OR "boreal chronozone" OR "atlantic chronozone" OR "subboreal chronozone") OR ("sediment core" OR "sedimentary sequence" OR "lake sediment" OR "transfer function" OR "peat core" OR "peat bog core" OR "peat monolith") OR ("ancient DNA" OR "aDNA" OR "sedDNA" OR "sedimentary DNA") OR ("postglacial" OR "post-glacial" OR "deglaciation" OR "deglacial") OR ("last appearance date" OR "refugia") OR ("multiproxy" OR "proxy record" OR "proxy archive" OR "proxy" OR "proxy study" OR "proxy studies") OR ("long-term ecolog*" OR "long-term record")

) OR AB=(

("reconstruct" OR "reconstruction" OR "reconstructed") OR ("pal$eo*" OR "micropal$entol*") OR ("arch$eolog*" OR "artefact") OR ("history" OR "historic site" OR "historical record") OR ("dendrochron*" OR "dendroclim*" OR "dendroeco*" OR "growth ring" OR "tree ring" OR "wood ring") OR ("radiocarbon" OR "radio-carbon" OR "AMS" OR "accelerator mass spectrometry") OR ("age determination" OR "years before present" OR "yr BP" OR "calibration of age" OR "age model" OR "age-depth model") OR ("1?,??? 14C yr B$P" OR "1? ??? 14C Yr B$P" OR "1?,??? yr B$P" OR "1? ??? yr B$P" OR "1?,??? cal yr B$P" OR "1? ??? cal yr B$P" OR "pre-1???" OR "pre-20th century" OR "1?th century" OR "last ?,??? years" OR "last 1?,??? years" OR "14C dates" OR "1? Cal Ka" OR "one century" OR "pre-industrial" OR "preindustrial") OR ("fossil" OR "sub-fossil" OR "macrofossil" OR "megafossil" OR "microfossil" OR "subfossil") OR ("palynolog*") OR ("prehistoric" OR "prehistory" OR "Iron Age" OR "Bronze Age" OR "Neolithic" OR "Mesolithic" OR "Late Upper Pal$eolithic" OR "BC") OR ("Holocene" OR "MidHolocene" OR "Mid-Holocene" OR "late Pleistocene" OR "end of the Pleistocene" OR "Late Quaternary" OR "Little Ice Age" OR "Medieval Climate Anomoly" OR "Younger Dryas" OR "MIS 1" OR "marine isotope stage 1") OR ("centennial" OR "centuries" OR "millenial" OR "millenia" OR "submillenial" OR "chronolog*" OR "temporal trend") OR ("preboreal chronozone" OR "boreal chronozone" OR "atlantic chronozone" OR "subboreal chronozone") OR ("sediment core" OR "sedimentary sequence" OR "lake sediment" OR "transfer function" OR "peat core" OR "peat bog core" OR "peat monolith") OR ("ancient DNA" OR "aDNA" OR "sedDNA" OR "sedimentary DNA") OR ("postglacial" OR "post-glacial" OR "deglaciation" OR "deglacial") OR ("last appearance date" OR "refugia") OR ("multiproxy" OR "proxy record" OR "proxy archive" OR "proxy" OR "proxy study" OR "proxy studies") OR ("long-term ecolog*" OR "long-term record")

) OR KP=(

("reconstruct" OR "reconstruction" OR "reconstructed") OR ("pal$eo*" OR "micropal$entol*") OR ("arch$eolog*" OR "artefact") OR ("history" OR "historic site" OR "historical record") OR ("dendrochron*" OR "dendroclim*" OR "dendroeco*" OR "growth ring" OR "tree ring" OR "wood ring") OR ("radiocarbon" OR "radio-carbon" OR "AMS" OR "accelerator mass spectrometry") OR ("age determination" OR "years before present" OR "yr BP" OR "calibration of age" OR "age model" OR "age-depth model") OR ("1?,??? 14C yr B$P" OR "1? ??? 14C Yr B$P" OR "1?,??? yr B$P" OR "1? ??? yr B$P" OR "1?,??? cal yr B$P" OR "1? ??? cal yr B$P" OR "pre-1???" OR "pre-20th century" OR "1?th century" OR "last ?,??? years" OR "last 1?,??? years" OR "14C dates" OR "1? Cal Ka" OR "one century" OR "pre-industrial" OR "preindustrial") OR ("fossil" OR "sub-fossil" OR "macrofossil" OR "megafossil" OR "microfossil" OR "subfossil") OR ("palynolog*") OR ("prehistoric" OR "prehistory" OR "Iron Age" OR "Bronze Age" OR "Neolithic" OR "Mesolithic" OR "Late Upper Pal$eolithic" OR "BC") OR ("Holocene" OR "MidHolocene" OR "Mid-Holocene" OR "late Pleistocene" OR "end of the Pleistocene" OR "Late Quaternary" OR "Little Ice Age" OR "Medieval Climate Anomoly" OR "Younger Dryas" OR "MIS 1" OR "marine isotope stage 1") OR ("centennial" OR "centuries" OR "millenial" OR "millenia" OR "submillenial" OR "chronolog*" OR "temporal trend") OR ("preboreal chronozone" OR "boreal chronozone" OR "atlantic chronozone" OR "subboreal chronozone") OR ("sediment core" OR "sedimentary sequence" OR "lake sediment" OR "transfer function" OR "peat core" OR "peat bog core" OR "peat monolith") OR ("ancient DNA" OR "aDNA" OR "sedDNA" OR "sedimentary DNA") OR ("postglacial" OR "post-glacial" OR "deglaciation" OR "deglacial") OR ("last appearance date" OR "refugia") OR ("multiproxy" OR "proxy record" OR "proxy archive" OR "proxy" OR "proxy study" OR "proxy studies") OR ("long-term ecolog*" OR "long-term record")

))

AND

(TI=(

("plant?" OR "flora" OR "vegetation" OR "Plantae" OR "vegetational" OR "pal?eovegetation" OR "floristic") OR ("bryophyte" OR "Bryophyta" OR "liverwort?" OR "moss" OR "mosses") OR ("shrub?" OR "woody plant" OR "vascular plant" OR "fern?" OR "pteridophyt?" OR "spermatopht?" OR "xerophyte" OR "tree species" OR "coniferous" OR "conifer" OR "deciduous" OR "herb" OR "forb?") OR ("forest species" OR "treeline species" OR "tree-line species" OR "larch" OR "Larix" OR "spruce" OR "picea" OR "western hemlock" OR "Tsuga" OR "Sitka" OR "alnus" OR "alder" OR "cottonwood" OR "Populus" OR "poplar" OR "Pinus" OR "pine" OR "Betula" OR "birch" OR "willow" OR "salix") OR ("lichen?" OR "sporomiella" OR "dung fungal spore" OR "fungi" OR "fungal") OR ("diatom" OR "Bacillariophyceae") OR ("Mollusca" OR "mollusk" OR "mollusc" OR "Gastropoda" OR "gastropod" OR "Bivalvia" OR "bivalve") OR ("mammal" OR "mammalian" OR "mammoth" OR "reindeer" OR "caribou" OR "Rangifer tarandus" OR "polar bear" OR "Mammuthus primigenius" OR "steppe bison" OR "Bison priscus" OR "muskox" OR "Ovibos moschatus" OR "moose" OR "Alcos alces" OR "horse" OR "Equus caballus" OR "wolf" OR "wolves" OR "Canis lupus" OR "dog" OR "squirrel" OR "rodent" OR "Rodentia" OR "Urocitellus" OR "arctic fox" OR "vulpes lagopus" OR "alopex lagopus" OR "reptile" OR "bird?" OR "Aves") OR ("ostracod" OR "ostracode" OR "ostracoda") OR ("coleoptera" OR "beetle") OR ("chironomid" OR "Chironomidae") OR ("insect" OR "macroinvertebrate" OR "macro-invertebrate") OR ("amphibian" OR "Amphibia" OR "aquatic animal" OR "algae" OR "algal") OR ("animal" OR "fauna" OR "faunal" OR "megafauna" OR "megaherbivore" OR "vertebrata" OR "vertebrate") OR ("pollen" OR "palynology" OR "macrofossil" OR "pal$eobotanical" OR "leaf wax" OR "palynomorph") OR ("organic biomarker") OR ("ring width" OR "wood ring" OR "tree ring" OR "stem sample" OR "driftwood") OR ("terrestrial proxies" OR "terrestrial proxy") ) OR TS=(

("plant?" OR "flora" OR "vegetation" OR "Plantae" OR "vegetational" OR "pal?eovegetation" OR "floristic") OR ("bryophyte" OR "Bryophyta" OR "liverwort?" OR "moss" OR "mosses") OR ("shrub?" OR "woody plant" OR "vascular plant" OR "fern?" OR "pteridophyt?" OR "spermatopht?" OR "xerophyte" OR "tree species" OR "coniferous" OR "conifer" OR "deciduous" OR "herb" OR "forb?") OR ("forest species" OR "treeline species" OR "tree-line species" OR "larch" OR "Larix" OR "spruce" OR "picea" OR "western hemlock" OR "Tsuga" OR "Sitka" OR "alnus" OR "alder" OR "cottonwood" OR "Populus" OR "poplar" OR "Pinus" OR "pine" OR "Betula" OR "birch" OR "willow" OR "salix") OR ("lichen?" OR "sporomiella" OR "dung fungal spore" OR "fungi" OR "fungal") OR ("diatom" OR "Bacillariophyceae") OR ("Mollusca" OR "mollusk" OR "mollusc" OR "Gastropoda" OR "gastropod" OR "Bivalvia" OR "bivalve") OR ("mammal" OR "mammalian" OR "mammoth" OR "reindeer" OR "caribou" OR "Rangifer tarandus" OR "polar bear" OR "Mammuthus primigenius" OR "steppe bison" OR "Bison priscus" OR "muskox" OR "Ovibos moschatus" OR "moose" OR "Alcos alces" OR "horse" OR "Equus caballus" OR "wolf" OR "wolves" OR "Canis lupus" OR "dog" OR "squirrel" OR "rodent" OR "Rodentia" OR "Urocitellus" OR "arctic fox" OR "vulpes lagopus" OR "alopex lagopus" OR "reptile" OR "bird?" OR "Aves") OR ("ostracod" OR "ostracode" OR "ostracoda") OR ("coleoptera" OR "beetle") OR ("chironomid" OR "Chironomidae") OR ("insect" OR "macroinvertebrate" OR "macro-invertebrate") OR ("amphibian" OR "Amphibia" OR "aquatic animal" OR "algae" OR "algal") OR ("animal" OR "fauna" OR "faunal" OR "megafauna" OR "megaherbivore" OR "vertebrata" OR "vertebrate") OR ("pollen" OR "palynology" OR "macrofossil" OR "pal$eobotanical" OR "leaf wax" OR "palynomorph") OR ("organic biomarker") OR ("ring width" OR "wood ring" OR "tree ring" OR "stem sample" OR "driftwood") OR ("terrestrial proxies" OR "terrestrial proxy")

) OR AK=(

("plant?" OR "flora" OR "vegetation" OR "Plantae" OR "vegetational" OR "pal?eovegetation" OR "floristic") OR ("bryophyte" OR "Bryophyta" OR "liverwort?" OR "moss" OR "mosses") OR ("shrub?" OR "woody plant" OR "vascular plant" OR "fern?" OR "pteridophyt?" OR "spermatopht?" OR "xerophyte" OR "tree species" OR "coniferous" OR "conifer" OR "deciduous" OR "herb" OR "forb?") OR ("forest species" OR "treeline species" OR "tree-line species" OR "larch" OR "Larix" OR "spruce" OR "picea" OR "western hemlock" OR "Tsuga" OR "Sitka" OR "alnus" OR "alder" OR "cottonwood" OR "Populus" OR "poplar" OR "Pinus" OR "pine" OR "Betula" OR "birch" OR "willow" OR "salix") OR ("lichen?" OR "sporomiella" OR "dung fungal spore" OR "fungi" OR "fungal") OR ("diatom" OR "Bacillariophyceae") OR ("Mollusca" OR "mollusk" OR "mollusc" OR "Gastropoda" OR "gastropod" OR "Bivalvia" OR "bivalve") OR ("mammal" OR "mammalian" OR "mammoth" OR "reindeer" OR "caribou" OR "Rangifer tarandus" OR "polar bear" OR "Mammuthus primigenius" OR "steppe bison" OR "Bison priscus" OR "muskox" OR "Ovibos moschatus" OR "moose" OR "Alcos alces" OR "horse" OR "Equus caballus" OR "wolf" OR "wolves" OR "Canis lupus" OR "dog" OR "squirrel" OR "rodent" OR "Rodentia" OR "Urocitellus" OR "arctic fox" OR "vulpes lagopus" OR "alopex lagopus" OR "reptile" OR "bird?" OR "Aves") OR ("ostracod" OR "ostracode" OR "ostracoda") OR ("coleoptera" OR "beetle") OR ("chironomid" OR "Chironomidae") OR ("insect" OR "macroinvertebrate" OR "macro-invertebrate") OR ("amphibian" OR "Amphibia" OR "aquatic animal" OR "algae" OR "algal") OR ("animal" OR "fauna" OR "faunal" OR "megafauna" OR "megaherbivore" OR "vertebrata" OR "vertebrate") OR ("pollen" OR "palynology" OR "macrofossil" OR "pal$eobotanical" OR "leaf wax" OR "palynomorph") OR ("organic biomarker") OR ("ring width" OR "wood ring" OR "tree ring" OR "stem sample" OR "driftwood") OR ("terrestrial proxies" OR "terrestrial proxy")

) OR AB=(

("plant?" OR "flora" OR "vegetation" OR "Plantae" OR "vegetational" OR "pal?eovegetation" OR "floristic") OR ("bryophyte" OR "Bryophyta" OR "liverwort?" OR "moss" OR "mosses") OR ("shrub?" OR "woody plant" OR "vascular plant" OR "fern?" OR "pteridophyt?" OR "spermatopht?" OR "xerophyte" OR "tree species" OR "coniferous" OR "conifer" OR "deciduous" OR "herb" OR "forb?") OR ("forest species" OR "treeline species" OR "tree-line species" OR "larch" OR "Larix" OR "spruce" OR "picea" OR "western hemlock" OR "Tsuga" OR "Sitka" OR "alnus" OR "alder" OR "cottonwood" OR "Populus" OR "poplar" OR "Pinus" OR "pine" OR "Betula" OR "birch" OR "willow" OR "salix") OR ("lichen?" OR "sporomiella" OR "dung fungal spore" OR "fungi" OR "fungal") OR ("diatom" OR "Bacillariophyceae") OR ("Mollusca" OR "mollusk" OR "mollusc" OR "Gastropoda" OR "gastropod" OR "Bivalvia" OR "bivalve") OR ("mammal" OR "mammalian" OR "mammoth" OR "reindeer" OR "caribou" OR "Rangifer tarandus" OR "polar bear" OR "Mammuthus primigenius" OR "steppe bison" OR "Bison priscus" OR "muskox" OR "Ovibos moschatus" OR "moose" OR "Alcos alces" OR "horse" OR "Equus caballus" OR "wolf" OR "wolves" OR "Canis lupus" OR "dog" OR "squirrel" OR "rodent" OR "Rodentia" OR "Urocitellus" OR "arctic fox" OR "vulpes lagopus" OR "alopex lagopus" OR "reptile" OR "bird?" OR "Aves") OR ("ostracod" OR "ostracode" OR "ostracoda") OR ("coleoptera" OR "beetle") OR ("chironomid" OR "Chironomidae") OR ("insect" OR "macroinvertebrate" OR "macro-invertebrate") OR ("amphibian" OR "Amphibia" OR "aquatic animal" OR "algae" OR "algal") OR ("animal" OR "fauna" OR "faunal" OR "megafauna" OR "megaherbivore" OR "vertebrata" OR "vertebrate") OR ("pollen" OR "palynology" OR "macrofossil" OR "pal$eobotanical" OR "leaf wax" OR "palynomorph") OR ("organic biomarker") OR ("ring width" OR "wood ring" OR "tree ring" OR "stem sample" OR "driftwood") OR ("terrestrial proxies" OR "terrestrial proxy")

) OR KP=(

("plant?" OR "flora" OR "vegetation" OR "Plantae" OR "vegetational" OR "pal?eovegetation" OR "floristic") OR ("bryophyte" OR "Bryophyta" OR "liverwort?" OR "moss" OR "mosses") OR ("shrub?" OR "woody plant" OR "vascular plant" OR "fern?" OR "pteridophyt?" OR "spermatopht?" OR "xerophyte" OR "tree species" OR "coniferous" OR "conifer" OR "deciduous" OR "herb" OR "forb?") OR ("forest species" OR "treeline species" OR "tree-line species" OR "larch" OR "Larix" OR "spruce" OR "picea" OR "western hemlock" OR "Tsuga" OR "Sitka" OR "alnus" OR "alder" OR "cottonwood" OR "Populus" OR "poplar" OR "Pinus" OR "pine" OR "Betula" OR "birch" OR "willow" OR "salix") OR ("lichen?" OR "sporomiella" OR "dung fungal spore" OR "fungi" OR "fungal") OR ("diatom" OR "Bacillariophyceae") OR ("Mollusca" OR "mollusk" OR "mollusc" OR "Gastropoda" OR "gastropod" OR "Bivalvia" OR "bivalve") OR ("mammal" OR "mammalian" OR "mammoth" OR "reindeer" OR "caribou" OR "Rangifer tarandus" OR "polar bear" OR "Mammuthus primigenius" OR "steppe bison" OR "Bison priscus" OR "muskox" OR "Ovibos moschatus" OR "moose" OR "Alcos alces" OR "horse" OR "Equus caballus" OR "wolf" OR "wolves" OR "Canis lupus" OR "dog" OR "squirrel" OR "rodent" OR "Rodentia" OR "Urocitellus" OR "arctic fox" OR "vulpes lagopus" OR "alopex lagopus" OR "reptile" OR "bird?" OR "Aves") OR ("ostracod" OR "ostracode" OR "ostracoda") OR ("coleoptera" OR "beetle") OR ("chironomid" OR "Chironomidae") OR ("insect" OR "macroinvertebrate" OR "macro-invertebrate") OR ("amphibian" OR "Amphibia" OR "aquatic animal" OR "algae" OR "algal") OR ("animal" OR "fauna" OR "faunal" OR "megafauna" OR "megaherbivore" OR "vertebrata" OR "vertebrate") OR ("pollen" OR "palynology" OR "macrofossil" OR "pal$eobotanical" OR "leaf wax" OR "palynomorph") OR ("organic biomarker") OR ("ring width" OR "wood ring" OR "tree ring" OR "stem sample" OR "driftwood") OR ("terrestrial proxies" OR "terrestrial proxy")

))

Zoological Record

(TI=(

("biodiversity") OR ("diversity" OR "evenness") OR ("richness" OR "number of species") OR ("composition" OR "assemblage" OR "pal$eocommunit*" OR "compositional shift" OR "species dominance") OR ("presence" OR "present" OR "absence" OR "absent" OR "occurrence" OR "occurred") OR ("stability" OR "persistence" OR "persisted" OR "disappeared" OR "extinction") OR ("abundance") OR ("succession" OR "vegetation development" OR "plant cover" OR "zonation pattern") OR ("distribution" OR "distributed" OR "distributional") OR ("coloni?ed" OR "coloni?ation" OR "established" OR "spread to") OR ("northern limit" OR "northern treeline" OR "ecotone" OR "habitat diversity") OR ("warming" OR "cooling" OR "temperature" OR "temperatures" OR "warm period" OR "cold period" OR "summer warmth") OR ("hydroclimate" OR "precipitation") OR ("pal$eoclimat*" OR "past climate" OR "climate of the past") OR ("lake level") OR ("vegetation productivity" OR "plant productivity" OR "radial growth" OR "annual growth" OR "growth rate" OR "fuel load") OR ("climate variable" OR "climate interpretation" OR "proxy climate record") OR ("snow cover" OR "snow extent") OR ("organic carbon release" OR "permafrost carbon" OR "soil carbon release") OR ("sea ice extent" OR "relative sea level" OR "sea level rise") OR ("hunting" OR "husbandry" OR "domestication" OR "construction activity" OR "settlement history")

) OR TS=(

("biodiversity") OR ("diversity" OR "evenness") OR ("richness" OR "number of species") OR ("composition" OR "assemblage" OR "pal$eocommunit*" OR "compositional shift" OR "species dominance") OR ("presence" OR "present" OR "absence" OR "absent" OR "occurrence" OR "occurred") OR ("stability" OR "persistence" OR "persisted" OR "disappeared" OR "extinction") OR ("abundance") OR ("succession" OR "vegetation development" OR "plant cover" OR "zonation pattern") OR ("distribution" OR "distributed" OR "distributional") OR ("coloni?ed" OR "coloni?ation" OR "established" OR "spread to") OR ("northern limit" OR "northern treeline" OR "ecotone" OR "habitat diversity") OR ("warming" OR "cooling" OR "temperature" OR "temperatures" OR "warm period" OR "cold period" OR "summer warmth") OR ("hydroclimate" OR "precipitation") OR ("pal$eoclimat*" OR "past climate" OR "climate of the past") OR ("lake level") OR ("vegetation productivity" OR "plant productivity" OR "radial growth" OR "annual growth" OR "growth rate" OR "fuel load") OR ("climate variable" OR "climate interpretation" OR "proxy climate record") OR ("snow cover" OR "snow extent") OR ("organic carbon release" OR "permafrost carbon" OR "soil carbon release") OR ("sea ice extent" OR "relative sea level" OR "sea level rise") OR ("hunting" OR "husbandry" OR "domestication" OR "construction activity" OR "settlement history")

) OR AB=(

("biodiversity") OR ("diversity" OR "evenness") OR ("richness" OR "number of species") OR ("composition" OR "assemblage" OR "pal$eocommunit*" OR "compositional shift" OR "species dominance") OR ("presence" OR "present" OR "absence" OR "absent" OR "occurrence" OR "occurred") OR ("stability" OR "persistence" OR "persisted" OR "disappeared" OR "extinction") OR ("abundance") OR ("succession" OR "vegetation development" OR "plant cover" OR "zonation pattern") OR ("distribution" OR "distributed" OR "distributional") OR ("coloni?ed" OR "coloni?ation" OR "established" OR "spread to") OR ("northern limit" OR "northern treeline" OR "ecotone" OR "habitat diversity") OR ("warming" OR "cooling" OR "temperature" OR "temperatures" OR "warm period" OR "cold period" OR "summer warmth") OR ("hydroclimate" OR "precipitation") OR ("pal$eoclimat*" OR "past climate" OR "climate of the past") OR ("lake level") OR ("vegetation productivity" OR "plant productivity" OR "radial growth" OR "annual growth" OR "growth rate" OR "fuel load") OR ("climate variable" OR "climate interpretation" OR "proxy climate record") OR ("snow cover" OR "snow extent") OR ("organic carbon release" OR "permafrost carbon" OR "soil carbon release") OR ("sea ice extent" OR "relative sea level" OR "sea level rise") OR ("hunting" OR "husbandry" OR "domestication" OR "construction activity" OR "settlement history")

) OR DE=(

("biodiversity") OR ("diversity" OR "evenness") OR ("richness" OR "number of species") OR ("composition" OR "assemblage" OR "pal$eocommunit*" OR "compositional shift" OR "species dominance") OR ("presence" OR "present" OR "absence" OR "absent" OR "occurrence" OR "occurred") OR ("stability" OR "persistence" OR "persisted" OR "disappeared" OR "extinction") OR ("abundance") OR ("succession" OR "vegetation development" OR "plant cover" OR "zonation pattern") OR ("distribution" OR "distributed" OR "distributional") OR ("coloni?ed" OR "coloni?ation" OR "established" OR "spread to") OR ("northern limit" OR "northern treeline" OR "ecotone" OR "habitat diversity") OR ("warming" OR "cooling" OR "temperature" OR "temperatures" OR "warm period" OR "cold period" OR "summer warmth") OR ("hydroclimate" OR "precipitation") OR ("pal$eoclimat*" OR "past climate" OR "climate of the past") OR ("lake level") OR ("vegetation productivity" OR "plant productivity" OR "radial growth" OR "annual growth" OR "growth rate" OR "fuel load") OR ("climate variable" OR "climate interpretation" OR "proxy climate record") OR ("snow cover" OR "snow extent") OR ("organic carbon release" OR "permafrost carbon" OR "soil carbon release") OR ("sea ice extent" OR "relative sea level" OR "sea level rise") OR ("hunting" OR "husbandry" OR "domestication" OR "construction activity" OR "settlement history")

))

AND

(TI=(

("arctic" OR "high-latitude" OR "oro-arctic" OR "North Polar region" OR "subarctic" OR "northern environ*") OR ("Canada" OR "Canadian" OR "Russia" OR "Russian Federation" OR "USSR" OR "RSFSR" OR "Russian Federation" OR "Russian" OR "Norway" OR "Norwegian" OR "Finland" OR "Finnish" OR "Sweden" OR "Iceland" OR "Icelandic" OR "Greenland" OR "Greenlandic" OR "Faroe") OR ("tundra" OR "permafrost" OR "mammoth steppe") OR ("Yukon Territory" OR "Northwest Territories" OR "Nunavut" OR "Baffin Island" OR "Belcher Islands" OR "Ellesmere Island" OR "Southampton Island" OR "Mackenzie River" OR "Great Slave Lake" OR "Great Bear Lake" OR "Yukon River" OR "Alaska" OR "Teshekpuk Lake" OR "Queen Elizabeth Islands" OR "Victoria Island" OR "Banks Island" OR "Wrangel Island" OR "Seward peninsula" OR "Hudson Bay") OR ("Siberia" OR "Siberian" OR "Lake Baikal" OR "Lena River" OR "New Siberian Islands" OR "Severnaya Zemlya" OR "Franz Josef Land" OR "Novaya Zemlya" OR "Chukotka Autonomous Okrug" OR "Kamchatka Krai" OR "Magadan Oblast" OR "Murmansk Oblast" OR "Sakha" OR "Arkhangelsk Oblast" OR "Irkutsk Oblast" OR "Khabarovsk Krai" OR "Komi Republic" OR "Krasnoyarsk Krai" OR "Republic of Karelia" OR "Sakhalin Oblast" OR "Tuva" OR "Tyumen Oblast" OR "Polar Urals" OR "Yamalia") OR ("Svalbard" OR "Spitsbergen" OR "Bjørnøya" OR "Jan Mayen" OR "Finnmark" OR "Troms" OR "Lapland" or "Lappi" OR "Grímsey" OR "Lappland" OR "Norrbotten" OR "Västerbotten" OR "North West Europe" OR "Northwest Europe" OR "north* Europe" OR "Scandes" OR "Kola Peninsula") OR ("beringia" OR "beringian") OR ("global data*")

) OR TS=(

("arctic" OR "high-latitude" OR "oro-arctic" OR "North Polar region" OR "subarctic" OR "northern environ*") OR ("Canada" OR "Canadian" OR "Russia" OR "Russian Federation" OR "USSR" OR "RSFSR" OR "Russian Federation" OR "Russian" OR "Norway" OR "Norwegian" OR "Finland" OR "Finnish" OR "Sweden" OR "Iceland" OR "Icelandic" OR "Greenland" OR "Greenlandic" OR "Faroe") OR ("tundra" OR "permafrost" OR "mammoth steppe") OR ("Yukon Territory" OR "Northwest Territories" OR "Nunavut" OR "Baffin Island" OR "Belcher Islands" OR "Ellesmere Island" OR "Southampton Island" OR "Mackenzie River" OR "Great Slave Lake" OR "Great Bear Lake" OR "Yukon River" OR "Alaska" OR "Teshekpuk Lake" OR "Queen Elizabeth Islands" OR "Victoria Island" OR "Banks Island" OR "Wrangel Island" OR "Seward peninsula" OR "Hudson Bay") OR ("Siberia" OR "Siberian" OR "Lake Baikal" OR "Lena River" OR "New Siberian Islands" OR "Severnaya Zemlya" OR "Franz Josef Land" OR "Novaya Zemlya" OR "Chukotka Autonomous Okrug" OR "Kamchatka Krai" OR "Magadan Oblast" OR "Murmansk Oblast" OR "Sakha" OR "Arkhangelsk Oblast" OR "Irkutsk Oblast" OR "Khabarovsk Krai" OR "Komi Republic" OR "Krasnoyarsk Krai" OR "Republic of Karelia" OR "Sakhalin Oblast" OR "Tuva" OR "Tyumen Oblast" OR "Polar Urals" OR "Yamalia") OR ("Svalbard" OR "Spitsbergen" OR "Bjørnøya" OR "Jan Mayen" OR "Finnmark" OR "Troms" OR "Lapland" or "Lappi" OR "Grímsey" OR "Lappland" OR "Norrbotten" OR "Västerbotten" OR "North West Europe" OR "Northwest Europe" OR "north* Europe" OR "Scandes" OR "Kola Peninsula") OR ("beringia" OR "beringian") OR ("global data*")

) OR AB=(

("arctic" OR "high-latitude" OR "oro-arctic" OR "North Polar region" OR "subarctic" OR "northern environ*") OR ("Canada" OR "Canadian" OR "Russia" OR "Russian Federation" OR "USSR" OR "RSFSR" OR "Russian Federation" OR "Russian" OR "Norway" OR "Norwegian" OR "Finland" OR "Finnish" OR "Sweden" OR "Iceland" OR "Icelandic" OR "Greenland" OR "Greenlandic" OR "Faroe") OR ("tundra" OR "permafrost" OR "mammoth steppe") OR ("Yukon Territory" OR "Northwest Territories" OR "Nunavut" OR "Baffin Island" OR "Belcher Islands" OR "Ellesmere Island" OR "Southampton Island" OR "Mackenzie River" OR "Great Slave Lake" OR "Great Bear Lake" OR "Yukon River" OR "Alaska" OR "Teshekpuk Lake" OR "Queen Elizabeth Islands" OR "Victoria Island" OR "Banks Island" OR "Wrangel Island" OR "Seward peninsula" OR "Hudson Bay") OR ("Siberia" OR "Siberian" OR "Lake Baikal" OR "Lena River" OR "New Siberian Islands" OR "Severnaya Zemlya" OR "Franz Josef Land" OR "Novaya Zemlya" OR "Chukotka Autonomous Okrug" OR "Kamchatka Krai" OR "Magadan Oblast" OR "Murmansk Oblast" OR "Sakha" OR "Arkhangelsk Oblast" OR "Irkutsk Oblast" OR "Khabarovsk Krai" OR "Komi Republic" OR "Krasnoyarsk Krai" OR "Republic of Karelia" OR "Sakhalin Oblast" OR "Tuva" OR "Tyumen Oblast" OR "Polar Urals" OR "Yamalia") OR ("Svalbard" OR "Spitsbergen" OR "Bjørnøya" OR "Jan Mayen" OR "Finnmark" OR "Troms" OR "Lapland" or "Lappi" OR "Grímsey" OR "Lappland" OR "Norrbotten" OR "Västerbotten" OR "North West Europe" OR "Northwest Europe" OR "north* Europe" OR "Scandes" OR "Kola Peninsula") OR ("beringia" OR "beringian") OR ("global data*")

) OR DE=(

("arctic" OR "high-latitude" OR "oro-arctic" OR "North Polar region" OR "subarctic" OR "northern environ*") OR ("Canada" OR "Canadian" OR "Russia" OR "Russian Federation" OR "USSR" OR "RSFSR" OR "Russian Federation" OR "Russian" OR "Norway" OR "Norwegian" OR "Finland" OR "Finnish" OR "Sweden" OR "Iceland" OR "Icelandic" OR "Greenland" OR "Greenlandic" OR "Faroe") OR ("tundra" OR "permafrost" OR "mammoth steppe") OR ("Yukon Territory" OR "Northwest Territories" OR "Nunavut" OR "Baffin Island" OR "Belcher Islands" OR "Ellesmere Island" OR "Southampton Island" OR "Mackenzie River" OR "Great Slave Lake" OR "Great Bear Lake" OR "Yukon River" OR "Alaska" OR "Teshekpuk Lake" OR "Queen Elizabeth Islands" OR "Victoria Island" OR "Banks Island" OR "Wrangel Island" OR "Seward peninsula" OR "Hudson Bay") OR ("Siberia" OR "Siberian" OR "Lake Baikal" OR "Lena River" OR "New Siberian Islands" OR "Severnaya Zemlya" OR "Franz Josef Land" OR "Novaya Zemlya" OR "Chukotka Autonomous Okrug" OR "Kamchatka Krai" OR "Magadan Oblast" OR "Murmansk Oblast" OR "Sakha" OR "Arkhangelsk Oblast" OR "Irkutsk Oblast" OR "Khabarovsk Krai" OR "Komi Republic" OR "Krasnoyarsk Krai" OR "Republic of Karelia" OR "Sakhalin Oblast" OR "Tuva" OR "Tyumen Oblast" OR "Polar Urals" OR "Yamalia") OR ("Svalbard" OR "Spitsbergen" OR "Bjørnøya" OR "Jan Mayen" OR "Finnmark" OR "Troms" OR "Lapland" or "Lappi" OR "Grímsey" OR "Lappland" OR "Norrbotten" OR "Västerbotten" OR "North West Europe" OR "Northwest Europe" OR "north* Europe" OR "Scandes" OR "Kola Peninsula") OR ("beringia" OR "beringian") OR ("global data*")

) OR SD=(

("arctic" OR "high-latitude" OR "oro-arctic" OR "North Polar region" OR "subarctic" OR "northern environ*") OR ("Canada" OR "Canadian" OR "Russia" OR "Russian Federation" OR "USSR" OR "RSFSR" OR "Russian Federation" OR "Russian" OR "Norway" OR "Norwegian" OR "Finland" OR "Finnish" OR "Sweden" OR "Iceland" OR "Icelandic" OR "Greenland" OR "Greenlandic" OR "Faroe") OR ("tundra" OR "permafrost" OR "mammoth steppe") OR ("Yukon Territory" OR "Northwest Territories" OR "Nunavut" OR "Baffin Island" OR "Belcher Islands" OR "Ellesmere Island" OR "Southampton Island" OR "Mackenzie River" OR "Great Slave Lake" OR "Great Bear Lake" OR "Yukon River" OR "Alaska" OR "Teshekpuk Lake" OR "Queen Elizabeth Islands" OR "Victoria Island" OR "Banks Island" OR "Wrangel Island" OR "Seward peninsula" OR "Hudson Bay") OR ("Siberia" OR "Siberian" OR "Lake Baikal" OR "Lena River" OR "New Siberian Islands" OR "Severnaya Zemlya" OR "Franz Josef Land" OR "Novaya Zemlya" OR "Chukotka Autonomous Okrug" OR "Kamchatka Krai" OR "Magadan Oblast" OR "Murmansk Oblast" OR "Sakha" OR "Arkhangelsk Oblast" OR "Irkutsk Oblast" OR "Khabarovsk Krai" OR "Komi Republic" OR "Krasnoyarsk Krai" OR "Republic of Karelia" OR "Sakhalin Oblast" OR "Tuva" OR "Tyumen Oblast" OR "Polar Urals" OR "Yamalia") OR ("Svalbard" OR "Spitsbergen" OR "Bjørnøya" OR "Jan Mayen" OR "Finnmark" OR "Troms" OR "Lapland" or "Lappi" OR "Grímsey" OR "Lappland" OR "Norrbotten" OR "Västerbotten" OR "North West Europe" OR "Northwest Europe" OR "north* Europe" OR "Scandes" OR "Kola Peninsula") OR ("beringia" OR "beringian") OR ("global data*")

))

AND

(TI=(

("reconstruct" OR "reconstruction" OR "reconstructed") OR ("pal$eo*" OR "micropal$entol*") OR ("arch$eolog*" OR "artefact") OR ("history" OR "historic site" OR "historical record") OR ("dendrochron*" OR "dendroclim*" OR "dendroeco*" OR "growth ring" OR "tree ring" OR "wood ring") OR ("radiocarbon" OR "radio-carbon" OR "AMS" OR "accelerator mass spectrometry") OR ("age determination" OR "years before present" OR "yr BP" OR "calibration of age" OR "age model" OR "age-depth model") OR ("1?,??? 14C yr B$P" OR "1? ??? 14C Yr B$P" OR "1?,??? yr B$P" OR "1? ??? yr B$P" OR "1?,??? cal yr B$P" OR "1? ??? cal yr B$P" OR "pre-1???" OR "pre-20th century" OR "1?th century" OR "last ?,??? years" OR "last 1?,??? years" OR "14C dates" OR "1? Cal Ka" OR "one century" OR "pre-industrial" OR "preindustrial") OR ("fossil" OR "sub-fossil" OR "macrofossil" OR "megafossil" OR "microfossil" OR "subfossil") OR ("palynolog*") OR ("prehistoric" OR "prehistory" OR "Iron Age" OR "Bronze Age" OR "Neolithic" OR "Mesolithic" OR "Late Upper Pal$eolithic" OR "BC") OR ("Holocene" OR "MidHolocene" OR "Mid-Holocene" OR "late Pleistocene" OR "end of the Pleistocene" OR "Late Quaternary" OR "Little Ice Age" OR "Medieval Climate Anomoly" OR "Younger Dryas" OR "MIS 1" OR "marine isotope stage 1") OR ("centennial" OR "centuries" OR "millenial" OR "millenia" OR "submillenial" OR "chronolog*" OR "temporal trend") OR ("preboreal chronozone" OR "boreal chronozone" OR "atlantic chronozone" OR "subboreal chronozone") OR ("sediment core" OR "sedimentary sequence" OR "lake sediment" OR "transfer function" OR "peat core" OR "peat bog core" OR "peat monolith") OR ("ancient DNA" OR "aDNA" OR "sedDNA" OR "sedimentary DNA") OR ("postglacial" OR "post-glacial" OR "deglaciation" OR "deglacial") OR ("last appearance date" OR "refugia") OR ("multiproxy" OR "proxy record" OR "proxy archive" OR "proxy" OR "proxy study" OR "proxy studies") OR ("long-term ecolog*" OR "long-term record")

) OR TS=(

("reconstruct" OR "reconstruction" OR "reconstructed") OR ("pal$eo*" OR "micropal$entol*") OR ("arch$eolog*" OR "artefact") OR ("history" OR "historic site" OR "historical record") OR ("dendrochron*" OR "dendroclim*" OR "dendroeco*" OR "growth ring" OR "tree ring" OR "wood ring") OR ("radiocarbon" OR "radio-carbon" OR "AMS" OR "accelerator mass spectrometry") OR ("age determination" OR "years before present" OR "yr BP" OR "calibration of age" OR "age model" OR "age-depth model") OR ("1?,??? 14C yr B$P" OR "1? ??? 14C Yr B$P" OR "1?,??? yr B$P" OR "1? ??? yr B$P" OR "1?,??? cal yr B$P" OR "1? ??? cal yr B$P" OR "pre-1???" OR "pre-20th century" OR "1?th century" OR "last ?,??? years" OR "last 1?,??? years" OR "14C dates" OR "1? Cal Ka" OR "one century" OR "pre-industrial" OR "preindustrial") OR ("fossil" OR "sub-fossil" OR "macrofossil" OR "megafossil" OR "microfossil" OR "subfossil") OR ("palynolog*") OR ("prehistoric" OR "prehistory" OR "Iron Age" OR "Bronze Age" OR "Neolithic" OR "Mesolithic" OR "Late Upper Pal$eolithic" OR "BC") OR ("Holocene" OR "MidHolocene" OR "Mid-Holocene" OR "late Pleistocene" OR "end of the Pleistocene" OR "Late Quaternary" OR "Little Ice Age" OR "Medieval Climate Anomoly" OR "Younger Dryas" OR "MIS 1" OR "marine isotope stage 1") OR ("centennial" OR "centuries" OR "millenial" OR "millenia" OR "submillenial" OR "chronolog*" OR "temporal trend") OR ("preboreal chronozone" OR "boreal chronozone" OR "atlantic chronozone" OR "subboreal chronozone") OR ("sediment core" OR "sedimentary sequence" OR "lake sediment" OR "transfer function" OR "peat core" OR "peat bog core" OR "peat monolith") OR ("ancient DNA" OR "aDNA" OR "sedDNA" OR "sedimentary DNA") OR ("postglacial" OR "post-glacial" OR "deglaciation" OR "deglacial") OR ("last appearance date" OR "refugia") OR ("multiproxy" OR "proxy record" OR "proxy archive" OR "proxy" OR "proxy study" OR "proxy studies") OR ("long-term ecolog*" OR "long-term record")

) OR AB=(

("reconstruct" OR "reconstruction" OR "reconstructed") OR ("pal$eo*" OR "micropal$entol*") OR ("arch$eolog*" OR "artefact") OR ("history" OR "historic site" OR "historical record") OR ("dendrochron*" OR "dendroclim*" OR "dendroeco*" OR "growth ring" OR "tree ring" OR "wood ring") OR ("radiocarbon" OR "radio-carbon" OR "AMS" OR "accelerator mass spectrometry") OR ("age determination" OR "years before present" OR "yr BP" OR "calibration of age" OR "age model" OR "age-depth model") OR ("1?,??? 14C yr B$P" OR "1? ??? 14C Yr B$P" OR "1?,??? yr B$P" OR "1? ??? yr B$P" OR "1?,??? cal yr B$P" OR "1? ??? cal yr B$P" OR "pre-1???" OR "pre-20th century" OR "1?th century" OR "last ?,??? years" OR "last 1?,??? years" OR "14C dates" OR "1? Cal Ka" OR "one century" OR "pre-industrial" OR "preindustrial") OR ("fossil" OR "sub-fossil" OR "macrofossil" OR "megafossil" OR "microfossil" OR "subfossil") OR ("palynolog*") OR ("prehistoric" OR "prehistory" OR "Iron Age" OR "Bronze Age" OR "Neolithic" OR "Mesolithic" OR "Late Upper Pal$eolithic" OR "BC") OR ("Holocene" OR "MidHolocene" OR "Mid-Holocene" OR "late Pleistocene" OR "end of the Pleistocene" OR "Late Quaternary" OR "Little Ice Age" OR "Medieval Climate Anomoly" OR "Younger Dryas" OR "MIS 1" OR "marine isotope stage 1") OR ("centennial" OR "centuries" OR "millenial" OR "millenia" OR "submillenial" OR "chronolog*" OR "temporal trend") OR ("preboreal chronozone" OR "boreal chronozone" OR "atlantic chronozone" OR "subboreal chronozone") OR ("sediment core" OR "sedimentary sequence" OR "lake sediment" OR "transfer function" OR "peat core" OR "peat bog core" OR "peat monolith") OR ("ancient DNA" OR "aDNA" OR "sedDNA" OR "sedimentary DNA") OR ("postglacial" OR "post-glacial" OR "deglaciation" OR "deglacial") OR ("last appearance date" OR "refugia") OR ("multiproxy" OR "proxy record" OR "proxy archive" OR "proxy" OR "proxy study" OR "proxy studies") OR ("long-term ecolog*" OR "long-term record")

) OR DE=(

("reconstruct" OR "reconstruction" OR "reconstructed") OR ("pal$eo*" OR "micropal$entol*") OR ("arch$eolog*" OR "artefact") OR ("history" OR "historic site" OR "historical record") OR ("dendrochron*" OR "dendroclim*" OR "dendroeco*" OR "growth ring" OR "tree ring" OR "wood ring") OR ("radiocarbon" OR "radio-carbon" OR "AMS" OR "accelerator mass spectrometry") OR ("age determination" OR "years before present" OR "yr BP" OR "calibration of age" OR "age model" OR "age-depth model") OR ("1?,??? 14C yr B$P" OR "1? ??? 14C Yr B$P" OR "1?,??? yr B$P" OR "1? ??? yr B$P" OR "1?,??? cal yr B$P" OR "1? ??? cal yr B$P" OR "pre-1???" OR "pre-20th century" OR "1?th century" OR "last ?,??? years" OR "last 1?,??? years" OR "14C dates" OR "1? Cal Ka" OR "one century" OR "pre-industrial" OR "preindustrial") OR ("fossil" OR "sub-fossil" OR "macrofossil" OR "megafossil" OR "microfossil" OR "subfossil") OR ("palynolog*") OR ("prehistoric" OR "prehistory" OR "Iron Age" OR "Bronze Age" OR "Neolithic" OR "Mesolithic" OR "Late Upper Pal$eolithic" OR "BC") OR ("Holocene" OR "MidHolocene" OR "Mid-Holocene" OR "late Pleistocene" OR "end of the Pleistocene" OR "Late Quaternary" OR "Little Ice Age" OR "Medieval Climate Anomoly" OR "Younger Dryas" OR "MIS 1" OR "marine isotope stage 1") OR ("centennial" OR "centuries" OR "millenial" OR "millenia" OR "submillenial" OR "chronolog*" OR "temporal trend") OR ("preboreal chronozone" OR "boreal chronozone" OR "atlantic chronozone" OR "subboreal chronozone") OR ("sediment core" OR "sedimentary sequence" OR "lake sediment" OR "transfer function" OR "peat core" OR "peat bog core" OR "peat monolith") OR ("ancient DNA" OR "aDNA" OR "sedDNA" OR "sedimentary DNA") OR ("postglacial" OR "post-glacial" OR "deglaciation" OR "deglacial") OR ("last appearance date" OR "refugia") OR ("multiproxy" OR "proxy record" OR "proxy archive" OR "proxy" OR "proxy study" OR "proxy studies") OR ("long-term ecolog*" OR "long-term record")

))

AND

(TI=(

("plant?" OR "flora" OR "vegetation" OR "Plantae" OR "vegetational" OR "pal?eovegetation" OR "floristic") OR ("bryophyte" OR "Bryophyta" OR "liverwort?" OR "moss" OR "mosses") OR ("shrub?" OR "woody plant" OR "vascular plant" OR "fern?" OR "pteridophyt?" OR "spermatopht?" OR "xerophyte" OR "tree species" OR "coniferous" OR "conifer" OR "deciduous" OR "herb" OR "forb?") OR ("forest species" OR "treeline species" OR "tree-line species" OR "larch" OR "Larix" OR "spruce" OR "picea" OR "western hemlock" OR "Tsuga" OR "Sitka" OR "alnus" OR "alder" OR "cottonwood" OR "Populus" OR "poplar" OR "Pinus" OR "pine" OR "Betula" OR "birch" OR "willow" OR "salix") OR ("lichen?" OR "sporomiella" OR "dung fungal spore" OR "fungi" OR "fungal") OR ("diatom" OR "Bacillariophyceae") OR ("Mollusca" OR "mollusk" OR "mollusc" OR "Gastropoda" OR "gastropod" OR "Bivalvia" OR "bivalve") OR ("mammal" OR "mammalian" OR "mammoth" OR "reindeer" OR "caribou" OR "Rangifer tarandus" OR "polar bear" OR "Mammuthus primigenius" OR "steppe bison" OR "Bison priscus" OR "muskox" OR "Ovibos moschatus" OR "moose" OR "Alcos alces" OR "horse" OR "Equus caballus" OR "wolf" OR "wolves" OR "Canis lupus" OR "dog" OR "squirrel" OR "rodent" OR "Rodentia" OR "Urocitellus" OR "arctic fox" OR "vulpes lagopus" OR "alopex lagopus" OR "reptile" OR "bird?" OR "Aves") OR ("ostracod" OR "ostracode" OR "ostracoda") OR ("coleoptera" OR "beetle") OR ("chironomid" OR "Chironomidae") OR ("insect" OR "macroinvertebrate" OR "macro-invertebrate") OR ("amphibian" OR "Amphibia" OR "aquatic animal" OR "algae" OR "algal") OR ("animal" OR "fauna" OR "faunal" OR "megafauna" OR "megaherbivore" OR "vertebrata" OR "vertebrate") OR ("pollen" OR "palynology" OR "macrofossil" OR "pal$eobotanical" OR "leaf wax" OR "palynomorph") OR ("organic biomarker") OR ("ring width" OR "wood ring" OR "tree ring" OR "stem sample" OR "driftwood") OR ("terrestrial proxies" OR "terrestrial proxy") ) OR TS=(

("plant?" OR "flora" OR "vegetation" OR "Plantae" OR "vegetational" OR "pal?eovegetation" OR "floristic") OR ("bryophyte" OR "Bryophyta" OR "liverwort?" OR "moss" OR "mosses") OR ("shrub?" OR "woody plant" OR "vascular plant" OR "fern?" OR "pteridophyt?" OR "spermatopht?" OR "xerophyte" OR "tree species" OR "coniferous" OR "conifer" OR "deciduous" OR "herb" OR "forb?") OR ("forest species" OR "treeline species" OR "tree-line species" OR "larch" OR "Larix" OR "spruce" OR "picea" OR "western hemlock" OR "Tsuga" OR "Sitka" OR "alnus" OR "alder" OR "cottonwood" OR "Populus" OR "poplar" OR "Pinus" OR "pine" OR "Betula" OR "birch" OR "willow" OR "salix") OR ("lichen?" OR "sporomiella" OR "dung fungal spore" OR "fungi" OR "fungal") OR ("diatom" OR "Bacillariophyceae") OR ("Mollusca" OR "mollusk" OR "mollusc" OR "Gastropoda" OR "gastropod" OR "Bivalvia" OR "bivalve") OR ("mammal" OR "mammalian" OR "mammoth" OR "reindeer" OR "caribou" OR "Rangifer tarandus" OR "polar bear" OR "Mammuthus primigenius" OR "steppe bison" OR "Bison priscus" OR "muskox" OR "Ovibos moschatus" OR "moose" OR "Alcos alces" OR "horse" OR "Equus caballus" OR "wolf" OR "wolves" OR "Canis lupus" OR "dog" OR "squirrel" OR "rodent" OR "Rodentia" OR "Urocitellus" OR "arctic fox" OR "vulpes lagopus" OR "alopex lagopus" OR "reptile" OR "bird?" OR "Aves") OR ("ostracod" OR "ostracode" OR "ostracoda") OR ("coleoptera" OR "beetle") OR ("chironomid" OR "Chironomidae") OR ("insect" OR "macroinvertebrate" OR "macro-invertebrate") OR ("amphibian" OR "Amphibia" OR "aquatic animal" OR "algae" OR "algal") OR ("animal" OR "fauna" OR "faunal" OR "megafauna" OR "megaherbivore" OR "vertebrata" OR "vertebrate") OR ("pollen" OR "palynology" OR "macrofossil" OR "pal$eobotanical" OR "leaf wax" OR "palynomorph") OR ("organic biomarker") OR ("ring width" OR "wood ring" OR "tree ring" OR "stem sample" OR "driftwood") OR ("terrestrial proxies" OR "terrestrial proxy")

) OR AB=(

("plant?" OR "flora" OR "vegetation" OR "Plantae" OR "vegetational" OR "pal?eovegetation" OR "floristic") OR ("bryophyte" OR "Bryophyta" OR "liverwort?" OR "moss" OR "mosses") OR ("shrub?" OR "woody plant" OR "vascular plant" OR "fern?" OR "pteridophyt?" OR "spermatopht?" OR "xerophyte" OR "tree species" OR "coniferous" OR "conifer" OR "deciduous" OR "herb" OR "forb?") OR ("forest species" OR "treeline species" OR "tree-line species" OR "larch" OR "Larix" OR "spruce" OR "picea" OR "western hemlock" OR "Tsuga" OR "Sitka" OR "alnus" OR "alder" OR "cottonwood" OR "Populus" OR "poplar" OR "Pinus" OR "pine" OR "Betula" OR "birch" OR "willow" OR "salix") OR ("lichen?" OR "sporomiella" OR "dung fungal spore" OR "fungi" OR "fungal") OR ("diatom" OR "Bacillariophyceae") OR ("Mollusca" OR "mollusk" OR "mollusc" OR "Gastropoda" OR "gastropod" OR "Bivalvia" OR "bivalve") OR ("mammal" OR "mammalian" OR "mammoth" OR "reindeer" OR "caribou" OR "Rangifer tarandus" OR "polar bear" OR "Mammuthus primigenius" OR "steppe bison" OR "Bison priscus" OR "muskox" OR "Ovibos moschatus" OR "moose" OR "Alcos alces" OR "horse" OR "Equus caballus" OR "wolf" OR "wolves" OR "Canis lupus" OR "dog" OR "squirrel" OR "rodent" OR "Rodentia" OR "Urocitellus" OR "arctic fox" OR "vulpes lagopus" OR "alopex lagopus" OR "reptile" OR "bird?" OR "Aves") OR ("ostracod" OR "ostracode" OR "ostracoda") OR ("coleoptera" OR "beetle") OR ("chironomid" OR "Chironomidae") OR ("insect" OR "macroinvertebrate" OR "macro-invertebrate") OR ("amphibian" OR "Amphibia" OR "aquatic animal" OR "algae" OR "algal") OR ("animal" OR "fauna" OR "faunal" OR "megafauna" OR "megaherbivore" OR "vertebrata" OR "vertebrate") OR ("pollen" OR "palynology" OR "macrofossil" OR "pal$eobotanical" OR "leaf wax" OR "palynomorph") OR ("organic biomarker") OR ("ring width" OR "wood ring" OR "tree ring" OR "stem sample" OR "driftwood") OR ("terrestrial proxies" OR "terrestrial proxy")

) OR SY=(

("plant?" OR "flora" OR "vegetation" OR "Plantae" OR "vegetational" OR "pal?eovegetation" OR "floristic") OR ("bryophyte" OR "Bryophyta" OR "liverwort?" OR "moss" OR "mosses") OR ("shrub?" OR "woody plant" OR "vascular plant" OR "fern?" OR "pteridophyt?" OR "spermatopht?" OR "xerophyte" OR "tree species" OR "coniferous" OR "conifer" OR "deciduous" OR "herb" OR "forb?") OR ("forest species" OR "treeline species" OR "tree-line species" OR "larch" OR "Larix" OR "spruce" OR "picea" OR "western hemlock" OR "Tsuga" OR "Sitka" OR "alnus" OR "alder" OR "cottonwood" OR "Populus" OR "poplar" OR "Pinus" OR "pine" OR "Betula" OR "birch" OR "willow" OR "salix") OR ("lichen?" OR "sporomiella" OR "dung fungal spore" OR "fungi" OR "fungal") OR ("diatom" OR "Bacillariophyceae") OR ("Mollusca" OR "mollusk" OR "mollusc" OR "Gastropoda" OR "gastropod" OR "Bivalvia" OR "bivalve") OR ("mammal" OR "mammalian" OR "mammoth" OR "reindeer" OR "caribou" OR "Rangifer tarandus" OR "polar bear" OR "Mammuthus primigenius" OR "steppe bison" OR "Bison priscus" OR "muskox" OR "Ovibos moschatus" OR "moose" OR "Alcos alces" OR "horse" OR "Equus caballus" OR "wolf" OR "wolves" OR "Canis lupus" OR "dog" OR "squirrel" OR "rodent" OR "Rodentia" OR "Urocitellus" OR "arctic fox" OR "vulpes lagopus" OR "alopex lagopus" OR "reptile" OR "bird?" OR "Aves") OR ("ostracod" OR "ostracode" OR "ostracoda") OR ("coleoptera" OR "beetle") OR ("chironomid" OR "Chironomidae") OR ("insect" OR "macroinvertebrate" OR "macro-invertebrate") OR ("amphibian" OR "Amphibia" OR "aquatic animal" OR "algae" OR "algal") OR ("animal" OR "fauna" OR "faunal" OR "megafauna" OR "megaherbivore" OR "vertebrata" OR "vertebrate") OR ("pollen" OR "palynology" OR "macrofossil" OR "pal$eobotanical" OR "leaf wax" OR "palynomorph") OR ("organic biomarker") OR ("ring width" OR "wood ring" OR "tree ring" OR "stem sample" OR "driftwood") OR ("terrestrial proxies" OR "terrestrial proxy")

))

Russian Citation Index

(TI=(

("biodiversity") OR ("diversity" OR "evenness") OR ("richness" OR "number of species") OR ("composition" OR "assemblage" OR "pal$eocommunit*" OR "compositional shift" OR "species dominance") OR ("presence" OR "present" OR "absence" OR "absent" OR "occurrence" OR "occurred") OR ("stability" OR "persistence" OR "persisted" OR "disappeared" OR "extinction") OR ("abundance") OR ("succession" OR "vegetation development" OR "plant cover" OR "zonation pattern") OR ("distribution" OR "distributed" OR "distributional") OR ("coloni?ed" OR "coloni?ation" OR "established" OR "spread to") OR ("northern limit" OR "northern treeline" OR "ecotone" OR "habitat diversity") OR ("warming" OR "cooling" OR "temperature" OR "temperatures" OR "warm period" OR "cold period" OR "summer warmth") OR ("hydroclimate" OR "precipitation") OR ("pal$eoclimat*" OR "past climate" OR "climate of the past") OR ("lake level") OR ("vegetation productivity" OR "plant productivity" OR "radial growth" OR "annual growth" OR "growth rate" OR "fuel load") OR ("climate variable" OR "climate interpretation" OR "proxy climate record") OR ("snow cover" OR "snow extent") OR ("organic carbon release" OR "permafrost carbon" OR "soil carbon release") OR ("sea ice extent" OR "relative sea level" OR "sea level rise") OR ("hunting" OR "husbandry" OR "domestication" OR "construction activity" OR "settlement history")

) OR TS=(

("biodiversity") OR ("diversity" OR "evenness") OR ("richness" OR "number of species") OR ("composition" OR "assemblage" OR "pal$eocommunit*" OR "compositional shift" OR "species dominance") OR ("presence" OR "present" OR "absence" OR "absent" OR "occurrence" OR "occurred") OR ("stability" OR "persistence" OR "persisted" OR "disappeared" OR "extinction") OR ("abundance") OR ("succession" OR "vegetation development" OR "plant cover" OR "zonation pattern") OR ("distribution" OR "distributed" OR "distributional") OR ("coloni?ed" OR "coloni?ation" OR "established" OR "spread to") OR ("northern limit" OR "northern treeline" OR "ecotone" OR "habitat diversity") OR ("warming" OR "cooling" OR "temperature" OR "temperatures" OR "warm period" OR "cold period" OR "summer warmth") OR ("hydroclimate" OR "precipitation") OR ("pal$eoclimat*" OR "past climate" OR "climate of the past") OR ("lake level") OR ("vegetation productivity" OR "plant productivity" OR "radial growth" OR "annual growth" OR "growth rate" OR "fuel load") OR ("climate variable" OR "climate interpretation" OR "proxy climate record") OR ("snow cover" OR "snow extent") OR ("organic carbon release" OR "permafrost carbon" OR "soil carbon release") OR ("sea ice extent" OR "relative sea level" OR "sea level rise") OR ("hunting" OR "husbandry" OR "domestication" OR "construction activity" OR "settlement history")

) OR AB=(

("biodiversity") OR ("diversity" OR "evenness") OR ("richness" OR "number of species") OR ("composition" OR "assemblage" OR "pal$eocommunit*" OR "compositional shift" OR "species dominance") OR ("presence" OR "present" OR "absence" OR "absent" OR "occurrence" OR "occurred") OR ("stability" OR "persistence" OR "persisted" OR "disappeared" OR "extinction") OR ("abundance") OR ("succession" OR "vegetation development" OR "plant cover" OR "zonation pattern") OR ("distribution" OR "distributed" OR "distributional") OR ("coloni?ed" OR "coloni?ation" OR "established" OR "spread to") OR ("northern limit" OR "northern treeline" OR "ecotone" OR "habitat diversity") OR ("warming" OR "cooling" OR "temperature" OR "temperatures" OR "warm period" OR "cold period" OR "summer warmth") OR ("hydroclimate" OR "precipitation") OR ("pal$eoclimat*" OR "past climate" OR "climate of the past") OR ("lake level") OR ("vegetation productivity" OR "plant productivity" OR "radial growth" OR "annual growth" OR "growth rate" OR "fuel load") OR ("climate variable" OR "climate interpretation" OR "proxy climate record") OR ("snow cover" OR "snow extent") OR ("organic carbon release" OR "permafrost carbon" OR "soil carbon release") OR ("sea ice extent" OR "relative sea level" OR "sea level rise") OR ("hunting" OR "husbandry" OR "domestication" OR "construction activity" OR "settlement history")

))

AND

(TI=(

("arctic" OR "high-latitude" OR "oro-arctic" OR "North Polar region" OR "subarctic" OR "northern environ*") OR ("Canada" OR "Canadian" OR "Russia" OR "Russian Federation" OR "USSR" OR "RSFSR" OR "Russian Federation" OR "Russian" OR "Norway" OR "Norwegian" OR "Finland" OR "Finnish" OR "Sweden" OR "Iceland" OR "Icelandic" OR "Greenland" OR "Greenlandic" OR "Faroe") OR ("tundra" OR "permafrost" OR "mammoth steppe") OR ("Yukon Territory" OR "Northwest Territories" OR "Nunavut" OR "Baffin Island" OR "Belcher Islands" OR "Ellesmere Island" OR "Southampton Island" OR "Mackenzie River" OR "Great Slave Lake" OR "Great Bear Lake" OR "Yukon River" OR "Alaska" OR "Teshekpuk Lake" OR "Queen Elizabeth Islands" OR "Victoria Island" OR "Banks Island" OR "Wrangel Island" OR "Seward peninsula" OR "Hudson Bay") OR ("Siberia" OR "Siberian" OR "Lake Baikal" OR "Lena River" OR "New Siberian Islands" OR "Severnaya Zemlya" OR "Franz Josef Land" OR "Novaya Zemlya" OR "Chukotka Autonomous Okrug" OR "Kamchatka Krai" OR "Magadan Oblast" OR "Murmansk Oblast" OR "Sakha" OR "Arkhangelsk Oblast" OR "Irkutsk Oblast" OR "Khabarovsk Krai" OR "Komi Republic" OR "Krasnoyarsk Krai" OR "Republic of Karelia" OR "Sakhalin Oblast" OR "Tuva" OR "Tyumen Oblast" OR "Polar Urals" OR "Yamalia") OR ("Svalbard" OR "Spitsbergen" OR "Bjørnøya" OR "Jan Mayen" OR "Finnmark" OR "Troms" OR "Lapland" or "Lappi" OR "Grímsey" OR "Lappland" OR "Norrbotten" OR "Västerbotten" OR "North West Europe" OR "Northwest Europe" OR "north* Europe" OR "Scandes" OR "Kola Peninsula") OR ("beringia" OR "beringian") OR ("global data*")

) OR TS=(

("arctic" OR "high-latitude" OR "oro-arctic" OR "North Polar region" OR "subarctic" OR "northern environ*") OR ("Canada" OR "Canadian" OR "Russia" OR "Russian Federation" OR "USSR" OR "RSFSR" OR "Russian Federation" OR "Russian" OR "Norway" OR "Norwegian" OR "Finland" OR "Finnish" OR "Sweden" OR "Iceland" OR "Icelandic" OR "Greenland" OR "Greenlandic" OR "Faroe") OR ("tundra" OR "permafrost" OR "mammoth steppe") OR ("Yukon Territory" OR "Northwest Territories" OR "Nunavut" OR "Baffin Island" OR "Belcher Islands" OR "Ellesmere Island" OR "Southampton Island" OR "Mackenzie River" OR "Great Slave Lake" OR "Great Bear Lake" OR "Yukon River" OR "Alaska" OR "Teshekpuk Lake" OR "Queen Elizabeth Islands" OR "Victoria Island" OR "Banks Island" OR "Wrangel Island" OR "Seward peninsula" OR "Hudson Bay") OR ("Siberia" OR "Siberian" OR "Lake Baikal" OR "Lena River" OR "New Siberian Islands" OR "Severnaya Zemlya" OR "Franz Josef Land" OR "Novaya Zemlya" OR "Chukotka Autonomous Okrug" OR "Kamchatka Krai" OR "Magadan Oblast" OR "Murmansk Oblast" OR "Sakha" OR "Arkhangelsk Oblast" OR "Irkutsk Oblast" OR "Khabarovsk Krai" OR "Komi Republic" OR "Krasnoyarsk Krai" OR "Republic of Karelia" OR "Sakhalin Oblast" OR "Tuva" OR "Tyumen Oblast" OR "Polar Urals" OR "Yamalia") OR ("Svalbard" OR "Spitsbergen" OR "Bjørnøya" OR "Jan Mayen" OR "Finnmark" OR "Troms" OR "Lapland" or "Lappi" OR "Grímsey" OR "Lappland" OR "Norrbotten" OR "Västerbotten" OR "North West Europe" OR "Northwest Europe" OR "north* Europe" OR "Scandes" OR "Kola Peninsula") OR ("beringia" OR "beringian") OR ("global data*")

) OR AB=(

("arctic" OR "high-latitude" OR "oro-arctic" OR "North Polar region" OR "subarctic" OR "northern environ*") OR ("Canada" OR "Canadian" OR "Russia" OR "Russian Federation" OR "USSR" OR "RSFSR" OR "Russian Federation" OR "Russian" OR "Norway" OR "Norwegian" OR "Finland" OR "Finnish" OR "Sweden" OR "Iceland" OR "Icelandic" OR "Greenland" OR "Greenlandic" OR "Faroe") OR ("tundra" OR "permafrost" OR "mammoth steppe") OR ("Yukon Territory" OR "Northwest Territories" OR "Nunavut" OR "Baffin Island" OR "Belcher Islands" OR "Ellesmere Island" OR "Southampton Island" OR "Mackenzie River" OR "Great Slave Lake" OR "Great Bear Lake" OR "Yukon River" OR "Alaska" OR "Teshekpuk Lake" OR "Queen Elizabeth Islands" OR "Victoria Island" OR "Banks Island" OR "Wrangel Island" OR "Seward peninsula" OR "Hudson Bay") OR ("Siberia" OR "Siberian" OR "Lake Baikal" OR "Lena River" OR "New Siberian Islands" OR "Severnaya Zemlya" OR "Franz Josef Land" OR "Novaya Zemlya" OR "Chukotka Autonomous Okrug" OR "Kamchatka Krai" OR "Magadan Oblast" OR "Murmansk Oblast" OR "Sakha" OR "Arkhangelsk Oblast" OR "Irkutsk Oblast" OR "Khabarovsk Krai" OR "Komi Republic" OR "Krasnoyarsk Krai" OR "Republic of Karelia" OR "Sakhalin Oblast" OR "Tuva" OR "Tyumen Oblast" OR "Polar Urals" OR "Yamalia") OR ("Svalbard" OR "Spitsbergen" OR "Bjørnøya" OR "Jan Mayen" OR "Finnmark" OR "Troms" OR "Lapland" or "Lappi" OR "Grímsey" OR "Lappland" OR "Norrbotten" OR "Västerbotten" OR "North West Europe" OR "Northwest Europe" OR "north* Europe" OR "Scandes" OR "Kola Peninsula") OR ("beringia" OR "beringian") OR ("global data*")

))

AND

(TI=(

("reconstruct" OR "reconstruction" OR "reconstructed") OR ("pal$eo*" OR "micropal$entol*") OR ("arch$eolog*" OR "artefact") OR ("history" OR "historic site" OR "historical record") OR ("dendrochron*" OR "dendroclim*" OR "dendroeco*" OR "growth ring" OR "tree ring" OR "wood ring") OR ("radiocarbon" OR "radio-carbon" OR "AMS" OR "accelerator mass spectrometry") OR ("age determination" OR "years before present" OR "yr BP" OR "calibration of age" OR "age model" OR "age-depth model") OR ("1?,??? 14C yr B$P" OR "1? ??? 14C Yr B$P" OR "1?,??? yr B$P" OR "1? ??? yr B$P" OR "1?,??? cal yr B$P" OR "1? ??? cal yr B$P" OR "pre-1???" OR "pre-20th century" OR "1?th century" OR "last ?,??? years" OR "last 1?,??? years" OR "14C dates" OR "1? Cal Ka" OR "one century" OR "pre-industrial" OR "preindustrial") OR ("fossil" OR "sub-fossil" OR "macrofossil" OR "megafossil" OR "microfossil" OR "subfossil") OR ("palynolog*") OR ("prehistoric" OR "prehistory" OR "Iron Age" OR "Bronze Age" OR "Neolithic" OR "Mesolithic" OR "Late Upper Pal$eolithic" OR "BC") OR ("Holocene" OR "MidHolocene" OR "Mid-Holocene" OR "late Pleistocene" OR "end of the Pleistocene" OR "Late Quaternary" OR "Little Ice Age" OR "Medieval Climate Anomoly" OR "Younger Dryas" OR "MIS 1" OR "marine isotope stage 1") OR ("centennial" OR "centuries" OR "millenial" OR "millenia" OR "submillenial" OR "chronolog*" OR "temporal trend") OR ("preboreal chronozone" OR "boreal chronozone" OR "atlantic chronozone" OR "subboreal chronozone") OR ("sediment core" OR "sedimentary sequence" OR "lake sediment" OR "transfer function" OR "peat core" OR "peat bog core" OR "peat monolith") OR ("ancient DNA" OR "aDNA" OR "sedDNA" OR "sedimentary DNA") OR ("postglacial" OR "post-glacial" OR "deglaciation" OR "deglacial") OR ("last appearance date" OR "refugia") OR ("multiproxy" OR "proxy record" OR "proxy archive" OR "proxy" OR "proxy study" OR "proxy studies") OR ("long-term ecolog*" OR "long-term record")

) OR TS=(

("reconstruct" OR "reconstruction" OR "reconstructed") OR ("pal$eo*" OR "micropal$entol*") OR ("arch$eolog*" OR "artefact") OR ("history" OR "historic site" OR "historical record") OR ("dendrochron*" OR "dendroclim*" OR "dendroeco*" OR "growth ring" OR "tree ring" OR "wood ring") OR ("radiocarbon" OR "radio-carbon" OR "AMS" OR "accelerator mass spectrometry") OR ("age determination" OR "years before present" OR "yr BP" OR "calibration of age" OR "age model" OR "age-depth model") OR ("1?,??? 14C yr B$P" OR "1? ??? 14C Yr B$P" OR "1?,??? yr B$P" OR "1? ??? yr B$P" OR "1?,??? cal yr B$P" OR "1? ??? cal yr B$P" OR "pre-1???" OR "pre-20th century" OR "1?th century" OR "last ?,??? years" OR "last 1?,??? years" OR "14C dates" OR "1? Cal Ka" OR "one century" OR "pre-industrial" OR "preindustrial") OR ("fossil" OR "sub-fossil" OR "macrofossil" OR "megafossil" OR "microfossil" OR "subfossil") OR ("palynolog*") OR ("prehistoric" OR "prehistory" OR "Iron Age" OR "Bronze Age" OR "Neolithic" OR "Mesolithic" OR "Late Upper Pal$eolithic" OR "BC") OR ("Holocene" OR "MidHolocene" OR "Mid-Holocene" OR "late Pleistocene" OR "end of the Pleistocene" OR "Late Quaternary" OR "Little Ice Age" OR "Medieval Climate Anomoly" OR "Younger Dryas" OR "MIS 1" OR "marine isotope stage 1") OR ("centennial" OR "centuries" OR "millenial" OR "millenia" OR "submillenial" OR "chronolog*" OR "temporal trend") OR ("preboreal chronozone" OR "boreal chronozone" OR "atlantic chronozone" OR "subboreal chronozone") OR ("sediment core" OR "sedimentary sequence" OR "lake sediment" OR "transfer function" OR "peat core" OR "peat bog core" OR "peat monolith") OR ("ancient DNA" OR "aDNA" OR "sedDNA" OR "sedimentary DNA") OR ("postglacial" OR "post-glacial" OR "deglaciation" OR "deglacial") OR ("last appearance date" OR "refugia") OR ("multiproxy" OR "proxy record" OR "proxy archive" OR "proxy" OR "proxy study" OR "proxy studies") OR ("long-term ecolog*" OR "long-term record")

) OR AB=(

("reconstruct" OR "reconstruction" OR "reconstructed") OR ("pal$eo*" OR "micropal$entol*") OR ("arch$eolog*" OR "artefact") OR ("history" OR "historic site" OR "historical record") OR ("dendrochron*" OR "dendroclim*" OR "dendroeco*" OR "growth ring" OR "tree ring" OR "wood ring") OR ("radiocarbon" OR "radio-carbon" OR "AMS" OR "accelerator mass spectrometry") OR ("age determination" OR "years before present" OR "yr BP" OR "calibration of age" OR "age model" OR "age-depth model") OR ("1?,??? 14C yr B$P" OR "1? ??? 14C Yr B$P" OR "1?,??? yr B$P" OR "1? ??? yr B$P" OR "1?,??? cal yr B$P" OR "1? ??? cal yr B$P" OR "pre-1???" OR "pre-20th century" OR "1?th century" OR "last ?,??? years" OR "last 1?,??? years" OR "14C dates" OR "1? Cal Ka" OR "one century" OR "pre-industrial" OR "preindustrial") OR ("fossil" OR "sub-fossil" OR "macrofossil" OR "megafossil" OR "microfossil" OR "subfossil") OR ("palynolog*") OR ("prehistoric" OR "prehistory" OR "Iron Age" OR "Bronze Age" OR "Neolithic" OR "Mesolithic" OR "Late Upper Pal$eolithic" OR "BC") OR ("Holocene" OR "MidHolocene" OR "Mid-Holocene" OR "late Pleistocene" OR "end of the Pleistocene" OR "Late Quaternary" OR "Little Ice Age" OR "Medieval Climate Anomoly" OR "Younger Dryas" OR "MIS 1" OR "marine isotope stage 1") OR ("centennial" OR "centuries" OR "millenial" OR "millenia" OR "submillenial" OR "chronolog*" OR "temporal trend") OR ("preboreal chronozone" OR "boreal chronozone" OR "atlantic chronozone" OR "subboreal chronozone") OR ("sediment core" OR "sedimentary sequence" OR "lake sediment" OR "transfer function" OR "peat core" OR "peat bog core" OR "peat monolith") OR ("ancient DNA" OR "aDNA" OR "sedDNA" OR "sedimentary DNA") OR ("postglacial" OR "post-glacial" OR "deglaciation" OR "deglacial") OR ("last appearance date" OR "refugia") OR ("multiproxy" OR "proxy record" OR "proxy archive" OR "proxy" OR "proxy study" OR "proxy studies") OR ("long-term ecolog*" OR "long-term record")

))

AND

(TI=(

("plant?" OR "flora" OR "vegetation" OR "Plantae" OR "vegetational" OR "pal?eovegetation" OR "floristic") OR ("bryophyte" OR "Bryophyta" OR "liverwort?" OR "moss" OR "mosses") OR ("shrub?" OR "woody plant" OR "vascular plant" OR "fern?" OR "pteridophyt?" OR "spermatopht?" OR "xerophyte" OR "tree species" OR "coniferous" OR "conifer" OR "deciduous" OR "herb" OR "forb?") OR ("forest species" OR "treeline species" OR "tree-line species" OR "larch" OR "Larix" OR "spruce" OR "picea" OR "western hemlock" OR "Tsuga" OR "Sitka" OR "alnus" OR "alder" OR "cottonwood" OR "Populus" OR "poplar" OR "Pinus" OR "pine" OR "Betula" OR "birch" OR "willow" OR "salix") OR ("lichen?" OR "sporomiella" OR "dung fungal spore" OR "fungi" OR "fungal") OR ("diatom" OR "Bacillariophyceae") OR ("Mollusca" OR "mollusk" OR "mollusc" OR "Gastropoda" OR "gastropod" OR "Bivalvia" OR "bivalve") OR ("mammal" OR "mammalian" OR "mammoth" OR "reindeer" OR "caribou" OR "Rangifer tarandus" OR "polar bear" OR "Mammuthus primigenius" OR "steppe bison" OR "Bison priscus" OR "muskox" OR "Ovibos moschatus" OR "moose" OR "Alcos alces" OR "horse" OR "Equus caballus" OR "wolf" OR "wolves" OR "Canis lupus" OR "dog" OR "squirrel" OR "rodent" OR "Rodentia" OR "Urocitellus" OR "arctic fox" OR "vulpes lagopus" OR "alopex lagopus" OR "reptile" OR "bird?" OR "Aves") OR ("ostracod" OR "ostracode" OR "ostracoda") OR ("coleoptera" OR "beetle") OR ("chironomid" OR "Chironomidae") OR ("insect" OR "macroinvertebrate" OR "macro-invertebrate") OR ("amphibian" OR "Amphibia" OR "aquatic animal" OR "algae" OR "algal") OR ("animal" OR "fauna" OR "faunal" OR "megafauna" OR "megaherbivore" OR "vertebrata" OR "vertebrate") OR ("pollen" OR "palynology" OR "macrofossil" OR "pal$eobotanical" OR "leaf wax" OR "palynomorph") OR ("organic biomarker") OR ("ring width" OR "wood ring" OR "tree ring" OR "stem sample" OR "driftwood") OR ("terrestrial proxies" OR "terrestrial proxy") ) OR TS=(

("plant?" OR "flora" OR "vegetation" OR "Plantae" OR "vegetational" OR "pal?eovegetation" OR "floristic") OR ("bryophyte" OR "Bryophyta" OR "liverwort?" OR "moss" OR "mosses") OR ("shrub?" OR "woody plant" OR "vascular plant" OR "fern?" OR "pteridophyt?" OR "spermatopht?" OR "xerophyte" OR "tree species" OR "coniferous" OR "conifer" OR "deciduous" OR "herb" OR "forb?") OR ("forest species" OR "treeline species" OR "tree-line species" OR "larch" OR "Larix" OR "spruce" OR "picea" OR "western hemlock" OR "Tsuga" OR "Sitka" OR "alnus" OR "alder" OR "cottonwood" OR "Populus" OR "poplar" OR "Pinus" OR "pine" OR "Betula" OR "birch" OR "willow" OR "salix") OR ("lichen?" OR "sporomiella" OR "dung fungal spore" OR "fungi" OR "fungal") OR ("diatom" OR "Bacillariophyceae") OR ("Mollusca" OR "mollusk" OR "mollusc" OR "Gastropoda" OR "gastropod" OR "Bivalvia" OR "bivalve") OR ("mammal" OR "mammalian" OR "mammoth" OR "reindeer" OR "caribou" OR "Rangifer tarandus" OR "polar bear" OR "Mammuthus primigenius" OR "steppe bison" OR "Bison priscus" OR "muskox" OR "Ovibos moschatus" OR "moose" OR "Alcos alces" OR "horse" OR "Equus caballus" OR "wolf" OR "wolves" OR "Canis lupus" OR "dog" OR "squirrel" OR "rodent" OR "Rodentia" OR "Urocitellus" OR "arctic fox" OR "vulpes lagopus" OR "alopex lagopus" OR "reptile" OR "bird?" OR "Aves") OR ("ostracod" OR "ostracode" OR "ostracoda") OR ("coleoptera" OR "beetle") OR ("chironomid" OR "Chironomidae") OR ("insect" OR "macroinvertebrate" OR "macro-invertebrate") OR ("amphibian" OR "Amphibia" OR "aquatic animal" OR "algae" OR "algal") OR ("animal" OR "fauna" OR "faunal" OR "megafauna" OR "megaherbivore" OR "vertebrata" OR "vertebrate") OR ("pollen" OR "palynology" OR "macrofossil" OR "pal$eobotanical" OR "leaf wax" OR "palynomorph") OR ("organic biomarker") OR ("ring width" OR "wood ring" OR "tree ring" OR "stem sample" OR "driftwood") OR ("terrestrial proxies" OR "terrestrial proxy")

) OR AB=(

("plant?" OR "flora" OR "vegetation" OR "Plantae" OR "vegetational" OR "pal?eovegetation" OR "floristic") OR ("bryophyte" OR "Bryophyta" OR "liverwort?" OR "moss" OR "mosses") OR ("shrub?" OR "woody plant" OR "vascular plant" OR "fern?" OR "pteridophyt?" OR "spermatopht?" OR "xerophyte" OR "tree species" OR "coniferous" OR "conifer" OR "deciduous" OR "herb" OR "forb?") OR ("forest species" OR "treeline species" OR "tree-line species" OR "larch" OR "Larix" OR "spruce" OR "picea" OR "western hemlock" OR "Tsuga" OR "Sitka" OR "alnus" OR "alder" OR "cottonwood" OR "Populus" OR "poplar" OR "Pinus" OR "pine" OR "Betula" OR "birch" OR "willow" OR "salix") OR ("lichen?" OR "sporomiella" OR "dung fungal spore" OR "fungi" OR "fungal") OR ("diatom" OR "Bacillariophyceae") OR ("Mollusca" OR "mollusk" OR "mollusc" OR "Gastropoda" OR "gastropod" OR "Bivalvia" OR "bivalve") OR ("mammal" OR "mammalian" OR "mammoth" OR "reindeer" OR "caribou" OR "Rangifer tarandus" OR "polar bear" OR "Mammuthus primigenius" OR "steppe bison" OR "Bison priscus" OR "muskox" OR "Ovibos moschatus" OR "moose" OR "Alcos alces" OR "horse" OR "Equus caballus" OR "wolf" OR "wolves" OR "Canis lupus" OR "dog" OR "squirrel" OR "rodent" OR "Rodentia" OR "Urocitellus" OR "arctic fox" OR "vulpes lagopus" OR "alopex lagopus" OR "reptile" OR "bird?" OR "Aves") OR ("ostracod" OR "ostracode" OR "ostracoda") OR ("coleoptera" OR "beetle") OR ("chironomid" OR "Chironomidae") OR ("insect" OR "macroinvertebrate" OR "macro-invertebrate") OR ("amphibian" OR "Amphibia" OR "aquatic animal" OR "algae" OR "algal") OR ("animal" OR "fauna" OR "faunal" OR "megafauna" OR "megaherbivore" OR "vertebrata" OR "vertebrate") OR ("pollen" OR "palynology" OR "macrofossil" OR "pal$eobotanical" OR "leaf wax" OR "palynomorph") OR ("organic biomarker") OR ("ring width" OR "wood ring" OR "tree ring" OR "stem sample" OR "driftwood") OR ("terrestrial proxies" OR "terrestrial proxy")

))

Zoological Record Archive via Ovid

Searches 1 to 4 combined using AND:

1. ("arctic" or "high-latitude" or "oro-arctic" or "North Polar region" or "subarctic" or "northern environ*" or ("Canada" or "Canadian" or "Russia" or "Russian Federation" or "USSR" or "RSFSR" or "Russian Federation" or "Russian" or "Norway" or "Norwegian" or "Finland" or "Finnish" or "Sweden" or "Iceland" or "Icelandic" or "Greenland" or "Greenlandic" or "Faroe") or ("tundra" or "permafrost" or "mammoth steppe") or ("Yukon Territory" or "Northwest Territories" or "Nunavut" or "Baffin Island" or "Belcher Islands" or "Ellesmere Island" or "Southampton Island" or "Mackenzie River" or "Great Slave Lake" or "Great Bear Lake" or "Yukon River" or "Alaska" or "Teshekpuk Lake" or "Queen Elizabeth Islands" or "Victoria Island" or "Banks Island" or "Wrangel Island" or "Seward peninsula" or "Hudson Bay") or ("Siberia" or "Siberian" or "Lake Baikal" or "Lena River" or "New Siberian Islands" or "Severnaya Zemlya" or "Franz Josef Land" or "Novaya Zemlya" or "Chukotka Autonomous Okrug" or "Kamchatka Krai" or "Magadan Oblast" or "Murmansk Oblast" or "Sakha" or "Arkhangelsk Oblast" or "Irkutsk Oblast" or "Khabarovsk Krai" or "Komi Republic" or "Krasnoyarsk Krai" or "Republic of Karelia" or "Sakhalin Oblast" or "Tuva" or "Tyumen Oblast" or "Polar Urals" or "Yamalia") or ("Svalbard" or "Spitsbergen" or "Bjørnøya" or "Jan Mayen" or "Finnmark" or "Troms" or "Lapland" or "Lappi" or "Grímsey" or "Lappland" or "Norrbotten" or "Västerbotten" or "North West Europe" or "Northwest Europe" or "north* Europe" or "Scandes" or "Kola Peninsula") or ("beringia" or "beringian") or "global data*").ti. or ("arctic" or "high-latitude" or "oro-arctic" or "North Polar region" or "subarctic" or "northern environ*" or ("Canada" or "Canadian" or "Russia" or "Russian Federation" or "USSR" or "RSFSR" or "Russian Federation" or "Russian" or "Norway" or "Norwegian" or "Finland" or "Finnish" or "Sweden" or "Iceland" or "Icelandic" or "Greenland" or "Greenlandic" or "Faroe") or ("tundra" or "permafrost" or "mammoth steppe") or ("Yukon Territory" or "Northwest Territories" or "Nunavut" or "Baffin Island" or "Belcher Islands" or "Ellesmere Island" or "Southampton Island" or "Mackenzie River" or "Great Slave Lake" or "Great Bear Lake" or "Yukon River" or "Alaska" or "Teshekpuk Lake" or "Queen Elizabeth Islands" or "Victoria Island" or "Banks Island" or "Wrangel Island" or "Seward peninsula" or "Hudson Bay") or ("Siberia" or "Siberian" or "Lake Baikal" or "Lena River" or "New Siberian Islands" or "Severnaya Zemlya" or "Franz Josef Land" or "Novaya Zemlya" or "Chukotka Autonomous Okrug" or "Kamchatka Krai" or "Magadan Oblast" or "Murmansk Oblast" or "Sakha" or "Arkhangelsk Oblast" or "Irkutsk Oblast" or "Khabarovsk Krai" or "Komi Republic" or "Krasnoyarsk Krai" or "Republic of Karelia" or "Sakhalin Oblast" or "Tuva" or "Tyumen Oblast" or "Polar Urals" or "Yamalia") or ("Svalbard" or "Spitsbergen" or "Bjørnøya" or "Jan Mayen" or "Finnmark" or "Troms" or "Lapland" or "Lappi" or "Grímsey" or "Lappland" or "Norrbotten" or "Västerbotten" or "North West Europe" or "Northwest Europe" or "north* Europe" or "Scandes" or "Kola Peninsula") or ("beringia" or "beringian") or "global data*").ab. or ("arctic" or "high-latitude" or "oro-arctic" or "North Polar region" or "subarctic" or "northern environ*" or ("Canada" or "Canadian" or "Russia" or "Russian Federation" or "USSR" or "RSFSR" or "Russian Federation" or "Russian" or "Norway" or "Norwegian" or "Finland" or "Finnish" or "Sweden" or "Iceland" or "Icelandic" or "Greenland" or "Greenlandic" or "Faroe") or ("tundra" or "permafrost" or "mammoth steppe") or ("Yukon Territory" or "Northwest Territories" or "Nunavut" or "Baffin Island" or "Belcher Islands" or "Ellesmere Island" or "Southampton Island" or "Mackenzie River" or "Great Slave Lake" or "Great Bear Lake" or "Yukon River" or "Alaska" or "Teshekpuk Lake" or "Queen Elizabeth Islands" or "Victoria Island" or "Banks Island" or "Wrangel Island" or "Seward peninsula" or "Hudson Bay") or ("Siberia" or "Siberian" or "Lake Baikal" or "Lena River" or "New Siberian Islands" or "Severnaya Zemlya" or "Franz Josef Land" or "Novaya Zemlya" or "Chukotka Autonomous Okrug" or "Kamchatka Krai" or "Magadan Oblast" or "Murmansk Oblast" or "Sakha" or "Arkhangelsk Oblast" or "Irkutsk Oblast" or "Khabarovsk Krai" or "Komi Republic" or "Krasnoyarsk Krai" or "Republic of Karelia" or "Sakhalin Oblast" or "Tuva" or "Tyumen Oblast" or "Polar Urals" or "Yamalia") or ("Svalbard" or "Spitsbergen" or "Bjørnøya" or "Jan Mayen" or "Finnmark" or "Troms" or "Lapland" or "Lappi" or "Grímsey" or "Lappland" or "Norrbotten" or "Västerbotten" or "North West Europe" or "Northwest Europe" or "north* Europe" or "Scandes" or "Kola Peninsula") or ("beringia" or "beringian") or "global data*").hw.
2. ("reconstruct" or "reconstruction" or "reconstructed" or ("pal$eo*" or "micropal$entol*") or ("arch$eolog*" or "artefact") or ("history" or "historic site" or "historical record") or ("dendrochron*" or "dendroclim*" or "dendroeco*" or "growth ring" or "tree ring" or "wood ring") or ("radiocarbon" or "radio-carbon" or "AMS" or "accelerator mass spectrometry") or ("age determination" or "years before present" or "yr BP" or "calibration of age" or "age model" or "age-depth model") or ("1?,??? 14C yr B$P" or "1? ??? 14C Yr B$P" or "1?,??? yr B$P" or "1? ??? yr B$P" or "1?,??? cal yr B$P" or "1? ??? cal yr B$P" or "pre-1???" or "pre-20th century" or "1?th century" or "last ?,??? years" or "last 1?,??? years" or "14C dates" or "1? Cal Ka" or "one century" or "pre-industrial" or "preindustrial") or ("fossil" or "sub-fossil" or "macrofossil" or "megafossil" or "microfossil" or "subfossil") or "palynolog*" or ("prehistoric" or "prehistory" or "Iron Age" or "Bronze Age" or "Neolithic" or "Mesolithic" or "Late Upper Pal$eolithic" or "BC") or ("Holocene" or "MidHolocene" or "Mid-Holocene" or "late Pleistocene" or "end of the Pleistocene" or "Late Quaternary" or "Little Ice Age" or "Medieval Climate Anomoly" or "Younger Dryas" or "MIS 1" or "marine isotope stage 1") or ("centennial" or "centuries" or "millenial" or "millenia" or "submillenial" or "chronolog*" or "temporal trend") or ("preboreal chronozone" or "boreal chronozone" or "atlantic chronozone" or "subboreal chronozone") or ("sediment core" or "sedimentary sequence" or "lake sediment" or "transfer function" or "peat core" or "peat bog core" or "peat monolith") or ("ancient DNA" or "aDNA" or "sedDNA" or "sedimentary DNA") or ("postglacial" or "post-glacial" or "deglaciation" or "deglacial") or ("last appearance date" or "refugia") or ("multiproxy" or "proxy record" or "proxy archive" or "proxy" or "proxy study" or "proxy studies") or ("long-term ecolog*" or "long-term record")).ti. or ("reconstruct" or "reconstruction" or "reconstructed" or ("pal$eo*" or "micropal$entol*") or ("arch$eolog*" or "artefact") or ("history" or "historic site" or "historical record") or ("dendrochron*" or "dendroclim*" or "dendroeco*" or "growth ring" or "tree ring" or "wood ring") or ("radiocarbon" or "radio-carbon" or "AMS" or "accelerator mass spectrometry") or ("age determination" or "years before present" or "yr BP" or "calibration of age" or "age model" or "age-depth model") or ("1?,??? 14C yr B$P" or "1? ??? 14C Yr B$P" or "1?,??? yr B$P" or "1? ??? yr B$P" or "1?,??? cal yr B$P" or "1? ??? cal yr B$P" or "pre-1???" or "pre-20th century" or "1?th century" or "last ?,??? years" or "last 1?,??? years" or "14C dates" or "1? Cal Ka" or "one century" or "pre- industrial" or "preindustrial") or ("fossil" or "sub-fossil" or "macrofossil" or "megafossil" or "microfossil" or "subfossil") or "palynolog*" or ("prehistoric" or "prehistory" or "Iron Age" or "Bronze Age" or "Neolithic" or "Mesolithic" or "Late Upper Pal$eolithic" or "BC") or ("Holocene" or "MidHolocene" or "Mid- Holocene" or "late Pleistocene" or "end of the Pleistocene" or "Late Quaternary" or "Little Ice Age" or "Medieval Climate Anomoly" or "Younger Dryas" or "MIS 1" or "marine isotope stage 1") or ("centennial" or "centuries" or "millenial" or "millenia" or "submillenial" or "chronolog*" or "temporal trend") or ("preboreal chronozone" or "boreal chronozone" or "atlantic chronozone" or "subboreal chronozone") or ("sediment core" or "sedimentary sequence" or "lake sediment" or "transfer function" or "peat core" or "peat bog core" or "peat monolith") or ("ancient DNA" or "aDNA" or "sedDNA" or "sedimentary DNA") or ("postglacial" or "post-glacial" or "deglaciation" or "deglacial") or ("last appearance date" or "refugia") or ("multiproxy" or "proxy record" or "proxy archive" or "proxy" or "proxy study" or "proxy studies") or ("long-term ecolog*" or "long-term record")).ab. or ("reconstruct" or "reconstruction" or "reconstructed" or ("pal$eo*" or "micropal$entol*") or ("arch$eolog*" or "artefact") or ("history" or "historic site" or "historical record") or ("dendrochron*" or "dendroclim*" or "dendroeco*" or "growth ring" or "tree ring" or "wood ring") or ("radiocarbon" or "radio-carbon" or "AMS" or "accelerator mass spectrometry") or ("age determination" or "years before present" or "yr BP" or "calibration of age" or "age model" or "age-depth model") or ("1?,??? 14C yr B$P" or "1? ??? 14C Yr B$P" or "1?,??? yr B$P" or "1? ??? yr B$P" or "1?,??? cal yr B$P" or "1? ??? cal yr B$P" or "pre-1???" or "pre-20th century" or "1?th century" or "last ?,??? years" or "last 1?,??? years" or "14C dates" or "1? Cal Ka" or "one century" or "pre-industrial" or "preindustrial") or ("fossil" or "sub-fossil" or "macrofossil" or "megafossil" or "microfossil" or "subfossil") or "palynolog*" or ("prehistoric" or "prehistory" or "Iron Age" or "Bronze Age" or "Neolithic" or "Mesolithic" or "Late Upper Pal$eolithic" or "BC") or ("Holocene" or "MidHolocene" or "Mid-Holocene" or "late Pleistocene" or "end of the Pleistocene" or "Late Quaternary" or "Little Ice Age" or "Medieval Climate Anomoly" or "Younger Dryas" or "MIS 1" or "marine isotope stage 1") or ("centennial" or "centuries" or "millenial" or "millenia" or "submillenial" or "chronolog*" or "temporal trend") or ("preboreal chronozone" or "boreal chronozone" or "atlantic chronozone" or "subboreal chronozone") or ("sediment core" or "sedimentary sequence" or "lake sediment" or "transfer function" or "peat core" or "peat bog core" or "peat monolith") or ("ancient DNA" or "aDNA" or "sedDNA" or "sedimentary DNA") or ("postglacial" or "post-glacial" or "deglaciation" or "deglacial") or ("last appearance date" or "refugia") or ("multiproxy" or "proxy record" or "proxy archive" or "proxy" or "proxy study" or "proxy studies") or ("long-term ecolog*" or "long-term record")).hw.
3. ("plant?" or "flora" or "vegetation" or "Plantae" or "vegetational" or "pal?eovegetation" or "floristic" or ("bryophyte" or "Bryophyta" or "liverwort?" or "moss" or "mosses") or ("shrub?" or "woody plant" or "vascular plant" or "fern?" or "pteridophyt?" or "spermatopht?" or "xerophyte" or "tree species" or "coniferous" or "conifer" or "deciduous" or "herb" or "forb?") or ("forest species" or "treeline species" or "tree-line species" or "larch" or "Larix" or "spruce" or "picea" or "western hemlock" or "Tsuga" or "Sitka" or "alnus" or "alder" or "cottonwood" or "Populus" or "poplar" or "Pinus" or "pine" or "Betula" or "birch" or "willow" or "salix") or ("lichen?" or "sporomiella" or "dung fungal spore" or "fungi" or "fungal") or ("diatom" or "Bacillariophyceae") or ("Mollusca" or "mollusk" or "mollusc" or "Gastropoda" or "gastropod" or "Bivalvia" or "bivalve") or ("mammal" or "mammalian" or "mammoth" or "reindeer" or "caribou" or "Rangifer tarandus" or "polar bear" or "Mammuthus primigenius" or "steppe bison" or "Bison priscus" or "muskox" or "Ovibos moschatus" or "moose" or "Alcos alces" or "horse" or "Equus caballus" or "wolf" or "wolves" or "Canis lupus" or "dog" or "squirrel" or "rodent" or "Rodentia" or "Urocitellus" or "arctic fox" or "vulpes lagopus" or "alopex lagopus" or "reptile" or "bird?" or "Aves") or ("ostracod" or "ostracode" or "ostracoda") or ("coleoptera" or "beetle") or ("chironomid" or "Chironomidae") or ("insect" or "macroinvertebrate" or "macro-invertebrate") or ("amphibian" or "Amphibia" or "aquatic animal" or "algae" or "algal") or ("animal" or "fauna" or "faunal" or "megafauna" or "megaherbivore" or "vertebrata" or "vertebrate") or ("pollen" or "palynology" or "macrofossil" or "pal$eobotanical" or "leaf wax" or "palynomorph") or "organic biomarker" or ("ring width" or "wood ring" or "tree ring" or "stem sample" or "driftwood") or ("terrestrial proxies" or "terrestrial proxy")).ti. or ("plant?" or "flora" or "vegetation" or "Plantae" or "vegetational" or "pal?eovegetation" or "floristic" or ("bryophyte" or "Bryophyta" or "liverwort?" or "moss" or "mosses") or ("shrub?" or "woody plant" or "vascular plant" or "fern?" or "pteridophyt?" or "spermatopht?" or "xerophyte" or "tree species" or "coniferous" or "conifer" or "deciduous" or "herb" or "forb?") or ("forest species" or "treeline species" or "tree-line species" or "larch" or "Larix" or "spruce" or "picea" or "western hemlock" or "Tsuga" or "Sitka" or "alnus" or "alder" or "cottonwood" or "Populus" or "poplar" or "Pinus" or "pine" or "Betula" or "birch" or "willow" or "salix") or ("lichen?" or "sporomiella" or "dung fungal spore" or "fungi" or "fungal") or ("diatom" or "Bacillariophyceae") or ("Mollusca" or "mollusk" or "mollusc" or "Gastropoda" or "gastropod" or "Bivalvia" or "bivalve") or ("mammal" or "mammalian" or "mammoth" or "reindeer" or "caribou" or "Rangifer tarandus" or "polar bear" or "Mammuthus primigenius" or "steppe bison" or "Bison priscus" or "muskox" or "Ovibos moschatus" or "moose" or "Alcos alces" or "horse" or "Equus caballus" or "wolf" or "wolves" or "Canis lupus" or "dog" or "squirrel" or "rodent" or "Rodentia" or "Urocitellus" or "arctic fox" or "vulpes lagopus" or "alopex lagopus" or "reptile" or "bird?" or "Aves") or ("ostracod" or "ostracode" or "ostracoda") or ("coleoptera" or "beetle") or ("chironomid" or "Chironomidae") or ("insect" or "macroinvertebrate" or "macro-invertebrate") or ("amphibian" or "Amphibia" or "aquatic animal" or "algae" or "algal") or ("animal" or "fauna" or "faunal" or "megafauna" or "megaherbivore" or "vertebrata" or "vertebrate") or ("pollen" or "palynology" or "macrofossil" or "pal$eobotanical" or "leaf wax" or "palynomorph") or "organic biomarker" or ("ring width" or "wood ring" or "tree ring" or "stem sample" or "driftwood") or ("terrestrial proxies" or "terrestrial proxy")).ab. or ("plant?" or "flora" or "vegetation" or "Plantae" or "vegetational" or "pal?eovegetation" or "floristic" or ("bryophyte" or "Bryophyta" or "liverwort?" or "moss" or "mosses") or ("shrub?" or "woody plant" or "vascular plant" or "fern?" or "pteridophyt?" or "spermatopht?" or "xerophyte" or "tree species" or "coniferous" or "conifer" or "deciduous" or "herb" or "forb?") or ("forest species" or "treeline species" or "tree-line species" or "larch" or "Larix" or "spruce" or "picea" or "western hemlock" or "Tsuga" or "Sitka" or "alnus" or "alder" or "cottonwood" or "Populus" or "poplar" or "Pinus" or "pine" or "Betula" or "birch" or "willow" or "salix") or ("lichen?" or "sporomiella" or "dung fungal spore" or "fungi" or "fungal") or ("diatom" or "Bacillariophyceae") or ("Mollusca" or "mollusk" or "mollusc" or "Gastropoda" or "gastropod" or "Bivalvia" or "bivalve") or ("mammal" or "mammalian" or "mammoth" or "reindeer" or "caribou" or "Rangifer tarandus" or "polar bear" or "Mammuthus primigenius" or "steppe bison" or "Bison priscus" or "muskox" or "Ovibos moschatus" or "moose" or "Alcos alces" or "horse" or "Equus caballus" or "wolf" or "wolves" or "Canis lupus" or "dog" or "squirrel" or "rodent" or "Rodentia" or "Urocitellus" or "arctic fox" or "vulpes lagopus" or "alopex lagopus" or "reptile" or "bird?" or "Aves") or ("ostracod" or "ostracode" or "ostracoda") or ("coleoptera" or "beetle") or ("chironomid" or "Chironomidae") or ("insect" or "macroinvertebrate" or "macro-invertebrate") or ("amphibian" or "Amphibia" or "aquatic animal" or "algae" or "algal") or ("animal" or "fauna" or "faunal" or "megafauna" or "megaherbivore" or "vertebrata" or "vertebrate") or ("pollen" or "palynology" or "macrofossil" or "pal$eobotanical" or "leaf wax" or "palynomorph") or "organic biomarker" or ("ring width" or "wood ring" or "tree ring" or "stem sample" or "driftwood") or ("terrestrial proxies" or "terrestrial proxy")).hw.
4. ("biodiversity" or ("diversity" or "evenness") or ("richness" or "number of species") or ("composition" or "assemblage" or "pal$eocommunit*" or "compositional shift" or "species dominance") or ("presence" or "present" or "absence" or "absent" or "occurrence" or "occurred") or ("stability" or "persistence" or "persisted" or "disappeared" or "extinction") or "abundance" or ("succession" or "vegetation development" or "plant cover" or "zonation pattern") or ("distribution" or "distributed" or "distributional") or ("coloni?ed" or "coloni?ation" or "established" or "spread to") or ("northern limit" or "northern treeline" or "ecotone" or "habitat diversity") or ("warming" or "cooling" or "temperature" or "temperatures" or "warm period" or "cold period" or "summer warmth") or ("hydroclimate" or "precipitation") or ("pal$eoclimat*" or "past climate" or "climate of the past") or "lake level" or ("vegetation productivity" or "plant productivity" or "radial growth" or "annual growth" or "growth rate" or "fuel load") or ("climate variable" or "climate interpretation" or "proxy climate record") or ("snow cover" or "snow extent") or ("organic carbon release" or "permafrost carbon" or "soil carbon release") or ("sea ice extent" or "relative sea level" or "sea level rise") or ("hunting" or "husbandry" or "domestication" or "construction activity" or "settlement history")).ti. or ("biodiversity" or ("diversity" or "evenness") or ("richness" or "number of species") or ("composition" or "assemblage" or "pal$eocommunit*" or "compositional shift" or "species dominance") or ("presence" or "present" or "absence" or "absent" or "occurrence" or "occurred") or ("stability" or "persistence" or "persisted" or "disappeared" or "extinction") or "abundance" or ("succession" or "vegetation development" or "plant cover" or "zonation pattern") or ("distribution" or "distributed" or "distributional") or ("coloni?ed" or "coloni?ation" or "established" or "spread to") or ("northern limit" or "northern treeline" or "ecotone" or "habitat diversity") or ("warming" or "cooling" or "temperature" or "temperatures" or "warm period" or "cold period" or "summer warmth") or ("hydroclimate" or "precipitation") or ("pal$eoclimat*" or "past climate" or "climate of the past") or "lake level" or ("vegetation productivity" or "plant productivity" or "radial growth" or "annual growth" or "growth rate" or "fuel load") or ("climate variable" or "climate interpretation" or "proxy climate record") or ("snow cover" or "snow extent") or ("organic carbon release" or "permafrost carbon" or "soil carbon release") or ("sea ice extent" or "relative sea level" or "sea level rise") or ("hunting" or "husbandry" or "domestication" or "construction activity" or "settlement history")).ab. or ("biodiversity" or ("diversity" or "evenness") or ("richness" or "number of species") or ("composition" or "assemblage" or "pal$eocommunit*" or "compositional shift" or "species dominance") or ("presence" or "present" or "absence" or "absent" or "occurrence" or "occurred") or ("stability" or "persistence" or "persisted" or "disappeared" or "extinction") or "abundance" or ("succession" or "vegetation development" or "plant cover" or "zonation pattern") or ("distribution" or "distributed" or "distributional") or ("coloni?ed" or "coloni?ation" or "established" or "spread to") or ("northern limit" or "northern treeline" or "ecotone" or "habitat diversity") or ("warming" or "cooling" or "temperature" or "temperatures" or "warm period" or "cold period" or "summer warmth") or ("hydroclimate" or "precipitation") or ("pal$eoclimat*" or "past climate" or "climate of the past") or "lake level" or ("vegetation productivity" or "plant productivity" or "radial growth" or "annual growth" or "growth rate" or "fuel load") or ("climate variable" or "climate interpretation" or "proxy climate record") or ("snow cover" or "snow extent") or ("organic carbon release" or "permafrost carbon" or "soil carbon release") or ("sea ice extent" or "relative sea level" or "sea level rise") or ("hunting" or "husbandry" or "domestication" or "construction activity" or "settlement history")).br.
