# Supplementary material for: What evidence exists for temporal variability in Arctic terrestrial and freshwater biodiversity throughout the Holocene? A systematic map protocol
Source: Environ Evid. 2022 Apr 4;11:13. doi: 10.1186/s13750-022-00267-x (PMC11378824; doi:10.1186/s13750-022-00267-x)
Supplement: Supplementary file 4 — Additional file 4: Appendix D. ‘Test list’ of sources used for search string calibration and development. [file 13750_2022_267_MOESM4_ESM.docx]

**Appendix D: ‘test list’ used for search string calibration / development**

The test list of 100 published items is given below:

| Abbott, M. B., Finney, B. P., Edwards, M. E., & Kelts, K. R. (2000). Lake-level reconstructions and paleohydrology of Birch Lake, Central Alaska, based on seismic reflection profiles and core transects. *Quaternary Research*, *53*(2), 154-166. |
| --- |
| Abbott, R. J. & Brochmann, C. History and evolution of the arctic flora: in the footsteps of Eric Hultén. *Molecular Ecology* 12, 299-313, doi:10.1046/j.1365-294X.2003.01731.x (2003). |
| Alsos, I. G. *et al.* The role of sea ice for vascular plant dispersal in the Arctic. *Biology Letters* 12, 20160264, doi:10.1098/rsbl.2016.0264 (2016). |
| Anderson, D. G. *et al.* Animal domestication in the high Arctic: Hunting and holding reindeer on the I͡Amal peninsula, northwest Siberia. *Journal of Anthropological Archaeology* 55, 101079, doi:10.1016/j.jaa.2019.101079 (2019). |
| Anderson, M., Brubaker, B., 1994. Vegetation history of northcentral Alaska: a mapped summary of late-Quaternary pollen data. Quaternary Science Reviews. |
| Andreev, A. A., Schirrmeister, L., Siegert, C., Bobrov, A. A., Demske, D., Seiffert, M., & Hubberten, H. W. (2000). Paleoenvironmental changes in Northeastern Siberia during the Late Quaternary -Evidence from pollen records of the Bykovsky Peninsula. Polarforschung, 70(1-2), 13-25. |
| Andreu-Hayles, L. *et al.* Varying boreal forest response to Arctic environmental change at the Firth River, Alaska. *Environmental Research Letters* 6, 045503, doi:10.1088/1748-9326/6/4/045503 (2011). |
| Arctic Holocene proxy climate database – new approaches to assessing geochronological accuracy and encoding climate variables. |
| Ashworth, A. C. (1996). The response of arctic Carabidae (Coleoptera) to climate change based on the fossil record of the Quaternary period. Annales Zoologici Fennici, 33(1), 125-131. |
| Bartlein, P. J. *et al.* Early-Holocene warming in Beringia and its mediation by sea-level and vegetation changes. *Clim. Past* 11, 1197-1222, doi:10.5194/cp-11-1197-2015 (2015). |
| Bellemain, E., Davey, M. L., Kauserud, H., Epp, L. S., Boessenkool, S., Coissac, E., Geml, J., Edwards, M., Willerslev, E., Gussarova, G., Taberlet, P., & Brochmann, C. (2013). Fungal palaeodiversity revealed using high-throughput metabarcoding of ancient DNA from arctic permafrost. Environmental Microbiology, 15(4), 1176-1189. |
| [Belt, S. T. et al. Striking similarities in temporal changes to spring sea ice occurrence across the central Canadian Arctic Archipelago over the last 7000 years. Quaternary Science Reviews 29, 3489-3504, doi:http://dx.doi.org/10.1016/j.quascirev.2010.06.041 (2010).](http://dx.doi.org/10.1016/j.quascirev.2010.06.041) |
| Bigelow, N. H. *et al.* Climate change and Arctic ecosystems: 1. Vegetation changes north of 55°N between the last glacial maximum, mid-Holocene, and present. *J. Geophys. Res.* 108, 8170, doi:10.1029/2002jd002558 (2003). |
| Birks, H. H. (2008). The Late-Quaternary history of arctic and alpine plants. Plant Ecology & Diversity, 1(2), 135-146. http://www.informaworld.com/smpp/title~content=t793409773~db=all |
| [Birks, HJB, Vivian A Felde, and Alistair WR Seddon. ‘Biodiversity Trends within the Holocene’. The Holocene 26, no. 6 (1 June 2016): 994–1001. https://doi.org/10.1177/0959683615622568.](https://doi.org/10.1177/0959683615622568) |
| Bjune, A. E., Bakke, J., Nesje, A. & Birks, H. J. B. Holocene mean July temperature and winter precipitation in western Norvay inferred from palynological and glaciological lake-sediment proxies. *The Holocene* 15, 177-189 (2005). |
| [Briffa, K. R. et al. Reassessing the evidence for tree-growth and inferred temperature change during the Common Era in Yamalia, northwest Siberia. Quaternary Science Reviews 72, 83-107, doi:http://dx.doi.org/10.1016/j.quascirev.2013.04.008 (2013).](http://dx.doi.org/10.1016/j.quascirev.2013.04.008) |
| Brubaker LB, Anderson PM, Hu FS, 1995. Arctic tundra biodiversity: a temporal perspective from late quaternary pollen record. In: Chapin FS, Ko€rner C (eds), Arctic and Alpine Biodiversity. Springer Verlag, Berlin, pp. 111e125. |
| [Büntgen, U. et al. Long-term recruitment dynamics of arctic dwarf shrub communities in coastal east Greenland. Dendrochronologia 50, 70-80, doi:https://doi.org/10.1016/j.dendro.2018.05.005 (2018).](https://doi.org/10.1016/j.dendro.2018.05.005) |
| Büntgen, U. *et al.* Temperature-induced recruitment pulses of Arctic dwarf shrub communities. *Journal of Ecology* 103, 489-501, doi:10.1111/1365-2745.12361 (2015). |
| Caccianiga, M. & Payette, S. Recent advance of white spruce (Picea glauca) in the coastal tundra of the eastern shore of Hudson Bay (Quebec, Canada). *Journal Of Biogeography* 33, 2120-2135 (2006). |
| Chapin, F. S., Bret-Harte, M. S., Hobbie, S. E. & Zhong, H. Plant functional types as predictors of transient responses of arctic vegetation to global change. *Journal of Vegetation Science* 7, 347-358, doi:10.2307/3236278 (1996). |
| Clarke, C. L. *et al.* Holocene floristic diversity and richness in northeast Norway revealed by sedimentary ancient DNA (sedaDNA) and pollen. *Boreas* 0, doi:10.1111/bor.12357 (2018). |
| Clarke, C. L. *et al.* Persistence of arctic-alpine flora during 24,000 years of environmental change in the Polar Urals. *Scientific Reports* 9, 19613, doi:10.1038/s41598-019-55989-9 (2019). |
| [Clarke, C. L., Alsos, I. G., Edwards, M. E., Paus, A., Gielly, L., Haflidason, H., Mangerud, J., Regnéll, C., Hughes, P. D. M., Svendsen, J. I., & Bjune, A. E. (2020). A 24,000-year ancient DNA and pollen record from the Polar Urals reveals temporal dynamics of arctic and boreal plant communities. Quaternary Science Reviews, 247, 106564. https://doi.org/10.1016/j.quascirev.2020.106564](https://doi.org/10.1016/j.quascirev.2020.106564) |
| Colinvaux, P.A., 1967. Quaternary vegetational history of Arctic Alaska, p. 207-231. In D.M. Hopkins, ed., The Bering Land Bridge. Stanford University Press, Stanford, 495 p. |
| Cwynar, L. C. (1982). A LATE QUATERNARY VEGETATION HISTORY FROM HANGING LAKE NORTHERN YUKON CANADA. Ecological Monographs, 52(1), 1-24. https://doi.org/10.2307/2937342 |
| [Dalton, A. S., Finkelstein, S. A., Barnett, P. J., Väliranta, M. & Forman, S. L. Late Pleistocene chronology, palaeoecology and stratigraphy at a suite of sites along the Albany River, Hudson Bay Lowlands, Canada. Palaeogeography, Palaeoclimatology, Palaeoecology 492, 50-63, doi:https://doi.org/10.1016/j.palaeo.2017.12.011 (2018).](https://doi.org/10.1016/j.palaeo.2017.12.011) |
| Dalton, A. S., Väliranta, M., Barnett, P. J. & Finkelstein, S. A. Pollen and macrofossil-inferred palaeoclimate at the Ridge Site, Hudson Bay Lowlands, Canada: evidence for a dry climate and significant recession of the Laurentide Ice Sheet during Marine Isotope Stage 3. *Boreas* 46, 388-401, doi:10.1111/bor.12218 (2016). |
| Danby, R. K. & Hik, D. S. Variability, contingency and rapid change in recent subarctic alpine tree line dynamics. *Journal of Ecology* 95, 352-363, doi:10.1111/j.1365-2745.2006.01200.x (2007). |
| Dearborn, K. D. & Danby, R. K. Topographic influences on ring widths of trees and shrubs across alpine treelines in southwest Yukon. *Arctic, Antarctic, and Alpine Research* 50, e1495445, doi:10.1080/15230430.2018.1495445 (2018). |
| Dornelas, M. *et al.* BioTIME: A database of biodiversity time series for the Anthropocene. *Global Ecology and Biogeography* 27, 760-786, doi:10.1111/geb.12729 (2018). |
| Douglas, M. S. V., & Smol, J. P. (2010). Freshwater diatoms as indicators of environmental change in the High Arctic. The diatoms: applications for the environmental and earth sciences(Ed.2), 249-266. |
| Dyke, A. S., England, J., Reimnitz, E. & Jette, H. Changes in driftwood delivery to the Canadian arctic archipelago: The hypothesis of postglacial oscillations of the transpolar drift. *Arctic* 50, 1-16 (1997). |
| Dyke, A. S., Savelle, J. M. & Johnson, D. S. Paleoeskimo Demography and Holocene Sea-level History, Gulf of Boothia, Arctic Canada. *Arctic* 64, 151-168, doi:10.14430/arctic4096 (2011). |
| Edwards, M. E. et al. Pollen-based biomes for Beringia 18,000, 6000 and 0 14C yr bp. Journal of Biogeography 27, 521-554, doi:10.1046/j.1365-2699.2000.00426.x (2000). |
| Edwards, M. E., Armbruster, W. S. & Elias, S. E. Constraints on post-glacial boreal tree expansion out of far-northern refugia. Global Ecology and Biogeography 23, 1198-1208, doi:10.1111/geb.12213 (2014). |
| Edwards, M. E., Brubaker, L. B., Lozhkin, A. V. & Anderson, P. M. Structurally novel biomes: A response to past warming in Beringia. *Ecology* 86, 1696-1703 (2005). |
| [Edwards, M. E., Mock, C. J., Finney, B. P., Barber, V. A. & Bartlein, P. J. Potential analogues for paleoclimatic variations in eastern interior Alaska during the past 14,000 yr: atmospheric-circulation controls of regional temperature and moisture responses. Quaternary Science Reviews 20, 189-202, doi:http://dx.doi.org/10.1016/S0277-3791(00)00123-2 (2001).](http://dx.doi.org/10.1016/S0277-3791(00)00123-2) |
| Eidesen, P. B. *et al.* Genetic roadmap of the Arctic: plant dispersal highways, traffic barriers and capitals of diversity. *New Phytologist* 200, 898-910, doi:10.1111/nph.12412 (2013). |
| Eidesen, P. B., Carlsen, T., Molau, U. & Brochmann, C. Repeatedly out of Beringia: Cassiope tetragona embraces the Arctic. *Journal of Biogeography* 34, 1559-1574 (2007). |
| Elmslie, B. G., Gushulak, C. A. C., Boreux, M. P., Lamoureux, S. F., Leavitt, P. R., & Cumming, B. F. (2020). Complex responses of phototrophic communities to climate warming during the Holocene of northeastern Ontario, Canada. Holocene, 30(2), 272-288. |
| Engelstad, E. (1985). THE LATE STONE AGE OF ARCTIC NORWAY A REVIEW. Arctic Anthropology, 22(1), 79-96. |
| Epstein, H. E. *et al.* The nature of spatial transitions in the Arctic. *Journal of Biogeography* 31, 1917-1933, doi:10.1111/j.1365-2699.2004.01140.x (2004). |
| Fastie, C. L. Causes and Ecosystem Consequences of Multiple Pathways of Primary Succession at Glacier Bay, Alaska. *Ecology* 76, 1899-1916, doi:10.2307/1940722 (1995). |
| Felde, V. A., Grytnes, J.-A., Bjune, A. E., Peglar, S. M. & Birks, H. J. B. Are diversity trends in western Scandinavia influenced by post-glacial dispersal limitation? *Journal of Vegetation Science* 29, 360-370, doi:10.1111/jvs.12569 (2017). |
| Gaglioti, B. V. *et al.* Radiocarbon age-offsets in an arctic lake reveal the long-term response of permafrost carbon to climate change. *Journal of Geophysical Research: Biogeosciences* 119, 2014JG002688, doi:10.1002/2014JG002688 (2014). |
| [Gaglioti, B. V. et al. Younger-Dryas cooling and sea-ice feedbacks were prominent features of the Pleistocene-Holocene transition in Arctic Alaska. Quaternary Science Reviews 169, 330-343, doi:https://doi.org/10.1016/j.quascirev.2017.05.012 (2017).](https://doi.org/10.1016/j.quascirev.2017.05.012) |
| Gajewski, K., Viau, A., Sawada, M., Atkinson, D. & Wilson, S. Sphagnum peatland distribution in North America and Eurasia during the past 21,000 years. *Global Biogeochemical Cycles* 15, 297-310, doi:10.1029/2000GB001286 (2001). |
| Galetti, M. *et al.* Ecological and evolutionary legacy of megafauna extinctions. *Biological Reviews*, (in press), doi:10.1111/brv.12374 (2017). |
| Giesecke, T. & Bennett, K. D. The Holocene spread of Picea abies (L.) Karst. in Fennoscandia and adjacent areas. *Journal of Biogeography* 31, 1523-1548, doi:10.1111/j.1365-2699.2004.01095.x (2004). |
| Girardin, M. P. *et al.* Unusual forest growth decline in boreal North America covaries with the retreat of Arctic sea ice. *Global Change Biology* 20, 851-866, doi:10.1111/gcb.12400 (2014). |
| Haile, J., Froese, Duane G. & MacPhee, Ross D. E. Ancient DNA reveals late survival of mammoth and horse in interior Alaska. *Proceedings of the National Academy of Sciences of the United States of America* 106, 22352-22357 (2009). |
| Helama, S. *et al.* Late Holocene climatic variability reconstructed from incremental data from pines and pearl mussels – a multi-proxy comparison of air and subsurface temperatures. *Boreas* 39, 734-748, doi:10.1111/j.1502-3885.2010.00165.x (2010). |
| [Hellmann, L. et al. Dendro-provenancing of Arctic driftwood. Quaternary Science Reviews 162, 1-11, doi:http://dx.doi.org/10.1016/j.quascirev.2017.02.025 (2017).](http://dx.doi.org/10.1016/j.quascirev.2017.02.025) |
| [Hellmann, L. et al. Regional coherency of boreal forest growth defines Arctic driftwood provenancing. Dendrochronologia 39, 3-9, doi:http://dx.doi.org/10.1016/j.dendro.2015.12.010 (2016).](http://dx.doi.org/10.1016/j.dendro.2015.12.010) |
| Higuera, P. E. et al. 2008. Frequent fires in ancient shrub tundra: implications of paleorecords for Arctic environmental change. |
| Higuera, P. E. et al. 2009. Vegetation mediated the impacts of postglacial climate change on fire regimes in the south-central Brooks Range, Alaska. – Ecol. Monogr. 79: 201–219. – PLoS One 3: e0001744. |
| Hu, F.S., Finney, B.P. & Brubaker, L.B., 2001. Effects of Holocene Alnus Expansion on Aquatic Productivity, Nitrogen Cycling, and Soil Development in Southwestern Alaska. *Ecosystems*, 4(4), pp.358–368. |
| Jakobsson, M., Long, A., Ingólfsson, Ó., Kjær, K. H. & Spielhagen, R. F. New insights on Arctic Quaternary climate variability from palaeo-records and numerical modelling. *Quaternary Science Reviews* 29, 3349-3358, doi:10.1016/j.quascirev.2010.08.016 (2010). |
| Kaakinen A, Eronen M (2000) Holocene pollen stratigraphy indicating climatic and tree-line changes derived from a peat section at Ortino, in the Pechora lowland, northern Russia. The Holocene, 10, 611–620. |
| Kapfer, J. & Popova, K. Changes in subarctic vegetation after one century of land use and climate change. *Journal of Vegetation Science* n/a, doi:10.1111/jvs.12854 (2019). |
| Kaplan, J. O. *et al.* Climate change and Arctic ecosystems: 2. Modeling, paleodata-model comparisons, and future projections. *J. Geophys. Res.* 108, 8171, doi:10.1029/2002jd002559 (2003). |
| Kaufman, D. S. *et al.* Holocene thermal maximum in the western Arctic (0–180°W). *Quaternary Science Reviews* 23, 529-560, doi:10.1016/j.quascirev.2003.09.007 (2004). |
| [Kienast, F. et al. Paleontological records indicate the occurrence of open woodlands in a dry inland climate at the present-day Arctic coast in western Beringia during the Last Interglacial. Quaternary Science Reviews 30, 2134-2159, doi:https://doi.org/10.1016/j.quascirev.2010.11.024 (2011).](https://doi.org/10.1016/j.quascirev.2010.11.024) |
| [Kinnard, C. et al. Reconstructed changes in Arctic sea ice over the past 1,450 years. Nature 479, 509-512, doi:http://www.nature.com/nature/journal/v479/n7374/abs/nature10581.html#supplementary-information (2011).](http://www.nature.com/nature/journal/v479/n7374/abs/nature10581.html#supplementary-information) |
| Kremenetski, C. V., Sulerzhitsky, L. D. & Hantemirov, R. Holocene History of the Northern Range Limits of Some Trees and Shrubs in Russia. *Arctic and Alpine Research* 30, 317-333 (1998). |
| Kullman, L. & Kjallgren, L. Holocene pine tree-line evolution in the Swedish Scandes: Recent tree-line rise and climate change in a long-term perspective. *Boreas* 35, 159-168, doi:10.1080/03009480500359137 (2006). |
| Kullman, L. Holocene History of the Forest Alpine Tundra Ecotone in the Scandes Mountains (Central Sweden). *New Phytologist* 108, 101-110, doi:DOI 10.1111/j.1469-8137.1988.tb00209.x (1988). |
| Kullman, L. Holocene Tree-Limit and Climate History from the Scandes Mountains, Sweden. *Ecology* 76, 2490-2502, doi:Doi 10.2307/2265823 (1995). |
| Kullman, L. Late Holocene Reproductional Patterns of *Pinus sylvestris* and *Picea abies* at the Forest Limit in Central Sweden. *Canadian Journal of Botany* 64, 1682-1690, doi:DOI 10.1139/b86-225 (1986). |
| Kullman, L. Non-analogous tree flora in the Scandes Mountains, Sweden, during the early Holocene - macrofossil evidence of rapid geographic spread and response to palaeoclimate. *Boreas* 27, 153-161 (1998). |
| Kuzmina, S., Froese, D. G., Jensen, B. J. L., Hall, E., & Zazula, G. D. (2014). Middle Pleistocene (MIS 7) to Holocene fossil insect assemblages from the Old Crow basin, northern Yukon, Canada. Quaternary International, 341, 216-242. |
| [Liu, S., Stoof-Leichsenring, K. R., Kruse, S., Pestryakova, L. A., & Herzschuh, U. (2020). Holocene Vegetation and Plant Diversity Changes in the North-Eastern Siberian Treeline Region From Pollen and Sedimentary Ancient DNA. Frontiers in Ecology and Evolution, 8. https://doi.org/10.3389/fevo.2020.560243](https://doi.org/10.3389/fevo.2020.560243) |
| Lydolph, M. C., Jacobsen, J., Arctander, P., Thomas, M., Gilbert, P., Gilichinsky, D. A., Hansen, A. J., Willerslev, E., & Lange, L. (2005). Beringian paleoecology inferred from permafrost-preserved fungal DNA. Applied and Environmental Microbiology, 71(2), 1012-1017. https://doi.org/http://dx.doi.org/10.1128/AEM.71.2.1012-1017.2005 |
| McFarlin, J. M., Axford, Y., Osburn, M. R., Kelly, M. A., Osterberg, E. C., & Farnsworth, L. B. (2018). Pronounced summer warming in northwest Greenland during the Holocene and Last Interglacial. Proceedings of the National Academy of Sciences of the United States of America, 115(25), 6357-6362. https://doi.org/10.1073/pnas.1720420115 |
| Meiri, M., Lister, A., Kosintsev, P., Zazula, G., & Barnes, I. (2020). Population dynamics and range shifts of moose (Alces alces) during the Late Quaternary. *Journal of Biogeography*, *47*(10), 2223-2234. |
| Omurova, G. T., Seim, A., Barinov, V. V., Kardash, O. V., & Myglan, V. S. (2020). Construction history and timber use of the medieval settlement Nadymskiy Gorodok in the northwestern Siberian forest-tundra. *Journal of Archaeological Science*, *116*. |
| Palagushkina, O. V., Wetterich, S., Schirrmeister, L., & Nazarova, L. B. (2017). Modern and fossil diatom assemblages from Bol’shoy Lyakhovsky Island (New Siberian Archipelago, Arctic Siberia). Contemporary Problems of Ecology, 10(4), 380-394. |
| Pitulko, V. V., Ivanova, V. V., Kasparov, A. K. & Pavlova, E. Y. Reconstructing prey selection, hunting strategy and seasonality of the early Holocene frozen site in the Siberian High Arctic: A case study on the Zhokhov site faunal remains, De Long Islands. *Environmental Archaeology* 20, 120-157, doi:10.1179/1749631414Y.0000000040 (2015). |
| Rolland, N., Larocque, I., Francus, P., Pienitz, R., & Laperrière, L. (2008). Holocene climate inferred from biological (Diptera: Chironomidae) analyses in a Southampton Island (Nunavut, Canada) lake. Holocene, 18(2), 229-241. |
| Schmidt, N. M., Baittinger, C. & Forchhammer, M. C. Reconstructing century-long snow regimes using estimates of high arctic Salix arctica radial growth. *Arctic Antarctic and Alpine Research* 38, 257-262 (2006). |
| Schwert DP, Ashworth AC. LATE QUATERNARY HISTORY OF THE NORTHERN BEETLE FAUNA OF NORTH AMERICA: A SYNTHESIS OF FOSSIL AND DISTRIBUTIONAL EVIDENCE. The Memoirs of the Entomological Society of Canada 120: 93–107. |
| Seppä, H. & Birks, H. J. B. July mean temperature and annual precipitation trends during the Holocene in the Fennoscandian tree-line area: pollen-based climate reconstructions. *The Holocene* 11, 527-539 (2001). |
| Seppä, H. *et al.* Invasion of Norway spruce (Picea abies) and the rise of the boreal ecosystem in Fennoscandia. *Journal of Ecology* 97, 629-640, doi:10.1111/j.1365-2745.2009.01505.x (2009). |
| Seppä, H., MacDonald, G. M., Birks, H. J. B., Gervais, B. R. & Snyder, J. A. Late-Quaternary summer temperature changes in the northern-European tree-line region. *Quaternary Research* 69, 404-412 (2008). |
| Shapiro, B. *et al.* Rise and Fall of the Beringian Steppe Bison. *Science* 306, 1561 (2004). |
| Skirbekk, K., Kristensen, D. K., Rasmussen, T. L., Koç, N. & Forwick, M. Holocene climate variations at the entrance to a warm Arctic fjord: evidence from Kongsfjorden trough, Svalbard. *Geological Society, London, Special Publications* 344, 289-304 (2010). |
| Smol, J. P., & Douglas, M. S. V. (2007). Crossing the final ecological threshold in high Arctic ponds. Proceedings of the National Academy of Sciences of the United States of America, 104(30), 12395-12397. https://doi.org/http://dx.doi.org/10.1073/pnas.0702777104 |
| Stewart, J. R. & Lister, A. M. Cryptic northern refugia and the origins of the modern biota. *Trends in Ecology & Evolution* 16, 608-613, doi:10.1016/S0169-5347(01)02338-2 (2001). |
| Stewart, T. G. & England, J. Holocene Sea-Ice Variations and Paleoenvironmental Change, Northernmost Ellesmere Island, N.W.T., Canada. *Arctic and Alpine Research* 15, 1-17 (1983). |
| Streeter R, Dugmore AJ, Lawson IT, Erlendsson E, Edwards KJ. 2015. The onset of the palaeoanthropocene in Iceland: Changes in complex natural systems:. The Holocene. |
| Sundqvist, H. S. *et al.* Arctic Holocene proxy climate database - new approaches to assessing geochronological accuracy and encoding climate variables. *Clim. Past* 10, 1605-1631, doi:10.5194/cp-10-1605-2014 (2014). |
| Treat, C. C., M. C. Jones, P. Camill, A. Gallego-Sala, M. Garneau, J. W. Harden, G. Hugelius, et al. ‘Effects of Permafrost Aggradation on Peat Properties as Determined from a Pan-Arctic Synthesis of Plant Macrofossils’. Journal of Geophysical Research: Biogeosciences 121, no. 1 (2016): 78–94. https://doi.org/10.1002/2015JG003061. |
| Voldstad, L. H., Alsos, I. G., Farnsworth, W. R., Heintzman, P. D., Håkansson, L., Kjellman, S. E., Rouillard, A., Schomacker, A., & Eidesen, P. B. (2020). A complete Holocene lake sediment ancient DNA record reveals long-standing high Arctic plant diversity hotspot in northern Svalbard. Quaternary Science Reviews, 234, 106207. https://doi.org/10.1016/j.quascirev.2020.106207 |
| Willerslev, E. *et al.* Fifty thousand years of Arctic vegetation and megafaunal diet. *Nature* 506, 47-51, doi:10.1038/nature12921 (2014). |
| Willerslev, E., Hansen, A. J., Christensen, B., Steffensen, J. P., & Arctander, P. (1999). Diversity of Holocene life forms in fossil glacier ice. Proceedings of the National Academy of Sciences of the United States of America, 96(14), 8017-8021. |
| Wilson, M. J., & Elias, S. A. (1986). PALEOECOLOGICAL SIGNIFICANCE OF HOLOCENE INSECT FOSSIL ASSEMBLAGES FROM THE NORTH COAST OF ALASKA USA. Arctic, 39(2), 150-157. |
| Zhang, H. *et al.* Inconsistent Response of Arctic Permafrost Peatland Carbon Accumulation to Warm Climate Phases. *Global Biogeochemical Cycles* 32, 1605-1620, doi:10.1029/2018GB005980 (2018). |
| Zinovjev, E. V. (2005). Early holocene entomocomplexes from the middle reaches of the Ob' river in West Siberia [Материалы к характеристике раннеголоценовых энтомокомплексов Среднего Приобья]. Evraziatskii entomologicheskii zhurnal, 4(4), 283-292. |
