# Supplementary material for: What evidence exists for temporal variability in Arctic terrestrial and freshwater biodiversity throughout the Holocene? A systematic map protocol
Source: Environ Evid. 2022 Apr 4;11:13. doi: 10.1186/s13750-022-00267-x (PMC11378824; doi:10.1186/s13750-022-00267-x)
Supplement: Supplementary file 5 — Additional file 5. ROSES Checklist. [file 13750_2022_267_MOESM5_ESM.pdf]

Section / sub-section	Topic	Description	Further explanation	Checklist/Meta-data	Author response	Comments
Title	Title	The title must indicate that it is a systematic map protocol, and must indicate if it is an update/amendment: e.g. "A systematic map update protocol...".	The title should normally be the same or very similar to the review question.	Meta-data	Title is:	What evidence exists for temporal variability in the measures of Arctic terrestrial and freshwater biodiversity throughout the Holocene? A systematic map protocol.
Type of review	Type of review	Select one of the following types of review: systematic map, systematic	See CEE Guidance on systematic mapping [1], and on amendments and	Meta-data	systematic map	
Authors contacts	Authors contacts	The full names, institutional addresses, and email addresses for all		Checklist	Yes	
Abstract	Structured summary	Abstract must not exceed 350 words and must include two sections 1)		Checklist	Yes	
Background	Background	Describe the rationale for the review in the context of what is already	A theory of change and/or conceptual model can be presented that links	Checklist	Yes	
Stakeholder engagement	Stakeholder engagement	The planned/actual role of stakeholders throughout the review process		Checklist	Yes	
Objective of the review	Objective	Describe the primary question and secondary questions (when	The primary question is the main question of the review. Secondary	Checklist	Yes	
	Definitions of the question components	Break down and summarise question key elements e.g. population, intervention(s)/exposure(s), comparator(s), and outcome(s).	For other question types see [4,5]	Meta-data	PECO	Population (Arctic biota); Exposure (year in Holocene); Comparator (another year in Holocene); Outcome (measure of biodiversity)
Methods						
Searches	Search strategy		Details regarding search strategy testing should be provided.	Checklist	Yes	
	Search string	Provide Boolean-style full search string and state the platform for		Meta-data	Scopus	See below
	Languages – bibliographic	List languages to be used in bibliographic database searches.		Meta-data	English, Russian,	
	Languages – grey literature	List languages to be used in organizational websites searches and web-		Meta-data	English,	
	Bibliographic databases	Provide the number of bibliographic databases to be searched.		Meta-data	13	
	Web – based search engines	Provide the number of web – based search engines to be searched.		Meta-data	2	
	Organisational websites	Provide the number of organisational websites to be searched.		Meta-data	9	
	Estimating the	Describe the process by which the comprehensiveness of the search		Checklist	Yes	
	Search update	Describe any plans to update the searches during the conduct of the	Optional. A search update is good practice if original searches were	Checklist	Yes	
Article screening and study	Screening strategy	Describe the methodology for screening articles/studies for		Checklist	Yes	
	Consistency checking	Describe clearly the process for checking consistency of decisions		Checklist	Yes	
	Inclusion criteria	Describe the inclusion criteria used to assess relevance of identified		Checklist	Yes	see Table 2
	Reasons for exclusion	State that you will provide a list of articles excluded at full text with		Checklist	Yes	
Critical appraisal	Critical appraisal strategy	Describe here the method you propose for critical appraisal of study	Optional	Checklist	Yes	Time-series harmonisation
	Critical appraisal used in	Describe how the information from critical appraisal will be used in	Optional	Checklist	Yes	
	Consistency checking	Describe how repeatability of critical appraisal of study validity will be	Optional	Checklist	No	
Data extraction	Meta-data extraction and coding	Describe the method for meta-data extraction and coding for studies		Checklist	Yes	
Data synthesis and	Narrative synthesis strategy	Describe methods to be used for narratively synthesising the evidence	Vote-counting (tallying of studies based on the direction or significance	Checklist	Yes	
	Knowledge gap and cluster	Describe the methods to be used to identify and/or prioritise key		Checklist	Yes	
	Demonstrating procedural	Describe the role of systematic reviewers (who have also authored	Reviewers who have authored articles to be considered within the	Checklist	Yes	
Declarations	Competing interests	Describe of any financial or non-financial competing interests that the		Checklist	Yes	

References

[1] James, K.L., Randall, N.P. and Haddaway, N.R., 2016. A methodology for systematic mapping in environmental sciences. Environmental Evidence, 5(1), p.7.
[2] Bayliss, H.R., Haddaway, N.R., Eales, J., Frampton, G.K. and James, K.L., 2016. Updating and amending systematic reviews and systematic maps in environmental management. Environmental Evidence, 5(1), p.20.
[3] Haddaway, N.R., Kohl, C., da Silva, N.R., Schiemann, J., Spök, A., Stewart, R., Sweet, J.B. and Wilhelm, R., 2017. A framework for stakeholder engagement during systematic reviews and maps in environmental management. *Environmental Evidence* , *6* (1), p.11.
[4] Collaboration for Environmental Evidence. 2018. Guidelines and Standards for Evidence synthesis in Environmental Management. Version 5.0. www.environmentalevidence.org/information-for-authors.
[5] Leeds Institute of Health Sciences. https://medhealth.leeds.ac.uk/info/639/information_specialists/1500/search_concept_tools. Accessed 12/11/2017.

TITLE-ABS-KEY((("biodiversity") OR ("diversity" OR "evenness") OR ("richness" OR "number of species") OR ("composition" OR "assemblage" OR "pal*eocommunit*" OR "compositional shift" OR "species dominance") OR ("presence" OR "present" OR "absence" OR "absent" OR "occurrence" OR "occurred") OR ("stability" OR "persistence" OR "persisted" OR "disappeared" OR "extinction") OR ("abundance") OR ("succession" OR "vegetation development" OR "plant cover" OR "zonation pattern") OR ("distribution" OR "distributed" OR "distributional") OR ("coloni?ed" OR "coloni?ation" OR "established" OR "spread to") OR ("northern limit" OR "northern treeline" OR "ecotone" OR "habitat diversity") OR ("warming" OR "cooling" OR "temperature" OR "temperatures" OR "warm period" OR "cold period" OR "summer warmth") OR ("hydroclimate" OR "precipitation") OR ("pal*eoclimat*" OR "past climate" OR "climate of the past") OR ("lake level") OR ("vegetation productivity" OR "plant productivity" OR "radial growth" OR "annual growth" OR "growth rate" OR "fuel load") OR ("climate variable" OR "climate interpretation" OR "proxy climate record") OR ("snow cover" OR "snow extent") OR ("organic carbon release" OR "permafrost carbon" OR "soil carbon release") OR ("sea ice extent" OR "relative sea level" OR "sea level rise") OR ("hunting" OR "husbandry" OR "domestication" OR "construction activity" OR "settlement history")
) AND (("arctic" OR "high-latitude" OR "oro-arctic" OR "North Polar region" OR "subarctic" OR "northern environ*") OR ("Canada" OR "Canadian" OR "Russia" OR "Russian Federation" OR "USSR" OR "RSFSR" OR "Russian Federation" OR "Russian" OR "Norway" OR "Norwegian" OR "Finland" OR "Finnish" OR "Sweden" OR "Iceland" OR "Icelandic" OR "Greenland" OR "Greenlandic" OR "Faroe") OR ("tundra" OR "permafrost" OR "mammoth steppe") OR ("Yukon Territory" OR "Northwest Territories" OR "Nunavut" OR "Baffin Island" OR "Belcher Islands" OR "Ellesmere Island" OR "Southampton Island" OR "Mackenzie River" OR "Great Slave Lake" OR "Great Bear Lake" OR "Yukon River" OR "Alaska" OR "Teshekpuk Lake" OR "Queen Elizabeth Islands" OR "Victoria Island" OR "Banks Island" OR "Wrangel Island" OR "Seward peninsula" OR "Hudson Bay") OR ("Siberia" OR "Siberian" OR "Lake Baikal" OR "Lena River" OR "New Siberian Islands" OR "Severnaya Zemlya" OR "Franz Josef Land" OR "Novaya Zemlya" OR "Chukotka Autonomous Okrug" OR "Kamchatka Krai" OR "Magadan Oblast" OR "Murmansk Oblast" OR "Sakha" OR "Arkhangelsk Oblast" OR "Irkutsk Oblast" OR "Khabarovsk Krai" OR "Komi Republic" OR "Krasnoyarsk Krai" OR "Republic of Karelia" OR "Sakhalin Oblast" OR "Tuva" OR "Tyumen Oblast" OR "Polar Urals" OR "Yamalia") OR ("Svalbard" OR "Spitsbergen" OR "Bjørnøya" OR "Jan Mayen" OR "Finnmark" OR "Troms" OR "Lapland" or "Lappi" OR "Grímsey" OR "Lappland" OR "Norrbotten" OR "Västerbotten" OR "North West Europe" OR "Northwest Europe" OR "north* Europe" OR "Scandes" OR "Kola Peninsula") OR ("beringia" OR "beringian") OR ("global data*")
) AND (("reconstruct" OR "reconstruction" OR "reconstructed") OR ("pal*eo*" OR "micropal*entol*") OR ("arch*eolog*" OR "artefact") OR ("history" OR "historic site" OR "historical record") OR ("dendrochron*" OR "dendroclim*" OR "dendroeco*" OR "growth ring" OR "tree ring" OR "wood ring") OR ("radiocarbon" OR "radio-carbon" OR "AMS" OR "accelerator mass spectrometry") OR ("age determination" OR "years before present" OR "yr BP" OR "calibration of age" OR "age model" OR "age-depth model") OR ("1?,??? 14C yr B*P" OR "1? ??? 14C Yr B*P" OR "1?,??? yr B*P" OR "1? ??? yr B*P" OR "1?,??? cal yr B*P" OR "1? ??? cal yr B*P" OR "pre-1???" OR "pre-20th century" OR "1?th century" OR "last ?,??? years" OR "last 1?,??? years" OR "14C dates" OR "1? Cal Ka" OR "one century" OR "pre-industrial" OR "preindustrial") OR ("fossil" OR "sub-fossil" OR "macrofossil" OR "megafossil" OR "microfossil" OR "subfossil") OR ("palynolog*") OR ("prehistoric" OR "prehistory" OR "Iron Age" OR "Bronze Age" OR "Neolithic" OR "Mesolithic" OR "Late Upper Pal*eolithic" OR "BC") OR ("Holocene" OR "MidHolocene" OR "Mid-Holocene" OR "late Pleistocene" OR "end of the Pleistocene" OR "Late Quaternary" OR "Little Ice Age" OR "Medieval Climate Anomoly" OR "Younger Dryas" OR "MIS 1" OR "marine isotope stage 1") OR ("centennial" OR "centuries" OR "millenial" OR "millenia" OR "submillenial" OR "chronolog*" OR "temporal trend") OR ("preboreal chronozone" OR "boreal chronozone" OR "atlantic chronozone" OR "subboreal chronozone") OR ("sediment core" OR "sedimentary sequence" OR "lake sediment" OR "transfer function" OR "peat core" OR "peat bog core" OR "peat monolith") OR ("ancient DNA" OR "aDNA" OR "sedDNA" OR "sedimentary DNA") OR ("postglacial" OR "post-glacial" OR "deglaciation" OR "deglacial") OR ("last appearance date" OR "refugia") OR ("multiproxy" OR "proxy record" OR "proxy archive" OR "proxy" OR "proxy study" OR "proxy studies") OR ("long-term ecolog*" OR "long-term record")) AND (("plant?" OR "flora" OR "vegetation" OR "Plantae" OR "vegetational" OR "pal?eovegetation" OR "floristic") OR ("bryophyte" OR "Bryophyta" OR "liverwort?" OR "moss" OR "mosses") OR ("shrub?" OR "woody plant" OR "vascular plant" OR "fern?" OR "pteridophyt?" OR "spermatopht?" OR "xerophyte" OR "tree species" OR "coniferous" OR "conifer" OR "deciduous" OR "herb" OR "forb?") OR ("forest species" OR "treeline species" OR "tree-line species" OR "larch" OR "Larix" OR "spruce" OR "picea" OR "western hemlock" OR "Tsuga" OR "Sitka" OR "alnus" OR "alder" OR "cottonwood" OR "Populus" OR "poplar" OR "Pinus" OR "pine" OR "Betula" OR "birch" OR "willow" OR "salix") OR ("lichen?" OR "sporomiella" OR "dung fungal spore" OR "fungi" OR "fungal") OR ("diatom" OR "Bacillariophyceae") OR ("Mollusca" OR "mollusk" OR "mollusc" OR "Gastropoda" OR "gastropod" OR "Bivalvia" OR "bivalve") OR ("mammal" OR "mammalian" OR "mammoth" OR "reindeer" OR "caribou" OR "Rangifer tarandus" OR "polar bear" OR "Mammuthus primigenius" OR "steppe bison" OR "Bison priscus" OR "muskox" OR "Ovibos moschatus" OR "moose" OR "Alcos alces" OR "horse" OR "Equus caballus" OR "wolf" OR "wolves" OR "Canis lupus" OR "dog" OR "squirrel" OR "rodent" OR "Rodentia" OR "Urocitellus" OR "arctic fox" OR "vulpes lagopus" OR "alopex lagopus" OR "reptile" OR "bird?" OR "Aves") OR ("ostracod" OR "ostracode" OR "ostracoda") OR ("coleoptera" OR "beetle") OR ("chironomid" OR "Chironomidae") OR ("insect" OR "macroinvertebrate" OR "macro-invertebrate") OR ("amphibian" OR "Amphibia" OR "aquatic animal" OR "algae" OR "algal") OR ("animal" OR "fauna" OR "faunal" OR "megafauna" OR "megaherbivore" OR "vertebrata" OR "vertebrate") OR ("pollen" OR "palynology" OR "macrofossil" OR "pal*eobotanical" OR "leaf wax" OR "palynomorph") OR ("organic biomarker") OR ("ring width" OR "wood ring" OR "tree ring" OR "stem sample" OR "driftwood") OR ("terrestrial proxies" OR "terrestrial proxy")))
